# Supplementary material for: Climatic niche evolution in the viviparous Sceloporus torquatus group (Squamata: Phrynosomatidae)
Source: PeerJ. 2019 Jan 9;6:e6192. doi: 10.7717/peerj.6192 (PMC6330044; doi:10.7717/peerj.6192)
Supplement: Supplemental Information 1 [file peerj-07-6192-s001.docx]

**Supplementary Analysis**

**Multivariate analysis**

**Material and Methods**

To test the evolutionary process thorough the dimensions of niche space in the *Sceloporus torquatus* group, we perform a multivariate analyses in a phylogenetic context. We used bioclim layers, monthly and annual maximum and minimum temperature and precipitation levels available from the WorldClim database 1.4 (http://www. worldclim.org), monthly and annual potential evapotranspiration (PET) and aridity data available from http://www.cgiar-csi.org/data/global-aridity-and-pet-database (Zomer *et al*., 2008). All layers were downloaded at a 30 arc second resolution and clipped to the general species group distribution. In addition, we used occurrence data for all species of the *Sceloporus torquatus* group after removing redundant occurrence points and pruning the database to allow only one record for each cell of the grid (30 arc seconds). We extracted bioclimatic values for each collecting point, transformed the data into z-scores, and then used a Principal Component Analyses (PCA to reduce the dimensionality of the data using a correlative matrix. We retained PCs using the broken-stick method, and used the resulting loadings and scores to explore the tempo and mode of niche evolution in the *S. torquatus* group. We assessed the evolutionary mode of PCs fitting the following models: (1) Brownian motion (BM); (2) Ornstein-Uhlenbeck (OU) (Butler and King 2004), (3) Early burst (EB), and (4) Pagel’s delta (δ) (Pagel, 1999). A best fitting model of evolution was identified by means of log likelihood and AICc. PCs scores were plotted along the phylogeny to assess the evolution of climatic niches. Moreover, we tested the phylogenetic signal (PS) for each PC by calculating Blomberg’s K (K) (Blomberg *et al*., 2003) with 1000 simulations and Pagel’s lambda (λ) (Pagel, 1999) using maximum likelihood. All analyses were performed with R. Additionally, we used raster libraries (Hijmans, 2017) for the extraction of climatic information; vegan, ggplot2, ggfortify, and cluster libraries were used for calculating Z-scores, PCA, and broken stick analyses, while geiger (Harmon *et al*., 2008) and phytools (Revell, 2012) libraries were used for all phylogenetic analyses. Furthermore, we conducted a preliminary PCA with all the layers and determined that only some bioclim layers and Average Potential Evapotranspiration in May (PET5), Average Precipitation in May (Prec5), Average Precipitation in October (Prec10), and Average Maximum Temperature in January (Tmax1) should be retained, as low loadings were produced for the remaining bioclimatic layers. However, in order to compare with other layers, we decided to use the entire bioclim layers from WorldClim and the four additional layers. Nevertheless, results with only bioclim layers were very similar to the analyses reported here.

**Results**

According to the broken stick method, we decided to retain the first three components of the final PCA, which explained 86.6% of the variation in the data (Table S6). PC1 generally showed that the majority of bioclimatic variables had an equivalent amount of variation, and no clear association was evident in 5 of the 23 layers used: Max Temperature of Warmest Month (Bio5), Mean Temperature of Warmest Quarter (Bio10), Precipitation of Driest Month (Bio14), Precipitation Seasonality (Bio15), and Precipitation of Coldest Quarter (Bio19). However, upon interpretation, slightly more association was observed in variables linked to variation in temperature (i.e. Temperature Seasonality (Bio4) and Temperature Annual Range (Bio7) in a positive way and Isothermality (Bio3) in a negative way). Moreover, a negative association also existed with precipitation throughout the year and the wettest months. PC2 exhibited large negative associations with temperature, particularly during the wettest, driest, and warmest months. PC3 showed a clear positive association with Precipitation Seasonality (Bio15) and a negative association with precipitation in driest and coldest months. The 3dPCA plot (Fig. S5) showed that *S. torquatus* species did not generally occupy a defined region of climatic niche space, even with sister or closer species occupying different regions in the climatic space. Furthermore, the evolution of PC1 (Fig. S6) showed that intermediate scores (green color) correspond with ancestral states, and that these states remain unchanged or present little change toward the tips of some branches. Nevertheless, some positive and negative extreme scores (relative to the centroid of the data) arise in the terminal branches and tips across the phylogeny, which coincides with a BM evolution model (Table S6) with strong PS (K = 1.062, *p* = 0.034; λ = 1.873, *p* = 0.033) and variance distributed between clades (λ >1 is possible due to a scaling factor for a correlation [Revell comment in http://blog.phytools.org/2012/03/phylogenetic-signal-with-k-and.html]). This can perhaps be interpreted as few species presenting opposite requirements, and inhabiting habitats with extreme and low variation in temperature; for example, along its distribution, *S. serrifer* inhabits habitats with great variation in temperature (*i.e.* populations in the Guatemala highlands and Yucatan lowlands). In appearance, the evolution of PC2 presented a trend to PC1; however, the BM evolution model is not the best explanation for the evolution of the observed scores of this eigenvector. Despite BM having the lowest Akaike value, the difference with OU model is < 1 (S7.Table) and no evidence of PS (K = 0.889, *p* = 0.614; λ = 7.77 e^-05^, *p* = 1) was observed. In this case, the interpretation is more difficult due to some species apparently possessing very divergent temperature requirements during different months. Only in the clade formed by *S. poinsetti*, *S. sugillatus,* and *S. mucronatus* can we possible observe some relevance, as this species inhabits conifer forests and its reproduction season coincides with driest months. Additionally, PC3 showed that ancestral states were closed to extreme values (blue colors), and the majority of species preserved this character, with some evolving slightly higher values, and only one clade evolving opposite values (*S. serrifer* clade). This behavior is explained by the evolution mode coinciding with a Pagel’s delta (δ) value > 1, which indicates a rapid recent evolution with trait evolution concentrated in the tips—at least in the *S. serrifer* clade. The PS was strong, with variance distributed between clades and a mild correlation with the BM evolution process (K = 0.897, *p* = 0.009; λ = 0.897, *p* = 0.0976).

**Discussion**

According with the cold-climate hypothesis (Shine, 1985) and for the prediction that viviparous species exhibit speciation via climatic niche conservatism, with similar niches being exhibited in allopatric sister species (Lambert & Wiens 2013), we expected high correlation and phylogenetic niche conservatism (PNC) among bioclimatic variables linked with temperate climates or with reproduction season among viviparous lizard species in Mexico and the southern US (fall-winter, Gillette & Méndez de la Cruz, 1993). However, all PCs demonstrated clear evidence of this expectation. PC1 showed some evidence of PNC with PS and a correlation with the BM process of evolution (Münkemüller *et al*. 2015); nevertheless, there was no clear evidence of a link between temperate conditions or with fall-winter (first and last quarter in bioclim variables) conditions with a complex combination of variables that is different for each species—and perhaps only those bioclimatic variables linked with some grade of variation in temperature could be important for this species. Moreover, the PC2 axes do not suggest PNC and did not have a clear evolution mode; therefore, it remains difficult to form a conclusion, though this reflects great heterogeneity in temperature and humidity requirements among members of this species. Additionally, PC3 showed no support for PNC (strong PS but slight correlation with a BM evolution process), with a tendency for rapid recent evolution in some species through Precipitation Seasonality (Bio15) and precipitation in driest and coldest months. In this case, we observe that *S. serrifer* clade sustains species that are more xerophitic prone or have populations in semidesertic areas; for example, *S. serrifer* at its northernmost distribution in the Yucatan peninsula inhabits dry forests, while the other species—with the exception of *S. prezygus*—have populations both in *Pinu*s and *Quercus* forests and in ecotones with xerophytic scrubs (NMM personal records).

However, differences among species are possibly not only linked with adaptations to different realized niches, but with microclime characteristics that are impossible to measure at least this scale. It is likely that *S. torquatus* species could have similar requirements that microclimates could similarly provide for the majority of species. The only way to determine the relative importance of microclimate on the establishment of species of the *S. torquatus* group in different vegetation and climates will be through thermal ecological studies and mechanistic approximations of the fundamental thermal niche. The final assumption is based on certain observations and thermal ecology data pertaining to *S. serrifer*, which occurs in highly contrasting sites (Martínez-Méndez *et al*., 2015).

**References**

Gillette LJ Jr, Méndez-de la Cruz FR. 1993. The reproductive cycle of the viviparous Mexican lizard *Sceloporus torquatus*. *Journal of Herpetology* 27(2): 168-174.

Harmon LJ, Weir JT, Brock CD, Glor RE, Challenger W. 2008. Geiger: investigating evolutionary radiations. Bioinformatics, 24(1): 129-131.

Heibl C, Calenge C. 2015. Phyloclim: Integrating phylogenetics and climatic niche modeling. R package version 0.9.4. *Available at http://cran.r-project.org/ web/packages/phyloclim*.

Hijmans RJ. 2017. Raster: Geographic analysis and modeling with raster data. R package version 2.6-7. *Available at http://cran.r-project.org/package=raster.*

Lambert SM, Wiens JJ. 2013. Test of the cold-climate hypothesis in phrynosomatid lizards. *Evolution* 67(9): 2614-2630. DOI: 10.1111/evo.12130.

Martínez-Méndez N, Mejía O, Méndez-de la Cruz FR. 2015. The past, present and future of a lizard: The phylogeography and extinction risk of *Sceloporus serrifer* (Squamata: Phrynosomatidae) under a global warming scenario. *Zoologischer Anzeiger* 254: 86-98. DOI: 10.1016/j.jcz.2014.12.004.

Münkemüller T, Boucher F, Thuiller W, Lavergne S. 2015. Phylogenetic niche conservatism – common pitfalls and ways forward. *Functional Ecology* 29: 627-639. DOI:10.1111/1365-2435.12388.

Paradis E, Claude J, Strimmer K. 2004. APE: analyses of phylogenetics and evolution in R language. *Bioinformatics* 20(2): 289-90. DOI: 10.1093/bioinformatics/btg412*.*

R Core Team. 2017. R: A language and environment for statistical computing. R Foundation for Statistical Computing, Vienna, Austria. *Available at http://www.R-project.org/.*

Revell LJ. 2012. phytools: an R package for phylogenetic comparative biology (and other things). *Methods in Ecology and Evolution* 3: 217-223. DOI: 10.1111/j.2041-210X.2011.00169.x.

Shine R. 1985. The evolution of viviparity in reptiles: an ecological analysis. In: Gans C, Billet F. eds. *Biology of the Reptilia 15.* New York: John Wiley & Sons, 607-681.

S6. Table. Loadings of Principal Component Analysis of bioclimatic variables.

| Variable | PC1 | PC2 | PC3 |
| --- | --- | --- | --- |
| Annual Mean Temperature(Bio1) | -0.128 | -0.342 | -0.007 |
| Mean Diurnal Range (Bio2) | 0.222 | 0.153 | 0.182 |
| Isothermality (Bio3) | -0.282 | 0.034 | 0.127 |
| Temperature Seasonality (Bio4) | 0.300 | -0.003 | -0.098 |
| Max Temperature of Warmest Month (Bio5) | 0.152 | -0.330 | -0.089 |
| Min Temperature of Coldest Month (Bio6) | -0.215 | -0.275 | -0.084 |
| Temperature Annual Range (Bio7) | 0.300 | 0.070 | 0.028 |
| Mean Temperature of Wettest Quarter (Bio8) | 0.083 | -0.357 | -0.151 |
| Mean Temperature of Driest Quarter (Bio9) | -0.128 | -0.342 | -0.007 |
| Mean Temperature of Warmest Quarter (Bio10) | 0.093 | -0.352 | -0.157 |
| Mean Temperature of Coldest Quarter (Bio11) | -0.211 | -0.284 | -0.034 |
| Annual Precipitation (Bio12) | -0.297 | 0.100 | 0.011 |
| Precipitation of Wettest Month (Bio13) | -0.288 | 0.088 | 0.106 |
| Precipitation of Driest Month (Bio14) | -0.061 | 0.105 | -0.487 |
| Precipitation Seasonality (Bio15) | -0.152 | -0.041 | 0.440 |
| Precipitation of Wettest Quarter (Bio16) | -0.282 | 0.087 | 0.141 |
| Precipitation of Driest Quarter (Bio17) | -0.065 | 0.126 | -0.484 |
| Precipitation of Warmest Quarter (Bio18) | -0.201 | 0.073 | -0.017 |
| Precipitation of Coldest Quarter (Bio19) | -0.042 | 0.129 | -0.319 |
| Average Potential Evapotranspiration in May (Pet5) | 0.191 | -0.243 | 0.066 |
| Average precipitation in May (Prec5) | -0.212 | 0.101 | -0.227 |
| Average precipitation in October (Prec10) | -0.276 | 0.073 | -0.146 |
| Average maximum temperature in January (Tmax1) | -0.219 | -0.264 | 0.041 |
| Proportion of explained variance | 43.2% | 28.1% | 15.3% |

S7. Table. Evolutionary models for niche dimensions.

| PC | Model | lnL | AICc | Parameters |
| --- | --- | --- | --- | --- |
| PC1 | **BM** | **-58.113** | **120.825** | **2** |
|  | EB | -57.973 | 123.210 | 3 |
|  | δ (2.6) | -58.104 | 123.471 | 3 |
|  | OU | -58.113 | 123.488 | 3 |
| PC2 | **BM** | **-55.197** | **114.994** | **2** |
|  | OU | -54.166 | 115.594 | 3 |
|  | δ (2.9) | -54.233 | 115.729 | 3 |
|  | EB | -55.197 | 117.657 | 3 |
| PC3 | **δ (2.9)** | **-45.784** | **96.167** | **2** |
|  | EB | -45.782 | 98.827 | 3 |
|  | BM | -45.783 | 98.830 | 3 |
|  | OU | -45.784 | 98.831 | 3 |


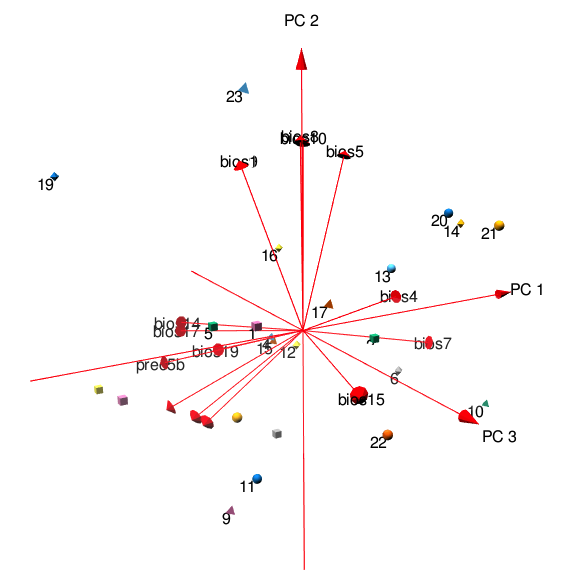


Figure S5. Principal Component Analysis (PCA) of the variables used to represent the ecological niche space of *S.torquatus species.* PCA Lebels: 1*. Sceloporus aureolus*, 2. *S. binocularis*, 3. *S. bulleri*, 4. *S. cyanogenys*, 5. *S. cyanostictus*, 6. *S. dugesii*, 7 *S. insignis*, 8. *S. jarrovii*, 9. *S. lineolateralis*, 10. *S. macdougalli*, 11*. S. melanogaster,* 12. *S. minor*, 13. *S. mucronatus*, 14. *S. oberon*, 15. S. omiltemanus, 16. *S. ornatus caeruleus*, 17. S*. ornatus ornatus*, 18. *S. poinsettii*, 19. *S. prezygus*, 20. *S. serrifer*, 21. *Sceloporus* sp., 22. *S. sugillatus*, 23. *S. torquatus*.


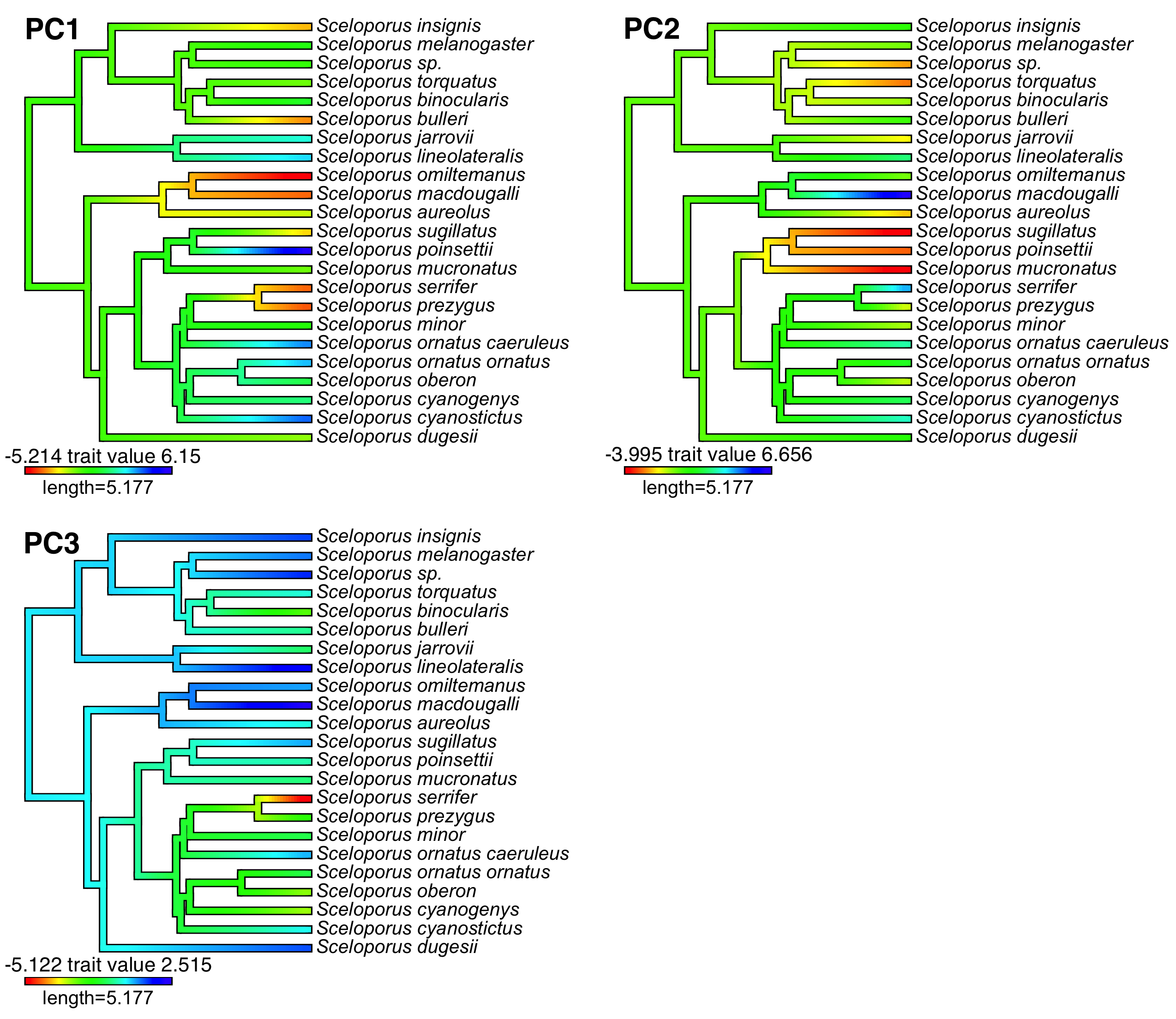


Figure S6. Variation in PC scores of bioclimatic variables along the phylogeny of the *S. torquatus* group.
