# Supplementary material for: Climatic niche evolution in the viviparous Sceloporus torquatus group (Squamata: Phrynosomatidae)
Source: PeerJ. 2019 Jan 9;6:e6192. doi: 10.7717/peerj.6192 (PMC6330044; doi:10.7717/peerj.6192)
Supplement: Supplemental Information 2 [file peerj-07-6192-s002.doc]

| Species | Voucher | GenBank accession number | | | | | | | |
| --- | --- | --- | --- | --- | --- | --- | --- | --- | --- |
|  |  | 12S | 16S | ND4 | ND1 | RAG1 | BDNF | R35 | PNN |
| *Sceloporus aureolus* | IBH 18022 | DQ525884 | DQ525901 | DQ525875 |  |  |  |  |  |
| *Sceloporus binocularis* | MZFC 8033 | AF000827 | AF000867 |  |  |  |  |  |  |
| *Sceloporus bulleri* | IBH 18034 | DQ525887 | DQ525904 | DQ525865 |  |  |  |  |  |
| *Sceloporus cyanogenys* | LSUMZ 48852 | AF15414 | AF15414 | AF154193 |  |  |  |  |  |
| *Sceloporus cyanostictus* | MZFC 7411b | AF000825 | AF000865 | AF154195 |  |  |  |  |  |
| *Sceloporus dugesii* | UTA-R 23955 | AF154170 | AF000877 | AF154190 | GQ464474 | GQ464719 | GQ464439 | GQ464663 | GQ464607 |
| *Sceloporus insignis* | No inf. | AF000806 | AF000846 |  |  |  |  |  |  |
| *Sceloporus jarrovii* | LSUMZ 48786 | AF154163 | AF000881 | GQ464765 | GQ464485 | GQ464720 | GQ464440 | GQ464664 | GQ464608 |
| *Sceloporus lineolateralis* | MZFC 6650 | AF000807 | AF000847 | AF154211 | GQ464487 | GQ464721 | GQ464441 | GQ464665 | GQ464609 |
| *Sceloporus macdougalli* | MZFC 7017 | AF000809 | AF000849 | GQ895844 | GQ464488 | GQ464722 | GQ464442 | GQ464666 | GQ464610 |
| *Sceloporus melanogaster* | UTA-R 24016 | GQ464569 | AF000890 | GQ464793 | GQ464513 | GQ464726 | GQ464446 | GQ464670 | GQ464614 |
| *Sceloporus minor* | MZFC 10736 | AF154138 |  | AF154198 |  |  |  |  |  |
| *Sceloporus mucronatus* | IBH 18008 | DQ525885 | DQ525902 | DQ525864 |  |  |  |  |  |
| *Sceloporus oberon* | MZFC 8032 | AF000826 | AF000866 | AF154212 |  |  |  |  |  |
| *Sceloporus omiltemanus* | UTA-R 24004 | AF440094 | AF440094 | GQ464775 | GQ464495 | GQ464723 | GQ464443 | GQ464667 | GQ464611 |
| *Sceloporus ornatus ornatus* | IBH 18041 | DQ525879 | DQ525896 | DQ525862 |  |  |  |  |  |
| *Sceloporus o. caeruleus* | JAM 652 | AF000814 | AF000854 | AF154240 | GQ464500 | KU768399 | KU765654 | KU768498 | GQ464612 |
| *Sceloporus poinsettii* | LSUMZ 48847 | AF154176 | AF000883 | AF154241 | GQ464504 | GQ464725 | GQ464445 | GQ464669 | GQ464613 |
| *Sceloporus prezygus* | IBH 18027 | DQ525880 | DQ525897 | DQ525870 |  |  |  |  |  |
| *Sceloporus torquatus* | IBH 18050 | DQ525892 | DQ525909 | DQ525863 |  |  |  |  |  |
| *Sceloporus serrifer* | IBH 18020 | DQ525894 | DQ525911 | DQ525876 |  |  |  |  |  |
| *Sceloporus sp.* | MX14-4 | EF608018 | EF608026 | EF608021 |  |  |  |  |  |
| *Sceloporus sugillatus* | CM 147623a | AF154187 |  | AF154242 |  |  |  |  |  |
| *Sceloporus grammicus* | UTA-R 23970 | L40457 | L41464 | AF154188 | GQ464479 | GQ464704 | GQ464424 | GQ464648 | GQ464592 |
| *Sceloporus heterolepis* | IBH 18138 (12S,16S)/MZFC 8017 | DQ525890 | DQ525907 | GQ464761 | GQ464481 | GQ464705 | GQ464425 | GQ464649 | GQ464593 |
| *Sceloporus palaciosi* | JJW 401 | GQ464557 |  | GQ464781 | GQ464501 | GQ464706 | GQ464426 | GQ464650 | GQ464594 |
