# Supplementary material for: Climatic niche evolution in the viviparous Sceloporus torquatus group (Squamata: Phrynosomatidae)
Source: PeerJ. 2019 Jan 9;6:e6192. doi: 10.7717/peerj.6192 (PMC6330044; doi:10.7717/peerj.6192)
Supplement: Supplemental Information 3 [file peerj-07-6192-s003.pdf]

Species	Collection code	Catalog number	Country	State or province	Latitude	Longitude
S. aureolus	CAS	82014	México	Oaxaca	17.3613	-97.1747
S. aureolus	CAS	103433	México	Oaxaca	17.3300	-96.4600
S. aureolus	CAS	169576	México	Oaxaca	16.1300	-97.0300
S. aureolus	CAS	114070	México	Oaxaca	17.7300	-97.3500
S. aureolus	CAS	169593	México	Oaxaca	16.4500	-97.1700
S. aureolus	CUMV	9993	México	Oaxaca	17.1520	-97.6230
S. aureolus	ENCB	5463	México	Oaxaca	17.9260	-97.4140
S. aureolus	ENCB	10337	México	Oaxaca	17.3560	-98.0320
S. aureolus	ENCB	5460	México	Oaxaca	17.8070	-97.4710
S. aureolus	ENCB	5464	México	Oaxaca	17.8070	-97.4840
S. aureolus	ENCB	2091	México	Oaxaca	17.1410	-96.0490
S. aureolus	ENCB	6630	México	Oaxaca	16.6940	-96.9780
S. aureolus	MZFC	11164	México	Oaxaca	17.3400	-97.2500
S. aureolus	MZFC	6042	México	Oaxaca	17.3100	-97.1700
S. aureolus	MZFC	15666	México	Oaxaca	17.3400	-97.2300
S. aureolus	MZFC	15657	México	Oaxaca	17.3300	-97.2200
S. aureolus	MZFC	6044	México	Oaxaca	17.3400	-97.2700
S. aureolus	MZFC	16088	México	Oaxaca	16.4000	-96.2300
S. aureolus	MZFC	16122	México	Oaxaca	16.5700	-96.1200
S. aureolus	MZFC	13538	México	Oaxaca	16.5900	-97.3800
S. aureolus	MZFC	6493	México	Oaxaca	17.9330	-96.1660
S. aureolus	MZFC	6044	México	Oaxaca	17.5830	-97.4500
S. aureolus	MZFC	16052	México	Oaxaca	17.4000	-96.2000
S. aureolus	MZFC	3195	México	Oaxaca	17.1900	-96.2800
S. aureolus	MZFC	7443	México	Oaxaca	17.2800	-96.5700
S. aureolus	MZFC	6041	México	Oaxaca	17.5330	-97.3000
S. aureolus	MZFC	7450	México	Oaxaca	17.1300	-97.4300
S. aureolus	MZFC	3193	México	Oaxaca	17.1600	-96.3900
S. aureolus	MZFC	6493	México	Oaxaca	17.5500	-96.1000
S. aureolus	MZFC	6623	México	Oaxaca	17.1400	-96.3300
S. aureolus	MZFC	16091	México	Oaxaca	17.1400	-96.3400
S. aureolus	MZFC	16234	México	Oaxaca	16.2800	-96.5900
S. aureolus	FMNH	112245	México	Oaxaca	17.1510	-97.6240
S. aureolus	CNAR	7059	México	Oaxaca	17.2500	-97.6900
S. aureolus	CNAR	7415	México	Oaxaca	17.2200	-97.7000
S. aureolus	CNAR	7471	México	Oaxaca	17.2700	-97.6800
S. aureolus	CNAR	7159	México	Oaxaca	17.3200	-96.4600
S. aureolus	CNAR	11248	México	Oaxaca	17.6008	-97.6008
S. aureolus	CNAR	AR5140	México	Oaxaca	17.6008	-97.6000
S. aureolus	CNAR	AR5131	México	Oaxaca	17.2240	-97.0360
S. aureolus	CNAR	7160	México	Oaxaca	17.2300	-97.0000
S. aureolus	CNAR	61986	México	Oaxaca	17.3500	-97.0500
S. aureolus	CNAR	6228	México	Oaxaca	17.2600	-96.5400
S. aureolus	CNAR	11245	México	Oaxaca	17.8522	-97.5703
S. aureolus	CNAR	11246	México	Oaxaca	17.8361	-97.5500
S. aureolus	CNAR	AR5139	México	Oaxaca	17.8360	-97.5500
S. aureolus	CNAR	3359	México	Oaxaca	17.6500	-97.4200
S. aureolus	CNAR	AR5130	México	Oaxaca	17.6500	-97.4200
S. aureolus	CNAR	2891	México	Oaxaca	17.5300	-97.3500
S. aureolus	CNAR	6755	México	Oaxaca	17.6900	-97.5700
S. aureolus	CNAR	390	México	Oaxaca	17.6700	-97.5700
S. aureolus	MCZ	R-136482	México	Oaxaca	16.9900	-97.6400
S. aureolus	MCZ	R-122066	México	Oaxaca	17.8070	-97.4840
S. aureolus	MCZ	R-42728	México	Oaxaca	17.8660	-97.1940
S. aureolus	MCZ	46932	México	Oaxaca	17.1330	-96.0160
S. aureolus	MCZ	R-121122	México	Oaxaca	16.6940	-96.9780
S. aureolus	MVZ	57226	México	Oaxaca	17.3261	-96.4500
S. aureolus	MVZ	164292	México	Oaxaca	17.3300	-96.4870
S. aureolus	MVZ	104060	México	Oaxaca	16.3080	-96.9770
S. aureolus	MVZ	164779	México	Oaxaca	17.4000	-96.5140
S. aureolus	ND	61092	México	Oaxaca	17.5500	-97.3740
S. aureolus	ND	R-42729	México	Oaxaca	16.3083	-96.9777
S. aureolus	ND	18547	México	Oaxaca	17.2330	-97.0000
S. aureolus	ND	112209	México	Oaxaca	16.0820	-96.3950
S. aureolus	ND	19651	México	Oaxaca	16.7000	-97.0160
S. aureolus	ND	61090	México	Oaxaca	17.0850	-97.7200
S. aureolus	ND	6075	México	Oaxaca	17.9750	-97.3160
S. aureolus	ND	11776	México	Oaxaca	16.4830	-96.9840
S. aureolus	LACM	96202	México	Oaxaca	17.6200	-97.2000
S. aureolus	LACM	128492	México	Oaxaca	17.5200	-97.4500
S. aureolus	LACM	96201	México	Oaxaca	17.6800	-97.2000
S. aureolus	USNM	47582	México	Oaxaca	17.3330	-96.4830
S. aureolus	USNM	112220	México	Oaxaca	16.4700	-96.9780
S. aureolus	ROM	16671	México	Oaxaca	17.3100	-96.5500

S. aureolus	TCWC	22051	México	Oaxaca	17.3560	-98.0320
S. aureolus	UAZ	UAZ 29929	México	Oaxaca	17.6200	-96.9200
S. aureolus	UAZ	UAZ 29478	México	Oaxaca	17.6100	-97.3700
S. aureolus	UCM	61092	México	Oaxaca	17.1600	-97.8300
S. aureolus	UCM	61085	México	Oaxaca	17.7370	-97.1190
S. aureolus	UCM	61086	México	Oaxaca	17.7520	-97.1360
S. aureolus	UCM	61110	México	Oaxaca	17.8070	-97.4710
S. aureolus	UCM	61084	México	Oaxaca	17.6860	-97.3380
S. aureolus	UCM	61088	México	Oaxaca	17.7520	-97.0840
S. aureolus	UCM	61090	México	Oaxaca	17.7030	-97.1520
S. aureolus	UCM	61082	México	Oaxaca	17.5860	-97.0040
S. aureolus	UCM	61091	México	Oaxaca	17.8690	-97.3530
S. aureolus	IUMNH	60891	México	Oaxaca	17.3200	-96.4600
S. aureolus	IUMNH	21364	México	Oaxaca	16.9560	-96.2140
S. aureolus	IUMNH	8759	México	Oaxaca	16.1330	-96.5000
S. aureolus	IUMNH	60471	México	Oaxaca	15.8000	-96.4600
S. aureolus	IUMNH	21331	México	Oaxaca	16.2100	-96.4700
S. aureolus	ND	489	México	Oaxaca	17.9580	-97.3300
S. aureolus	UTA	11774	México	Oaxaca	18.1580	-97.0000
S. binocularis	CAS	147381	México	Nuevo León	24.7700	-99.9700
S. binocularis	CM	59722	México	Nuevo León	23.7430	-100.3800
S. binocularis	UANL	197	México	Nuevo León	24.8270	-100.0760
S. binocularis	UANL	3857	México	Nuevo León	24.8760	-100.2200
S. binocularis	UANL	1055	México	Nuevo León	23.9080	-99.7970
S. binocularis	UANL	594	México	Nuevo León	24.6790	-99.8450
S. binocularis	UANL	2508	México	Nuevo León	25.1890	-99.8270
S. binocularis	UANL	2799	México	Nuevo León	25.2170	-100.1180
S. binocularis	UANL	3821	México	Nuevo León	25.2170	-100.1340
S. binocularis	UANL	3824	México	Nuevo León	25.2000	-100.1170
S. binocularis	UANL	2199	México	Nuevo León	25.5780	-100.3060
S. binocularis	UANL	1910	México	Nuevo León	25.1190	-100.2000
S. binocularis	UANL	3832	México	Nuevo León	25.2170	-100.1510
S. binocularis	UANL	2711	México	Nuevo León	25.3720	-100.2210
S. binocularis	UANL	2714	México	Nuevo León	25.2500	-100.1610
S. binocularis	UANL	2730	México	Nuevo León	25.3000	-100.1410
S. binocularis	UANL	2783	México	Nuevo León	25.3720	-100.2240
S. binocularis	UANL	2803	México	Nuevo León	25.3420	-100.1820
S. binocularis	UANL	2808	México	Nuevo León	25.3130	-100.2090
S. binocularis	MZFC	747	México	Nuevo León	25.0330	-100.3000
S. binocularis	FMNH	30776	México	Nuevo León	24.8160	-100.0830
S. binocularis	FMNH	30769	México	Nuevo León	25.1830	-99.8500
S. binocularis	FMNH	36902	México	Nuevo León	25.4160	-100.1500
S. binocularis	FMNH	36904	México	Nuevo León	25.4160	-100.1330
S. binocularis	UCM	48392	México	Nuevo León	24.1040	-99.9240
S. bulleri	CAS	3711	México	Jalisco	20.0340	-103.4150
S. bulleri	CAS	169640	México	Jalisco	19.5700	-103.5200
S. bulleri	CAS	159352	México	Sinaloa	23.4300	-105.8300
S. bulleri	CAS	160213	México	Sinaloa	23.5900	-105.8400
S. bulleri	ENCB	14952	México	Jalisco	20.0340	-103.4150
S. bulleri	ENCB	14953	México	Jalisco	19.9320	-102.9140
S. bulleri	MZFC	6774	México	Jalisco	19.4670	-103.9500
S. bulleri	MZFC	6786	México	Sinaloa	23.5330	-105.9000
S. bulleri	CNAR	5883	México	Jalisco	20.3800	-104.5500
S. bulleri	CNAR	5884	México	Jalisco	20.4800	-104.7800
S. bulleri	CNAR	5885	México	Jalisco	20.3800	-104.9200
S. bulleri	CNAR	5886	México	Jalisco	20.4200	-104.9800
S. bulleri	MSUM	HE.4159	México	Jalisco	19.6600	-104.4000
S. bulleri	MVZ	44694	México	Durango	23.6400	-105.8100
S. bulleri	LACM	6672	México	Colima	19.2900	-104.1000
S. bulleri	LACM	61988	México	Jalisco	19.7660	-104.3660
S. bulleri	LACM	50959	México	Jalisco	19.6000	-104.3900
S. bulleri	LACM	50957	México	Sinaloa	23.3100	-106.0000
S. bulleri	LACM	95584	México	Sinaloa	23.5300	-106.0000
S. bulleri	LACM	116347	México	Sinaloa	23.4400	-105.8400
S. bulleri	LACM	50963	México	Sinaloa	23.3900	-105.8100
S. bulleri	USNM	8701	México	Jalisco	20.3660	-102.9660
S. bulleri	SDNHM	48186	México	Jalisco	20.4820	-105.2930
S. bulleri	SDNHM	48183	México	Jalisco	20.5300	-105.3000
S. bulleri	TCWC	54542	México	Sinaloa	23.4500	-105.8500
S. bulleri	UCM	50017-018	México	Sinaloa	23.5330	-105.8330
S. bulleri	IUMNH	46581-602	México	Jalisco	20.5330	-104.7830
S. bulleri	IUMNH	41621-622	México	Sinaloa	23.3000	-105.8500
S. bulleri	UMMZ	101957-958	México	Jalisco	19.7500	-103.4660
S. bulleri	UTEP	7029	México	Durango	23.4000	-105.6200
S. caeruleus	FMNH	115763	México	Coahuila	25.5000	-103.2830

S. caeruleus	KU	37916	México	Coahuila	27.0330	-102.4500
S. caeruleus	KU	33615-617	México	Coahuila	27.0660	-102.4160
S. caeruleus	KU	37916-918	México	Coahuila	27.0330	-102.4500
S. caeruleus	KU	33615	México	Coahuila	27.3100	-102.4100
S. caeruleus	KU	33865	México	Coahuila	25.5000	-103.2830
S. caeruleus	USNM	3776	México	Coahuila	25.7330	-102.4500
S. caeruleus	USNM	3793	México	Coahuila	25.7000	-103.0000
S. caeruleus	USNM	50910	México	Coahuila	25.6330	-102.6000
S. caeruleus	USNM	3752	México	Coahuila	25.5830	-102.7160
S. caeruleus	TCWC	49562	México	Coahuila	25.6160	-102.8830
S. caeruleus	IUMNH	43342-344	México	Coahuila	25.6660	-102.5000
S. caeruleus	IUMNH	43348-353	México	Coahuila	25.6160	-102.8830
S. caeruleus	UTA	R2020	México	Coahuila	25.3200	-103.2500
S. cyanogenys	AMNH	110258	México	Tamaulipas	23.9380	-99.0180
S. cyanogenys	AMNH	74304	México	Tamaulipas	23.1300	-99.1400
S. cyanogenys	CAS	141825	México	Tamaulipas	24.1500	-98.5600
S. cyanogenys	EAL	4131	México	Nuevo León	26.3140	-99.5380
S. cyanogenys	EAL	747	México	Tamaulipas	24.1080	-98.8360
S. cyanogenys	UANL	320	México	Nuevo León	25.7056	-99.3500
S. cyanogenys	UANL	1827	México	Nuevo León	23.7430	-100.3800
S. cyanogenys	UANL	326	México	Nuevo León	25.8090	-100.5930
S. cyanogenys	UANL	48340	México	Nuevo León	25.7830	-99.1830
S. cyanogenys	UANL	1180	México	Nuevo León	26.2210	-99.4820
S. cyanogenys	UANL	1161	México	Nuevo León	25.6300	-100.2320
S. cyanogenys	UANL	2063	México	Nuevo León	25.6940	-100.2190
S. cyanogenys	UANL	32217	México	Nuevo León	25.6460	-100.0920
S. cyanogenys	UANL	17364	México	Nuevo León	26.0640	-99.4000
S. cyanogenys	UANL	3939	México	Nuevo León	26.5460	-99.4250
S. cyanogenys	UANL	1145	México	Nuevo León	25.7520	-100.0180
S. cyanogenys	UANL	1205	México	Nuevo León	25.6970	-99.6260
S. cyanogenys	UANL	51	México	Nuevo León	26.1910	-100.4800
S. cyanogenys	UANL	4149	México	Nuevo León	25.9640	-100.2950
S. cyanogenys	UANL	469	México	Nuevo León	25.7350	-100.3270
S. cyanogenys	UANL	472	México	Nuevo León	25.7360	-100.3050
S. cyanogenys	UANL	2064	México	Nuevo León	25.6760	-100.4620
S. cyanogenys	UANL	4150	México	Tamaulipas	22.9560	-97.9070
S. cyanogenys	UANL	4819	México	Tamaulipas	23.6470	-99.6390
S. cyanogenys	UANL	4324	México	Tamaulipas	24.6230	-99.0270
S. cyanogenys	MZFC	15877	México	Nuevo León	25.6000	-99.9300
S. cyanogenys	FMNH	48340	México	Nuevo León	25.7820	-100.1860
S. cyanogenys	FMNH	112225	México	Nuevo León	25.9560	-100.1680
S. cyanogenys	FMNH	98416	México	Nuevo León	25.6640	-100.3110
S. cyanogenys	FMNH	123825	México	Nuevo León	26.5000	-99.5230
S. cyanogenys	FMNH	1775	México	Nuevo León	26.4470	-100.1560
S. cyanogenys	FMNH	1829	México	Nuevo León	26.5350	-100.2440
S. cyanogenys	FMNH	32111	México	Nuevo León	26.4390	-100.1470
S. cyanogenys	FMNH	32220	México	Nuevo León	26.5130	-100.1770
S. cyanogenys	FMNH	32222	México	Nuevo León	26.5010	-100.1730
S. cyanogenys	FMNH	2447	México	Nuevo León	25.8700	-100.2620
S. cyanogenys	FMNH	32214	México	Nuevo León	25.9450	-100.2690
S. cyanogenys	FMNH	98417	México	Nuevo León	25.6500	-100.4580
S. cyanogenys	CNAR	4700	México	Nuevo León	25.4700	-100.6300
S. cyanogenys	CNAR	4742	México	San Luis Potosí	23.6900	-100.8900
S. cyanogenys	CNAR	4743	México	San Luis Potosí	22.5600	-100.3600
S. cyanogenys	CNAR	4744	México	San Luis Potosí	22.6200	-100.4400
S. cyanogenys	CNAR	4592	México	Tamaulipas	24.7500	-98.2500
S. cyanogenys	CNAR	4593	México	Tamaulipas	23.6400	-98.1800
S. cyanogenys	CNAR	4594	México	Tamaulipas	22.9200	-99.6300
S. cyanogenys	KU	62082	México	Nuevo León	24.5150	-99.9950
S. cyanogenys	KU	94504	México	Nuevo León	25.6430	-100.3520
S. cyanogenys	KU	23231	México	Nuevo León	26.4360	-100.1520
S. cyanogenys	KU	3920	México	Tamaulipas	24.7050	-99.0770
S. cyanogenys	KU	68099	México	Tamaulipas	24.6490	-99.0510
S. cyanogenys	KU	68100	México	Tamaulipas	24.5470	-99.0820
S. cyanogenys	KU	33998	México	Tamaulipas	23.7630	-98.2110
S. cyanogenys	MVZ	36772	México	Nuevo León	25.7030	-99.2620
S. cyanogenys	MVZ	36772	México	Nuevo León	25.7056	-99.3500
S. cyanogenys	MVZ	32198	México	Nuevo León	24.8760	-100.2200
S. cyanogenys	MVZ	129293	México	Nuevo León	24.5680	-100.0230
S. cyanogenys	MVZ	186496	México	Nuevo León	24.8683	-100.2310
S. cyanogenys	MVZ	186498	México	Nuevo León	24.5732	-100.0090
S. cyanogenys	MVZ	186501	México	Nuevo León	24.5157	-99.9950
S. cyanogenys	MVZ	38713	México	Nuevo León	26.5520	-100.4730
S. cyanogenys	MVZ	12701	México	Tamaulipas	27.4452	-99.5490
S. cyanogenys	MVZ	12701	México	Tamaulipas	27.4450	-99.5490

S. cyanogenys	MVZ	79121	México	Tamaulipas	27.4860	-99.5080
S. cyanogenys	LACM	17505	México	Tamaulipas	23.1000	-99.1900
S. cyanogenys	LACM	140633	US	Texas	29.4900	-101.0360
S. cyanogenys	USNM	505730	México	Tamaulipas	26.3270	-99.2600
S. cyanogenys	USNM	505732	México	Tamaulipas	26.4280	-99.1470
S. cyanogenys	USNM	15877	México	Tamaulipas	25.5450	-98.3750
S. cyanogenys	TCWC	58057	México	Nuevo León	24.5920	-99.9950
S. cyanogenys	TCWC	57324	México	Tamaulipas	23.0750	-98.4250
S. cyanogenys	TCWC	52955	México	Tamaulipas	23.6410	-99.6330
S. cyanogenys	TCWC	52956	México	Tamaulipas	23.6580	-99.8640
S. cyanogenys	TCWC	52958	México	Tamaulipas	23.6610	-99.6550
S. cyanogenys	TCWC	52959	México	Tamaulipas	23.6780	-99.6730
S. cyanogenys	TCWC	6969	México	Tamaulipas	24.0800	-98.7520
S. cyanogenys	TCWC	55318	México	Tamaulipas	24.6200	-98.9110
S. cyanogenys	TCWC	58045	México	Tamaulipas	24.6090	-98.9410
S. cyanogenys	TCWC	58051	México	Tamaulipas	24.6110	-98.9160
S. cyanogenys	TCWC	58087	México	Tamaulipas	24.6230	-99.0310
S. cyanogenys	TCWC	58090	México	Tamaulipas	24.6230	-99.0370
S. cyanogenys	TCWC	58092	México	Tamaulipas	24.6230	-99.0410
S. cyanogenys	TCWC	58093	México	Tamaulipas	24.6230	-99.0400
S. cyanogenys	TCWC	58094	México	Tamaulipas	24.6230	-99.0480
S. cyanogenys	TCWC	58096	México	Tamaulipas	24.6230	-99.0450
S. cyanogenys	TCWC	58099	México	Tamaulipas	24.6210	-98.9120
S. cyanogenys	TCWC	58100	México	Tamaulipas	24.6230	-99.0440
S. cyanogenys	TCWC	58102	México	Tamaulipas	24.6230	-99.0380
S. cyanogenys	TCWC	58128	México	Tamaulipas	24.4820	-98.9420
S. cyanogenys	TCWC	58129	México	Tamaulipas	24.4660	-98.8880
S. cyanogenys	TCWC	62083	México	Tamaulipas	24.6230	-99.0330
S. cyanogenys	TCWC	62161	México	Tamaulipas	24.6130	-98.9150
S. cyanogenys	TCWC	62162	México	Tamaulipas	24.6150	-98.9260
S. cyanogenys	TCWC	62163	México	Tamaulipas	24.6160	-98.9240
S. cyanogenys	TCWC	1545	México	Tamaulipas	23.4740	-99.1440
S. cyanogenys	TCWC	35232	México	Tamaulipas	23.6300	-99.0500
S. cyanogenys	TCWC	57031	México	Tamaulipas	23.1180	-98.7420
S. cyanogenys	UCM	48334	México	Nuevo León	25.6970	-99.4280
S. cyanogenys	UCM	48911	México	Nuevo León	25.8000	-100.6500
S. cyanogenys	UCM	38987-988	México	Nuevo León	25.8000	-100.6000
S. cyanogenys	UCM	47357-362	México	Nuevo León	25.7830	-100.5830
S. cyanogenys	UCM	48340	México	Nuevo León	25.7940	-99.1800
S. cyanogenys	UCM	48334-335	México	Nuevo León	25.6330	-99.5830
S. cyanogenys	UCM	48165	México	Tamaulipas	23.0500	-99.1660
S. cyanogenys	UCM	61132	México	Tamaulipas	23.0360	-99.1900
S. cyanogenys	UMMZ	61596	México	Tamaulipas	24.0660	-98.2060
S. cyanogenys	UMMZ	4149	México	Tamaulipas	22.7550	-98.0310
S. cyanogenys	UMMZ	101544	México	Tamaulipas	23.1370	-98.0730
S. cyanogenys	UMMZ	110749	México	Tamaulipas	23.0010	-99.1530
S. cyanogenys	UMMZ	61595	México	Tamaulipas	23.9690	-98.8070
S. cyanogenys	UMMZ	90612	México	Tamaulipas	23.9610	-98.8820
S. cyanogenys	UTEP	7874	México	Tamaulipas	23.1900	-98.4250
S. cyanogenys	UTEP	7882	México	Tamaulipas	23.0390	-99.7210
S. cyanostictus	ND	20937	México	Coahuila	25.7000	-103.1170
S. cyanostictus	MCZ	R-4557	México	Coahuila	26.9000	-101.4160
S. cyanostictus	SNOMNH	40858	México	Coahuila	26.3556	-101.3583
S. cyanostictus	USNM	167353	México	Coahuila	26.3550	-101.3580
S. cyanostictus	UTEP	18621	México	Coahuila	25.1800	-102.9000
S. cyanostictus	MZFC	10684	México	Coahuila	26.1500	-102.7500
S. cyanostictus	MZFC	6837	México	Coahuila	25.7000	-103.1160
S. cyanostictus	MCZ	4557	México	Coahuila	26.9000	-101.4160
S. cyanostictus	ND	S/N / 3851	México	Coahuila	26.9660	-100.7830
S. cyanostictus	USNM	S/N / 3475	México	Coahuila	26.4160	-101.3500
S. cyanostictus	ND	20937	México	Coahuila	25.7000	-103.1170
S. cyanostictus	MCZ	R-4557	México	Coahuila	26.9000	-101.4160
S. cyanostictus	SNOMNH	40858	México	Coahuila	26.3556	-101.3583
S. cyanostictus	USNM	167353	México	Coahuila	26.3550	-101.3580
S. cyanostictus	UTEP	18621	México	Coahuila	25.1800	-102.9000
S. cyanostictus	MZFC	10684	México	Coahuila	26.1500	-102.7500
S. cyanostictus	MZFC	6837	México	Coahuila	25.7000	-103.1160
S. cyanostictus	MCZ	4557	México	Coahuila	26.9000	-101.4160
S. cyanostictus	ND	S/N / 3851	México	Coahuila	26.9660	-100.7830
S. cyanostictus	USNM	S/N / 3475	México	Coahuila	26.4160	-101.3500
S. dugesii	CAS	100222	México	Jalisco	19.9000	-104.3300
S. dugesii	CAS	97252	México	Michoacán	20.1600	-103.0200
S. dugesii	FMNH	33460-467	México	Jalisco	20.9660	-104.0500
S. dugesii	FMNH	999	México	Jalisco	20.3500	-102.7660
S. dugesii	CNAR	5877	México	Jalisco	20.3800	-104.5500

S. dugesii	CNAR	7209	México	Jalisco	19.7100	-103.4600
S. dugesii	CNAR	AR4328	México	Jalisco	19.6500	-103.7000
S. dugesii	CNAR	7149	México	Jalisco	20.3700	-103.2100
S. dugesii	CNAR	2751	México	Jalisco	20.2900	-103.2900
S. dugesii	CNAR	2803	México	Jalisco	20.1700	-102.9600
S. dugesii	CNAR	4257	México	Jalisco	20.2900	-103.1900
S. dugesii	CNAR	6094	México	Jalisco	19.8700	-103.0500
S. dugesii	CNAR	2750	México	Jalisco	20.1100	-103.2500
S. dugesii	CNAR	7568	México	Michoacán	20.1100	-102.8400
S. dugesii	CNAR	AR3392	México	Michoacán	20.0500	-102.9000
S. dugesii	KU	29673-683	México	Jalisco	19.9500	-103.0000
S. dugesii	KU	29268-271	México	Jalisco	20.4660	-103.4830
S. dugesii	KU	38185	México	Michoacán	19.9660	-102.7000
S. dugesii	KU	29153	México	Michoacán	20.0000	-102.8830
S. dugesii	KU	37734	México	Nayarit	20.9500	-104.3660
S. dugesii	MCZ	R-131716	México	Jalisco	20.0900	-104.2000
S. dugesii	MVZ	56286	México	Jalisco	20.8030	-104.2260
S. dugesii	MVZ	72181	México	Jalisco	20.4380	-103.5340
S. dugesii	MVZ	72192	México	Jalisco	20.3850	-103.3460
S. dugesii	MVZ	71248	México	Michoacán	20.0230	-102.5880
S. dugesii	MVZ	56274	México	Nayarit	20.7660	-104.3250
S. dugesii	MVZ	56274	México	Nayarit	20.7670	-104.3257
S. dugesii	ND	S/N / 8107	México	Jalisco	20.2160	-103.3660
S. dugesii	ND	S/N / 8416	México	Jalisco	20.4330	-103.5160
S. dugesii	ND	S/N / 8103	México	Jalisco	20.4330	-103.7160
S. dugesii	ND	S/N / 12732	México	Michoacán	20.0000	-103.0160
S. dugesii	LACM	37609	México	Jalisco	20.3800	-103.5900
S. dugesii	LACM	37610	México	Jalisco	20.4200	-103.6200
S. dugesii	LACM	65201	México	Jalisco	20.2800	-103.1800
S. dugesii	LACM	25773	México	Jalisco	20.0900	-104.2000
S. dugesii	LACM	136871	México	Michoacán	20.1700	-102.6100
S. dugesii	USNM	S/N / 7937	México	Jalisco	20.7660	-104.0830
S. dugesii	USNM	S/N / 12123 / A014	México	Michoacán	20.1500	-102.7160
S. dugesii	USNM	S/N / 8168	México	Nayarit	21.1160	-104.4330
S. dugesii	SDNHM	48980	México	Michoacán	20.0239	-102.5884
S. dugesii	SDNHM	48980	México	Michoacán	20.0300	-102.5700
S. dugesii	TCWC	35234	México	Michoacán	20.1500	-102.7160
S. dugesii	UCM	8704-813	México	Jalisco	20.3500	-103.5160
S. dugesii	UCM	16727	México	Michoacán	20.0000	-103.0160
S. dugesii	IUMNH	6483-496	México	Jalisco	20.5660	-104.0000
S. dugesii	IUMNH	20884	México	Jalisco	19.7660	-104.3000
S. dugesii	IUMNH	20873	México	Jalisco	19.6830	-104.3500
S. dugesii	IUMNH	20857	México	Jalisco	20.1830	-102.6830
S. dugesii	IUMNH	20877-879	México	Jalisco	20.9000	-103.9660
S. dugesii	IUMNH	47924-933	México	Jalisco	20.5660	-103.8500
S. dugesii	IUMNH	20871	México	Michoacán	20.1160	-102.8500
S. dugesii	IUMNH	6413-469	México	Nayarit	21.0330	-104.3330
S. dugesii	UMMZ	119090	México	Michoacán	19.9660	-102.7000
S. dugesii	UTEP	7271	México	Jalisco	19.9400	-103.6800
S. grammicus	CAS	169748	México	Durango	23.9100	-105.3400
S. grammicus	CAS	114924	México	Durango	23.7400	-105.5600
S. grammicus	CAS	142598	México	Oaxaca	17.4833	-96.4500
S. grammicus	CAS	180832	México	Oaxaca	17.6000	-96.4833
S. grammicus	CAS	87232	México	Oaxaca	17.8670	-97.1920
S. grammicus	CAS	156554	México	Puebla	18.9200	-97.2500
S. grammicus	CAS	165242	México	Tamaulipas	23.6000	-99.7100
S. grammicus	CAS	165247	México	Tamaulipas	23.5720	-99.7220
S. grammicus	CAS	135687	México	Veracruz	19.5808	-96.9928
S. grammicus	CAS	165271	México	Zacatecas	21.6800	-103.1600
S. grammicus	CIB-UAEH	CIB-UAEH-949	México	Aguascalientes	22.2060	-102.6250
S. grammicus	CIB-UAEH	CIB-UAEH-582	México	México	19.6980	-98.5780
S. grammicus	CIB-UAEH	CIB-UAEH-660	México	México	19.6640	-98.5890
S. grammicus	CIB-UAEH	CIB-UAEH-668	México	México	19.6820	-98.5760
S. grammicus	CIB-UAEH	CIB-UAEH-363	México	Hidalgo	20.2960	-98.9770
S. grammicus	CIB-UAEH	CIB-UAEH-168	México	Hidalgo	19.7000	-98.4500
S. grammicus	CIB-UAEH	CIB-UAEH-351	México	Hidalgo	20.2940	-98.6790
S. grammicus	CIB-UAEH	CIB-UAEH-367	México	Hidalgo	20.7490	-98.8100
S. grammicus	CIB-UAEH	CIB-UAEH-89	México	Hidalgo	20.1090	-98.6020
S. grammicus	CIB-UAEH	CIB-UAEH-303	México	Hidalgo	20.2820	-98.3470
S. grammicus	CIB-UAEH	CIB-UAEH-253	México	Hidalgo	20.0950	-98.7310
S. grammicus	CIB-UAEH	CIB-UAEH-118	México	Hidalgo	20.0990	-98.7490
S. grammicus	CIB-UAEH	CIB-UAEH-424	México	Hidalgo	19.9010	-98.4780
S. grammicus	CIB-UAEH	CIB-UAEH-659	México	Hidalgo	19.6940	-98.5630
S. grammicus	CIB-UAEH	CIB-UAEH-364	México	Hidalgo	20.0870	-98.3790
S. grammicus	CIB-UAEH	CIB-UAEH-676	México	Hidalgo	20.1310	-98.5270

S. grammicus	CIB-UAEH	CIB-UAEH-356	México	Hidalgo	20.6490	-98.6400
S. grammicus	CIB-UAEH	CIB-UAEH-1331	México	Hidalgo	20.7910	-99.3890
S. grammicus	CIB-UAEH	CIB-UAEH-1332	México	Hidalgo	20.7920	-99.4080
S. grammicus	CIB-UAEH	CIB-UAEH-549	México	Puebla	19.6961	-98.0761
S. grammicus	CIB-UAEH	CIB-UAEH-233	México	Puebla	20.3100	-98.2740
S. grammicus	CIB-UAEH	CIB-UAEH-628	México	San Luis Potosí	23.5111	-100.6206
S. grammicus	CIB-UAEH	CIB-UAEH-605	México	Tamaulipas	23.5580	-99.6880
S. grammicus	BUAP	not recorded / 168	México	Oaxaca	17.6060	-96.7640
S. grammicus	ENCB	15179	México	Aguascalientes	21.9440	-102.0860
S. grammicus	ENCB	16413	México	Distrito Federal	19.5650	-99.1250
S. grammicus	ENCB	407	México	Distrito Federal	19.3680	-99.0870
S. grammicus	ENCB	17023	México	Distrito Federal	19.3110	-99.0410
S. grammicus	ENCB	908	México	Distrito Federal	19.2670	-99.2540
S. grammicus	ENCB	6893	México	Distrito Federal	19.2670	-99.2540
S. grammicus	ENCB	13952	México	Distrito Federal	19.3000	-99.2167
S. grammicus	ENCB	13724	México	Distrito Federal	19.1040	-99.1170
S. grammicus	ENCB	404	México	Distrito Federal	19.2750	-99.1390
S. grammicus	ENCB	1317	México	Distrito Federal	19.2420	-99.2110
S. grammicus	ENCB	1674	México	Distrito Federal	19.1360	-99.1430
S. grammicus	ENCB	7372	México	Distrito Federal	19.1620	-99.2480
S. grammicus	ENCB	7375	México	Distrito Federal	19.1620	-99.2480
S. grammicus	ENCB	15287	México	Distrito Federal	19.2750	-99.1490
S. grammicus	ENCB	6965	México	Distrito Federal	19.4400	-99.0590
S. grammicus	ENCB	9489	México	Durango	23.4370	-104.2790
S. grammicus	ENCB	9497	México	Durango	23.4230	-104.2900
S. grammicus	ENCB	9500	México	Durango	23.4140	-104.2900
S. grammicus	ENCB	9732	México	Durango	23.3870	-104.2510
S. grammicus	ENCB	9952	México	Durango	23.4190	-104.2610
S. grammicus	ENCB	9503	México	Durango	23.3980	-104.2400
S. grammicus	ENCB	9939	México	Durango	23.4480	-104.2500
S. grammicus	ENCB	9945	México	Durango	23.4180	-104.2400
S. grammicus	ENCB	9950	México	Durango	23.4130	-104.2050
S. grammicus	ENCB	5423	México	México	19.1280	-98.6300
S. grammicus	ENCB	10820	México	México	19.0850	-99.3310
S. grammicus	ENCB	7376	México	México	19.1620	-100.1420
S. grammicus	ENCB	11650	México	Hidalgo	21.0540	-98.3350
S. grammicus	ENCB	12214	México	Hidalgo	20.2330	-99.5660
S. grammicus	ENCB	11649	México	Hidalgo	20.7230	-98.9490
S. grammicus	ENCB	3147	México	Hidalgo	20.0370	-98.6360
S. grammicus	ENCB	5075	México	Hidalgo	20.0170	-98.6360
S. grammicus	ENCB	10632	México	Hidalgo	20.2040	-98.5770
S. grammicus	ENCB	12213	México	Hidalgo	20.3750	-99.6510
S. grammicus	ENCB	6574	México	Hidalgo	20.3240	-98.3710
S. grammicus	ENCB	10737	México	Hidalgo	20.2580	-98.3930
S. grammicus	ENCB	4075	México	Hidalgo	20.1960	-98.7560
S. grammicus	ENCB	6247	México	Hidalgo	20.2000	-98.7370
S. grammicus	ENCB	6667	México	Hidalgo	20.1750	-98.7720
S. grammicus	ENCB	11265	México	Hidalgo	20.2170	-99.2000
S. grammicus	ENCB	6665	México	Hidalgo	20.1600	-98.8000
S. grammicus	ENCB	3146	México	Hidalgo	20.0350	-98.5320
S. grammicus	ENCB	498	México	Hidalgo	20.2090	-99.2730
S. grammicus	ENCB	12891	México	Hidalgo	19.8720	-98.9660
S. grammicus	ENCB	16405	México	Hidalgo	19.9840	-99.3280
S. grammicus	ENCB	3145	México	Hidalgo	19.9520	-98.8410
S. grammicus	ENCB	16158	México	Hidalgo	20.0010	-98.9400
S. grammicus	ENCB	3219	México	Hidalgo	19.9170	-98.5720
S. grammicus	ENCB	3220	México	Hidalgo	19.9170	-98.5720
S. grammicus	ENCB	14311	México	Jalisco	21.9010	-103.8390
S. grammicus	ENCB	12171	México	Morelos	19.0280	-99.2670
S. grammicus	ENCB	5030	México	Morelos	18.9180	-99.1440
S. grammicus	ENCB	14073	México	Morelos	18.9490	-98.6810
S. grammicus	ENCB	15344	México	Morelos	18.9440	-98.6810
S. grammicus	ENCB	615	México	Oaxaca	17.0680	-96.7200
S. grammicus	ENCB	6085	México	Oaxaca	17.4460	-96.5030
S. grammicus	ENCB	5449	México	Oaxaca	17.7500	-96.8220
S. grammicus	ENCB	15925	México	Oaxaca	17.8360	-96.7830
S. grammicus	ENCB	12475	México	Oaxaca	17.1080	-96.7690
S. grammicus	ENCB	9649	México	Oaxaca	17.8160	-96.0590
S. grammicus	ENCB	5375	México	Puebla	18.8620	-98.4380
S. grammicus	ENCB	1012	México	Puebla	19.0600	-98.6190
S. grammicus	ENCB	5378	México	Puebla	19.0760	-98.4870
S. grammicus	ENCB	13947	México	Puebla	19.3190	-98.4690
S. grammicus	ENCB	5376	México	Puebla	18.9620	-98.4600
S. grammicus	ENCB	5388	México	Puebla	18.9860	-98.4810
S. grammicus	ENCB	10821	México	Puebla	19.0870	-97.3220

S. grammicus	ENCB	10831	México	Puebla	19.0740	-97.2840
S. grammicus	ENCB	13935	México	Puebla	19.3780	-98.6420
S. grammicus	ENCB	13954	México	Puebla	19.3030	-98.6430
S. grammicus	ENCB	13652	México	Puebla	18.8920	-98.5720
S. grammicus	ENCB	8148	México	San Luis Potosí	22.6940	-101.6710
S. grammicus	ENCB	5689	México	Tamaulipas	23.7510	-99.8180
S. grammicus	ENCB	13614	México	Tlaxcala	19.3370	-98.1880
S. grammicus	ENCB	6305	México	Tlaxcala	19.5500	-98.5670
S. grammicus	ENCB	13607	México	Tlaxcala	19.2920	-98.0310
S. grammicus	ENCB	13794	México	Tlaxcala	19.2410	-98.0110
S. grammicus	ENCB	6291	México	Tlaxcala	19.4770	-98.5670
S. grammicus	ENCB	9712	México	Tlaxcala	19.3170	-98.2880
S. grammicus	ENCB	13943	México	Tlaxcala	19.3170	-98.2880
S. grammicus	ENCB	13816	México	Tlaxcala	19.2540	-98.0680
S. grammicus	ENCB	345	México	Tlaxcala	19.6040	-98.3290
S. grammicus	ENCB	17410	México	Tlaxcala	19.6990	-98.2560
S. grammicus	ENCB	17415	México	Tlaxcala	19.6310	-98.1190
S. grammicus	ENCB	5022	México	Tlaxcala	19.4300	-98.1980
S. grammicus	ENCB	13810	México	Tlaxcala	19.2410	-98.0040
S. grammicus	ENCB	346	México	Veracruz	19.5990	-97.0220
S. grammicus	ENCB	6498	México	Veracruz	19.5990	-97.0220
S. grammicus	ENCB	13705	México	Veracruz	19.0800	-97.2140
S. grammicus	ENCB	13735	México	Veracruz	19.0840	-97.1760
S. grammicus	ENCB	13742	México	Veracruz	19.0920	-97.2110
S. grammicus	ENCB	13746	México	Veracruz	19.1030	-97.2000
S. grammicus	ENCB	5027	México	Veracruz	18.9175	-97.1969
S. grammicus	ENCB	13682	México	Veracruz	19.0380	-97.2000
S. grammicus	ENCB	5080	México	Veracruz	19.5600	-97.2420
S. grammicus	ENCB	5090	México	Veracruz	19.5600	-97.2420
S. grammicus	ENCB	5113	México	Veracruz	19.5600	-97.1700
S. grammicus	ENCB	5139	México	Veracruz	19.4980	-97.1790
S. grammicus	ENCB	14223	México	Zacatecas	21.6750	-102.8300
S. grammicus	ENCB	14103	México	Zacatecas	22.8780	-103.6760
S. grammicus	ENCB	14766	México	Zacatecas	22.8200	-103.7330
S. grammicus	ENCB	14767	México	Zacatecas	22.7717	-102.6044
S. grammicus	UAEM	819	México	Morelos	18.9670	-99.2840
S. grammicus	UAEM	2475	México	Morelos	19.0370	-99.2830
S. grammicus	UAEM	2516	México	Morelos	19.0360	-99.3010
S. grammicus	UAEM	2670	México	Veracruz	18.9622	-97.1547
S. grammicus	UANL	4786	México	Tamaulipas	23.6150	-99.7030
S. grammicus	MZFC	5790	México	Chihuahua	27.8830	-107.5830
S. grammicus	MZFC	6000	México	Distrito Federal	19.3160	-99.1830
S. grammicus	MZFC	107	México	Distrito Federal	19.2830	-99.2660
S. grammicus	MZFC	5770	México	Distrito Federal	19.3330	-99.2330
S. grammicus	MZFC	611	México	Distrito Federal	19.2330	-99.2330
S. grammicus	MZFC	1939	México	Distrito Federal	19.2160	-99.2000
S. grammicus	MZFC	1950	México	México	20.0233	-99.8683
S. grammicus	MZFC	3288	México	México	19.3330	-98.7000
S. grammicus	MZFC	1562	México	México	19.0500	-99.3160
S. grammicus	MZFC	3485	México	México	18.8617	-100.4483
S. grammicus	MZFC	3486	México	México	18.8660	-100.4500
S. grammicus	MZFC	3198	México	México	19.1330	-99.3000
S. grammicus	MZFC	4176	México	México	19.2830	-99.6500
S. grammicus	MZFC	930	México	México	19.1660	-100.1160
S. grammicus	MZFC	4322	México	México	19.1933	-100.1294
S. grammicus	MZFC	6293	México	Guanajuato	21.5500	-101.0830
S. grammicus	MZFC	6901	México	Guanajuato	21.3000	-100.0500
S. grammicus	MZFC	not recorded / 1271	México	Guerrero	17.5750	-99.6920
S. grammicus	MZFC	4237	México	Hidalgo	20.1830	-98.4500
S. grammicus	MZFC	4247	México	Hidalgo	20.3000	-98.9160
S. grammicus	MZFC	98	México	Hidalgo	20.1000	-99.1330
S. grammicus	MZFC	1949	México	Hidalgo	20.2330	-99.5500
S. grammicus	MZFC	1986	México	Hidalgo	20.2660	-98.9000
S. grammicus	MZFC	1987	México	Hidalgo	20.2500	-98.8830
S. grammicus	MZFC	4227	México	Hidalgo	20.2330	-98.8330
S. grammicus	MZFC	4229	México	Hidalgo	20.2330	-98.8500
S. grammicus	MZFC	4248	México	Hidalgo	20.2500	-98.8660
S. grammicus	MZFC	4851	México	Hidalgo	19.9660	-98.7000
S. grammicus	MZFC	1985	México	Hidalgo	20.2000	-98.5160
S. grammicus	MZFC	4232	México	Hidalgo	20.1660	-98.5160
S. grammicus	MZFC	4239	México	Hidalgo	20.2000	-98.5000
S. grammicus	MZFC	4849	México	Hidalgo	20.2830	-98.3500
S. grammicus	MZFC	4842	México	Hidalgo	20.0660	-98.7000
S. grammicus	MZFC	1990	México	Hidalgo	20.2160	-98.7330
S. grammicus	MZFC	4245	México	Hidalgo	20.2000	-98.7330

S. grammicus	MZFC	4233	México	Hidalgo	20.1500	-98.6160
S. grammicus	MZFC	4246	México	Hidalgo	20.1660	-98.7660
S. grammicus	MZFC	1982	México	Hidalgo	20.0500	-98.4660
S. grammicus	MZFC	4241	México	Hidalgo	20.0330	-98.4660
S. grammicus	MZFC	4226	México	Hidalgo	20.0330	-98.5330
S. grammicus	MZFC	4841	México	Hidalgo	20.0500	-98.5000
S. grammicus	MZFC	4846	México	Hidalgo	20.0500	-98.5160
S. grammicus	MZFC	104	México	Hidalgo	19.9831	-99.4708
S. grammicus	MZFC	4850	México	Hidalgo	19.8500	-98.9660
S. grammicus	MZFC	4231	México	Hidalgo	20.0660	-98.4830
S. grammicus	MZFC	4838	México	Hidalgo	20.0830	-98.3660
S. grammicus	MZFC	3459	México	Hidalgo	20.7160	-98.7000
S. grammicus	MZFC	1952	México	Hidalgo	20.6500	-98.6830
S. grammicus	MZFC	1944	México	Michoacán	20.1910	-100.2640
S. grammicus	MZFC	1951	México	Michoacán	20.1320	-100.1950
S. grammicus	MZFC	MZFC-12007	México	Michoacán	19.5240	-100.2570
S. grammicus	MZFC	3252	México	Morelos	19.0660	-99.3000
S. grammicus	MZFC	4482	México	Oaxaca	17.5830	-96.4830
S. grammicus	MZFC	4484	México	Oaxaca	17.5500	-96.4500
S. grammicus	MZFC	4487	México	Oaxaca	17.5830	-96.4660
S. grammicus	MZFC	101	México	Oaxaca	17.1000	-96.7160
S. grammicus	MZFC	102	México	Puebla	18.7660	-97.4000
S. grammicus	MZFC	3262	México	Puebla	18.3330	-98.6660
S. grammicus	MZFC	3263	México	Puebla	18.3330	-98.6500
S. grammicus	MZFC	3458	México	Puebla	19.7500	-97.9830
S. grammicus	MZFC	3568	México	Puebla	19.7500	-98.0500
S. grammicus	MZFC	4839	México	Puebla	20.3160	-98.2330
S. grammicus	MZFC	6418	México	Puebla	19.3000	-97.4000
S. grammicus	MZFC	3231	México	Puebla	20.1160	-98.1000
S. grammicus	MZFC	3203	México	Puebla	18.3330	-98.6830
S. grammicus	MZFC	6036	México	Puebla	18.7000	-98.2830
S. grammicus	MZFC	226	México	Querétaro	20.1850	-99.9930
S. grammicus	MZFC	9345	México	Querétaro	20.1820	-100.1630
S. grammicus	MZFC	9346	México	Querétaro	20.1610	-100.1670
S. grammicus	MZFC	1943	México	Querétaro	20.7720	-99.7110
S. grammicus	MZFC	8396	México	Querétaro	20.8540	-99.5880
S. grammicus	MZFC	9357	México	Querétaro	20.9240	-100.1720
S. grammicus	MZFC	9670	México	Querétaro	20.9160	-100.2020
S. grammicus	MZFC	9672	México	Querétaro	20.9200	-100.1950
S. grammicus	MZFC	9673	México	Querétaro	20.9300	-100.1820
S. grammicus	MZFC	9135	México	Querétaro	21.3910	-99.1910
S. grammicus	MZFC	9137	México	Querétaro	21.2970	-99.1790
S. grammicus	MZFC	9303	México	Querétaro	21.2860	-99.1790
S. grammicus	MZFC	9306	México	Querétaro	21.3200	-99.1680
S. grammicus	MZFC	9138	México	Querétaro	21.1400	-99.6880
S. grammicus	MZFC	6877	México	Querétaro	21.1420	-99.6210
S. grammicus	MZFC	9669	México	Querétaro	20.9110	-99.5450
S. grammicus	MZFC	9455	México	Querétaro	20.9730	-100.0230
S. grammicus	MZFC	5764	México	Tlaxcala	19.4660	-98.4160
S. grammicus	MZFC	3455	México	Tlaxcala	19.6660	-98.1000
S. grammicus	MZFC	6422	México	Veracruz	19.6000	-97.0330
S. grammicus	MZFC	732	México	Veracruz	18.7160	-97.3000
S. grammicus	MZFC	1820	México	Veracruz	20.6830	-98.0160
S. grammicus	MZFC	105	México	Zacatecas	23.1660	-103.0160
S. grammicus	FMNH	104563-564	México	Chihuahua	27.8660	-107.9330
S. grammicus	FMNH	65489	México	Distrito Federal	19.2660	-99.3160
S. grammicus	FMNH	32533-534	México	México	20.0500	-99.7160
S. grammicus	FMNH	1300	México	México	19.1160	-98.7660
S. grammicus	FMNH	65467-480	México	México	19.0330	-98.6330
S. grammicus	FMNH	1002	México	México	19.2583	-98.8967
S. grammicus	FMNH	116263	México	México	19.2917	-100.0958
S. grammicus	FMNH	111747	México	México	19.3517	-98.6711
S. grammicus	FMNH	32496-497	México	México	19.3500	-98.6660
S. grammicus	FMNH	32532	México	México	19.2903	-99.5117
S. grammicus	FMNH	116265	México	México	19.0489	-99.3175
S. grammicus	FMNH	32454	México	México	19.2931	-99.5333
S. grammicus	FMNH	32498	México	México	19.2830	-99.5160
S. grammicus	FMNH	1004	México	México	19.5000	-98.9500
S. grammicus	FMNH	65461-466	México	México	19.2000	-99.8160
S. grammicus	FMNH	65481-486	México	México	19.2330	-99.7830
S. grammicus	FMNH	65487-488	México	México	19.2500	-99.7660
S. grammicus	FMNH	32531	México	Guanajuato	21.1000	-101.1660
S. grammicus	FMNH	32397-399	México	Hidalgo	20.1160	-98.9830
S. grammicus	FMNH	70801	México	Hidalgo	20.8500	-99.2330
S. grammicus	FMNH	32407-446	México	Michoacán	19.4160	-102.0500

S. grammicus	FMNH	1280	México	Morelos	18.8160	-98.9500
S. grammicus	FMNH	32499-508	México	Morelos	19.0500	-99.2330
S. grammicus	FMNH	32509-510	México	Morelos	19.0160	-99.2500
S. grammicus	FMNH	32400-401	México	Morelos	19.0160	-99.1830
S. grammicus	FMNH	32476-478	México	Morelos	19.0160	-99.1660
S. grammicus	FMNH	32495	México	Puebla	18.7330	-97.4160
S. grammicus	FMNH	32455-458	México	Puebla	18.6500	-97.3830
S. grammicus	FMNH	1003	México	Puebla	19.0500	-98.2000
S. grammicus	FMNH	1341	México	Puebla	19.8160	-97.3660
S. grammicus	FMNH	32479	México	Puebla	20.0330	-97.6160
S. grammicus	FMNH	104565-566	México	San Luis Potosí	22.0160	-101.2500
S. grammicus	FMNH	32459-467	México	Veracruz	19.6330	-97.1660
S. grammicus	CNAR	6131	México	Aguascalientes	22.1600	-102.5600
S. grammicus	CNAR	4876	México	Chihuahua	26.0700	-106.9600
S. grammicus	CNAR	7023	México	Chihuahua	28.3700	-107.9100
S. grammicus	CNAR	4661	México	Coahuila	27.8000	-101.1500
S. grammicus	CNAR	4663	México	Coahuila	27.8500	-101.1100
S. grammicus	CNAR	7024	México	Coahuila	25.4200	-100.9700
S. grammicus	CNAR	6843	México	Distrito Federal	19.3200	-99.2000
S. grammicus	CNAR	7180	México	Distrito Federal	19.5200	-99.1400
S. grammicus	CNAR	7473	México	Distrito Federal	19.3500	-99.0000
S. grammicus	CNAR	6838	México	Distrito Federal	19.2100	-99.2700
S. grammicus	CNAR	6847	México	Distrito Federal	19.2600	-99.2400
S. grammicus	CNAR	7784	México	Distrito Federal	19.2300	-99.2600
S. grammicus	CNAR	8655	México	Distrito Federal	19.2100	-99.2600
S. grammicus	CNAR	8676	México	Distrito Federal	19.3000	-99.2400
S. grammicus	CNAR	8713	México	Distrito Federal	19.2200	-99.2700
S. grammicus	CNAR	8776	México	Distrito Federal	19.2300	-99.2700
S. grammicus	CNAR	8991	México	Distrito Federal	19.2200	-99.2800
S. grammicus	CNAR	6839	México	Distrito Federal	19.2700	-99.2100
S. grammicus	CNAR	6841	México	Distrito Federal	19.2000	-99.2500
S. grammicus	CNAR	9797	México	Distrito Federal	19.2900	-99.1400
S. grammicus	CNAR	7132	México	Durango	24.0100	-104.7500
S. grammicus	CNAR	7576	México	Durango	23.8700	-105.1500
S. grammicus	CNAR	6262	México	Durango	26.5900	-104.0600
S. grammicus	CNAR	7131	México	Durango	22.6200	-104.2100
S. grammicus	CNAR	7575	México	Durango	23.8400	-105.3200
S. grammicus	CNAR	1840	México	Durango	23.3864	-104.2464
S. grammicus	CNAR	7654	México	México	19.1083	-99.7617
S. grammicus	CNAR	6835	México	México	19.7400	-99.2300
S. grammicus	CNAR	8865	México	México	19.1300	-99.2900
S. grammicus	CNAR	8993	México	México	19.1314	-99.2833
S. grammicus	CNAR	10848	México	México	19.1367	-99.2917
S. grammicus	CNAR	10873	México	México	19.1350	-99.2917
S. grammicus	CNAR	10894	México	México	19.1600	-99.3200
S. grammicus	CNAR	7659	México	México	19.1900	-99.8100
S. grammicus	CNAR	6478	México	Guerrero	17.2600	-98.7400
S. grammicus	CNAR	6479	México	Guerrero	17.2500	-98.7600
S. grammicus	CNAR	7517	México	Hidalgo	20.1700	-98.4400
S. grammicus	CNAR	7527	México	Hidalgo	20.0900	-99.1100
S. grammicus	CNAR	7526	México	Hidalgo	20.2400	-99.5500
S. grammicus	CNAR	7525	México	Hidalgo	20.3700	-99.6500
S. grammicus	CNAR	7183	México	Hidalgo	20.0800	-98.7100
S. grammicus	CNAR	7516	México	Hidalgo	20.1700	-98.6500
S. grammicus	CNAR	7660	México	Hidalgo	20.0800	-98.7800
S. grammicus	CNAR	7192	México	Hidalgo	20.1200	-98.8300
S. grammicus	CNAR	7182	México	Hidalgo	20.4000	-98.2000
S. grammicus	CNAR	7190	México	Hidalgo	20.0500	-98.4600
S. grammicus	CNAR	7507	México	Hidalgo	20.0600	-98.4800
S. grammicus	CNAR	7512	México	Hidalgo	20.0500	-98.4800
S. grammicus	CNAR	7522	México	Hidalgo	20.0500	-98.4800
S. grammicus	CNAR	AR9107	México	Hidalgo	20.0500	-98.4830
S. grammicus	CNAR	7508	México	Hidalgo	20.0600	-98.5100
S. grammicus	CNAR	7509	México	Hidalgo	20.0500	-98.5000
S. grammicus	CNAR	7524	México	Hidalgo	20.0400	-98.5200
S. grammicus	CNAR	7574	México	Hidalgo	21.0100	-98.9500
S. grammicus	CNAR	1854	México	Hidalgo	19.8700	-98.9500
S. grammicus	CNAR	7193	México	Hidalgo	20.0700	-98.4800
S. grammicus	CNAR	11081	México	Jalisco	21.7100	-102.7100
S. grammicus	CNAR	AR11379	México	Jalisco	21.6830	-102.5830
S. grammicus	CNAR	7166	México	Michoacán	19.5200	-101.6100
S. grammicus	CNAR	7165	México	Michoacán	19.4100	-101.5400
S. grammicus	CNAR	AR8870	México	Michoacán	19.3500	-101.5330
S. grammicus	CNAR	CNAR-IBH-16429	México	Michoacán	19.3930	-100.2810
S. grammicus	CNAR	1945	México	Morelos	19.0380	-99.2790

S. grammicus	CNAR	6356	México	Morelos	19.0300	-99.2000
S. grammicus	CNAR	1998	México	Morelos	18.9680	-99.1840
S. grammicus	CNAR	9277	México	Morelos	19.0000	-99.0500
S. grammicus	CNAR	1695	México	Nuevo León	24.7833	-100.2067
S. grammicus	CNAR	7025	México	Nuevo León	26.4200	-99.5300
S. grammicus	CNAR	4664	México	Nuevo León	25.4700	-100.6300
S. grammicus	CNAR	6726	México	Oaxaca	17.3300	-96.4700
S. grammicus	CNAR	6729	México	Oaxaca	17.2600	-96.6900
S. grammicus	CNAR	7590	México	Oaxaca	17.2600	-96.6400
S. grammicus	CNAR	6255	México	Oaxaca	16.9300	-97.0500
S. grammicus	CNAR	6728	México	Oaxaca	17.4500	-96.5000
S. grammicus	CNAR	6723	México	Oaxaca	16.9400	-97.0100
S. grammicus	CNAR	6725	México	Oaxaca	16.9400	-97.0000
S. grammicus	CNAR	6727	México	Oaxaca	16.9300	-97.0000
S. grammicus	CNAR	6730	México	Oaxaca	16.9300	-96.9400
S. grammicus	CNAR	6251	México	Oaxaca	17.0400	-97.0900
S. grammicus	CNAR	6731	México	Oaxaca	17.2100	-97.0200
S. grammicus	CNAR	7653	México	Puebla	20.1600	-98.1500
S. grammicus	CNAR	7040	México	Querétaro	20.0900	-100.0700
S. grammicus	CNAR	7658	México	Querétaro	20.8900	-99.6600
S. grammicus	CNAR	7044	México	Querétaro	20.9230	-99.6010
S. grammicus	CNAR	7039	México	Querétaro	20.4010	-100.0170
S. grammicus	CNAR	7043	México	Querétaro	20.5310	-99.8880
S. grammicus	CNAR	4751	México	San Luis Potosí	23.6900	-100.8900
S. grammicus	CNAR	4750	México	San Luis Potosí	23.2400	-100.9500
S. grammicus	CNAR	2728	México	San Luis Potosí	22.5200	-101.0200
S. grammicus	CNAR	4596	México	Tamaulipas	23.9600	-98.6600
S. grammicus	CNAR	4595	México	Tamaulipas	23.6300	-99.0500
S. grammicus	CNAR	7164	México	Tlaxcala	19.2000	-98.1500
S. grammicus	CNAR	6349	México	Veracruz	19.6500	-97.0900
S. grammicus	CNAR	3217	México	Zacatecas	24.1700	-101.8000
S. grammicus	CNAR	7577	México	Zacatecas	22.2900	-101.6100
S. grammicus	CNAR	7578	México	Zacatecas	22.8200	-103.6000
S. grammicus	KU	43669	México	Chihuahua	30.0830	-108.4160
S. grammicus	KU	44181	México	Chihuahua	28.0500	-108.4700
S. grammicus	KU	38111	México	Coahuila	27.2160	-101.0830
S. grammicus	KU	33603	México	Coahuila	25.3660	-103.3330
S. grammicus	KUMNH	62458	México	México	20.0719	-99.8500
S. grammicus	KUMNH	62459	México	México	19.8594	-99.8561
S. grammicus	KUMNH	43654	México	México	19.3319	-100.2472
S. grammicus	KUMNH	23919	México	México	19.3472	-98.7525
S. grammicus	KUMNH	38117	México	México	19.1278	-99.7806
S. grammicus	KU	43671	México	Michoacán	20.2000	-102.5160
S. grammicus	KU	29761	México	Michoacán	19.5160	-102.2330
S. grammicus	KU	68109	México	Nuevo León	25.2850	-100.0190
S. grammicus	KU	38110	México	Nuevo León	24.3160	-99.8830
S. grammicus	KU	92598	México	Nuevo León	25.1190	-100.1600
S. grammicus	KU	92597	México	Nuevo León	25.4280	-100.1290
S. grammicus	KU	59623	México	Puebla	18.7690	-97.5360
S. grammicus	KU	26702	México	Veracruz	19.7500	-97.3000
S. grammicus	LSUMNS	30199	México	México	19.3500	-98.7111
S. grammicus	LSUMNS	36183	México	México	19.0133	-98.8008
S. grammicus	LSUMNS	48966	México	Morelos	19.0892	-99.2844
S. grammicus	LSUMNS	19801	México	Tamaulipas	23.0360	-99.1900
S. grammicus	MCZ	R-136651	México	Coahuila	27.8830	-101.5160
S. grammicus	MCZ	R-136626	México	Coahuila	25.2160	-101.5330
S. grammicus	MCZ	R-136781	México	México	19.7500	-98.6660
S. grammicus	MCZ	R-136611	México	Michoacán	19.7830	-101.9500
S. grammicus	MCZ	R-136604	México	Michoacán	19.8500	-102.0660
S. grammicus	MCZ	R-136596	México	Nuevo León	26.0870	-99.6160
S. grammicus	MCZ	R-136570	México	Nuevo León	25.7056	-99.3500
S. grammicus	MCZ	R-136593	México	Nuevo León	26.1910	-100.4800
S. grammicus	MCZ	46758-761	México	Oaxaca	17.8330	-96.6660
S. grammicus	MCZ	R-136847	México	Puebla	19.1380	-97.5410
S. grammicus	MCZ	R-136588	México	San Luis Potosí	22.4000	-101.1660
S. grammicus	MCZ	R-136595	México	San Luis Potosí	22.4500	-99.5500
S. grammicus	MVZ	68832	México	Chihuahua	29.4944	-106.7871
S. grammicus	MVZ	70759	México	Chihuahua	29.4851	-106.7704
S. grammicus	MVZ	84642	México	Chihuahua	29.4807	-106.7717
S. grammicus	MVZ	46671	México	Chihuahua	29.9107	-108.3333
S. grammicus	MVZ	72912	México	Chihuahua	29.3920	-106.8980
S. grammicus	MVZ	66003	México	Chihuahua	28.5681	-108.1481
S. grammicus	MVZ	58325	México	Coahuila	28.9770	-102.5510
S. grammicus	MVZ	131509	México	Distrito Federal	19.2353	-99.2889
S. grammicus	MVZ	197656	México	Distrito Federal	19.2350	-99.2880

S. grammicus	MVZ	36774	México	Distrito Federal	19.4039	-99.1953
S. grammicus	MVZ	197616	México	Distrito Federal	19.2330	-99.1990
S. grammicus	MVZ	197654	México	Distrito Federal	19.2333	-99.1997
S. grammicus	MVZ	36775	México	México	19.0569	-98.6778
S. grammicus	MVZ	36814	México	México	19.1270	-98.6860
S. grammicus	MVZ	144148	México	México	19.0847	-98.6394
S. grammicus	MVZ	104055	México	México	19.1330	-99.7330
S. grammicus	MVZ	144125	México	México	19.1331	-99.7331
S. grammicus	MVZ	71951	México	México	19.3389	-98.7561
S. grammicus	MVZ	137089	México	México	19.3425	-98.7644
S. grammicus	MVZ	137092	México	México	19.3206	-98.7453
S. grammicus	MVZ	137127	México	México	19.3200	-98.7450
S. grammicus	MVZ	104056	México	México	19.3217	-99.3739
S. grammicus	MVZ	144127	México	México	19.0728	-99.3667
S. grammicus	MVZ	8854	México	México	19.9090	-99.1440
S. grammicus	MVZ	8858	México	México	19.9094	-99.1447
S. grammicus	MVZ	71952	México	México	19.4308	-100.1728
S. grammicus	MVZ	71954	México	México	19.4508	-100.1867
S. grammicus	MVZ	71966	México	México	19.4528	-100.1833
S. grammicus	MVZ	144107	México	México	19.5350	-100.0017
S. grammicus	MVZ	36813	México	México	19.2161	-99.7703
S. grammicus	MVZ	144121	México	México	19.1361	-99.7972
S. grammicus	MVZ	144124	México	México	19.1333	-99.7906
S. grammicus	MVZ	186503	México	Hidalgo	20.9114	-99.2072
S. grammicus	MVZ	114700	México	Hidalgo	20.7150	-98.6420
S. grammicus	MVZ	8857	México	Hidalgo	19.9020	-98.7020
S. grammicus	MVZ	146915	México	Hidalgo	20.8800	-99.2300
S. grammicus	MVZ	56299	México	Michoacán	19.6687	-100.8168
S. grammicus	MVZ	61203	México	Michoacán	19.6631	-100.8521
S. grammicus	MVZ	71956	México	Michoacán	19.7647	-100.6202
S. grammicus	MVZ	36799	México	Morelos	19.0220	-99.2240
S. grammicus	MVZ	71256	México	Morelos	19.0520	-99.3020
S. grammicus	MVZ	78274	México	Morelos	19.0281	-99.2342
S. grammicus	MVZ	78388	México	Morelos	19.0280	-99.2340
S. grammicus	MVZ	109514	México	Nuevo León	25.8860	-100.2190
S. grammicus	MVZ	109511	México	Nuevo León	24.6560	-100.1790
S. grammicus	MVZ	109512	México	Nuevo León	24.5116	-100.0016
S. grammicus	MVZ	144116	México	Nuevo León	24.8760	-100.2200
S. grammicus	MVZ	144117	México	Nuevo León	24.8783	-100.1869
S. grammicus	MVZ	144118	México	Nuevo León	24.8683	-100.2317
S. grammicus	MVZ	131504	México	Oaxaca	17.5819	-96.4761
S. grammicus	MVZ	144146	México	Oaxaca	17.5810	-96.4761
S. grammicus	MVZ	144132	México	Oaxaca	16.9222	-96.9000
S. grammicus	MVZ	131497	México	Oaxaca	17.2394	-96.6392
S. grammicus	MVZ	131499	México	Oaxaca	17.1128	-97.6119
S. grammicus	MVZ	164711	México	Oaxaca	17.1120	-97.6110
S. grammicus	MVZ	144143	México	Oaxaca	17.4464	-96.5025
S. grammicus	MVZ	144139	México	Oaxaca	17.3333	-96.7631
S. grammicus	MVZ	144140	México	Oaxaca	17.5270	-97.2690
S. grammicus	MVZ	144142	México	Oaxaca	17.5272	-97.2692
S. grammicus	MVZ	162289	México	Oaxaca	17.5453	-96.5169
S. grammicus	MVZ	162290	México	Oaxaca	17.5450	-96.5169
S. grammicus	MVZ	114717	México	Oaxaca	17.2097	-96.6481
S. grammicus	MVZ	131498	México	Oaxaca	17.2358	-96.6370
S. grammicus	MVZ	144133	México	Oaxaca	17.2090	-96.6480
S. grammicus	MVZ	144137	México	Oaxaca	17.1883	-96.6011
S. grammicus	MVZ	137094	México	Oaxaca	17.5770	-96.4986
S. grammicus	MVZ	131505	México	Puebla	19.6960	-98.0760
S. grammicus	MVZ	144131	México	Puebla	19.0600	-98.6190
S. grammicus	MVZ	104119	México	Puebla	19.3440	-98.6420
S. grammicus	MVZ	69625	México	Tamaulipas	24.0100	-98.9400
S. grammicus	MVZ	109515	México	Tamaulipas	24.8520	-98.1540
S. grammicus	MVZ	66019	US	Texas	27.0097	-99.3856
S. grammicus	MVZ	131510	México	Tlaxcala	19.2408	-98.1028
S. grammicus	MVZ	196097	México	Veracruz	19.6100	-97.0270
S. grammicus	MVZ	146917	México	Veracruz	19.7717	-97.1845
S. grammicus	MVZ	137086	México	Veracruz	18.9170	-97.1960
S. grammicus	MVZ	114719	México	Veracruz	18.9620	-97.1540
S. grammicus	MVZ	106264	México	Veracruz	19.4980	-97.0980
S. grammicus	MVZ	106263	México	Veracruz	19.6280	-97.1330
S. grammicus	MVZ	114709	México	Veracruz	19.5830	-97.1130
S. grammicus	MVZ	172130	México	Veracruz	19.5706	-97.1047
S. grammicus	MVZ	61207	México	Veracruz	19.6561	-97.2453
S. grammicus	MVZ	144147	México	Veracruz	19.6560	-97.2450
S. grammicus	MVZ	80039	México	Zacatecas	22.7710	-102.6040

S. grammicus	ND	S/N / 52420	México	Distrito Federal	19.4330	-99.0830
S. grammicus	ND	S/N / 53673	México	Durango	23.7660	-105.4000
S. grammicus	ND	not recorded / 850	México	México	19.0819	-98.6475
S. grammicus	ND	NA / 1224	México	México	19.3580	-100.2380
S. grammicus	ND	NA / 1244	México	México	19.3770	-100.2280
S. grammicus	ND	NA / 1255	México	México	19.3800	-100.2250
S. grammicus	ND	NA / 1307	México	México	19.3970	-100.2550
S. grammicus	ND	NA / 2042	México	México	19.4000	-100.2270
S. grammicus	ND	NA / 2043	México	México	19.4010	-100.2330
S. grammicus	ND	NA / 2054	México	México	19.3750	-100.2260
S. grammicus	ND	NA / 2057	México	México	19.3770	-100.2500
S. grammicus	ND	NA / 2058	México	México	19.3840	-100.2500
S. grammicus	ND	NA / 2059	México	México	19.3840	-100.2520
S. grammicus	ND	NA / 2104	México	México	19.3650	-100.2400
S. grammicus	ND	NA / 232	México	México	19.3780	-100.2220
S. grammicus	ND	NA / 608	México	México	19.3910	-100.2680
S. grammicus	ND	NA / 893	México	México	19.3450	-100.2450
S. grammicus	ND	NA / 910	México	México	19.3750	-100.2560
S. grammicus	ND	NA / 974	México	México	19.3740	-100.2240
S. grammicus	ND	NA / 977	México	México	19.3650	-100.2310
S. grammicus	ND	NA / 979	México	México	19.3580	-100.2400
S. grammicus	ND	NA / 983	México	México	19.3590	-100.2410
S. grammicus	ND	NA / 984	México	México	19.3600	-100.2440
S. grammicus	ND	NA / 999	México	México	19.3800	-100.2230
S. grammicus	ND	not recorded / 844	México	México	19.0870	-99.3120
S. grammicus	ND	not recorded / 846	México	México	19.0890	-99.3150
S. grammicus	ND	S/N / 9798	México	México	18.9000	-100.1500
S. grammicus	ND	NA / 1234	México	México	19.3800	-100.2180
S. grammicus	ND	NA / 1287	México	México	19.3790	-100.2190
S. grammicus	ND	NA / 2040	México	México	19.3980	-100.2140
S. grammicus	ND	NA / 2045	México	México	19.3970	-100.2160
S. grammicus	ND	NA / 1707	México	México	19.5090	-100.2210
S. grammicus	ND	NA / 1736	México	México	19.5120	-100.2080
S. grammicus	ND	NA / 1890	México	México	19.5000	-100.2090
S. grammicus	ND	NA / 253	México	México	19.5090	-100.2290
S. grammicus	ND	S/N / 6578	México	Guanajuato	21.0830	-101.1500
S. grammicus	ND	S/N / 7179	México	Hidalgo	20.3330	-99.7330
S. grammicus	ND	NA / 1009	México	Michoacán	19.5420	-100.3000
S. grammicus	ND	NA / 1028	México	Michoacán	19.5420	-100.2990
S. grammicus	ND	NA / 1029	México	Michoacán	19.5420	-100.3470
S. grammicus	ND	NA / 1062	México	Michoacán	19.5380	-100.2770
S. grammicus	ND	NA / 1064	México	Michoacán	19.5370	-100.2800
S. grammicus	ND	NA / 1117	México	Michoacán	19.5590	-100.2690
S. grammicus	ND	NA / 1202	México	Michoacán	19.5470	-100.3310
S. grammicus	ND	NA / 1213	México	Michoacán	19.5350	-100.3000
S. grammicus	ND	NA / 1518	México	Michoacán	19.5410	-100.2720
S. grammicus	ND	NA / 1521	México	Michoacán	19.5410	-100.2710
S. grammicus	ND	NA / 1529	México	Michoacán	19.5410	-100.2880
S. grammicus	ND	NA / 1809	México	Michoacán	19.5300	-100.2510
S. grammicus	ND	NA / 1832	México	Michoacán	19.5320	-100.2480
S. grammicus	ND	NA / 1896	México	Michoacán	19.5380	-100.3510
S. grammicus	ND	NA / 1916	México	Michoacán	19.5400	-100.2860
S. grammicus	ND	NA / 1934	México	Michoacán	19.5430	-100.3000
S. grammicus	ND	NA / 2024	México	Michoacán	19.5470	-100.3300
S. grammicus	ND	NA / 2025	México	Michoacán	19.5470	-100.3330
S. grammicus	ND	NA / 2288	México	Michoacán	19.5430	-100.3480
S. grammicus	ND	NA / 2295	México	Michoacán	19.5410	-100.2730
S. grammicus	ND	NA / 2305	México	Michoacán	19.5390	-100.3350
S. grammicus	ND	NA / 2310	México	Michoacán	19.5380	-100.3530
S. grammicus	ND	NA / 2311	México	Michoacán	19.5390	-100.3500
S. grammicus	ND	NA / 2313	México	Michoacán	19.5380	-100.3520
S. grammicus	ND	NA / 520	México	Michoacán	19.5480	-100.2530
S. grammicus	ND	NA / 543	México	Michoacán	19.5420	-100.2670
S. grammicus	ND	NA / 727	México	Michoacán	19.5400	-100.3020
S. grammicus	ND	NA / 734	México	Michoacán	19.5400	-100.3000
S. grammicus	ND	NA / 749	México	Michoacán	19.5410	-100.2970
S. grammicus	ND	NA / 751	México	Michoacán	19.5410	-100.2850
S. grammicus	ND	NA / 766	México	Michoacán	19.5410	-100.2980
S. grammicus	ND	NA / 771	México	Michoacán	19.5420	-100.2840
S. grammicus	ND	NA / 805	México	Michoacán	19.5410	-100.3000
S. grammicus	ND	NA / 808	México	Michoacán	19.5390	-100.2950
S. grammicus	ND	NA / 827	México	Michoacán	19.5460	-100.3220
S. grammicus	ND	NA / 103	México	Michoacán	19.3910	-100.2830
S. grammicus	ND	NA / 1077	México	Michoacán	19.5050	-100.3200
S. grammicus	ND	NA / 1118	México	Michoacán	19.5290	-100.2490

S. grammicus	ND	NA / 1120	México	Michoacán	19.5240	-100.2350
S. grammicus	ND	NA / 1136	México	Michoacán	19.4050	-100.3070
S. grammicus	ND	NA / 1152	México	Michoacán	19.5080	-100.2660
S. grammicus	ND	NA / 1211	México	Michoacán	19.5300	-100.2340
S. grammicus	ND	NA / 1312	México	Michoacán	19.5010	-100.3010
S. grammicus	ND	NA / 1495	México	Michoacán	19.5200	-100.2400
S. grammicus	ND	NA / 1496	México	Michoacán	19.5180	-100.3100
S. grammicus	ND	NA / 1556	México	Michoacán	19.4100	-100.3060
S. grammicus	ND	NA / 1557	México	Michoacán	19.4100	-100.3080
S. grammicus	ND	NA / 1590	México	Michoacán	19.4030	-100.3030
S. grammicus	ND	NA / 1598	México	Michoacán	19.4030	-100.3100
S. grammicus	ND	NA / 1661	México	Michoacán	19.5030	-100.3210
S. grammicus	ND	NA / 1752	México	Michoacán	19.5090	-100.3250
S. grammicus	ND	NA / 1753	México	Michoacán	19.5100	-100.3180
S. grammicus	ND	NA / 1805	México	Michoacán	19.5210	-100.2430
S. grammicus	ND	NA / 1943	México	Michoacán	19.4040	-100.3040
S. grammicus	ND	NA / 1968	México	Michoacán	19.4040	-100.3050
S. grammicus	ND	NA / 2151	México	Michoacán	19.5170	-100.3140
S. grammicus	ND	NA / 2152	México	Michoacán	19.5170	-100.3000
S. grammicus	ND	NA / 2201	México	Michoacán	19.4590	-100.3260
S. grammicus	ND	NA / 2255	México	Michoacán	19.5110	-100.3170
S. grammicus	ND	NA / 2263	México	Michoacán	19.4800	-100.3240
S. grammicus	ND	NA / 255	México	Michoacán	19.5050	-100.2300
S. grammicus	ND	NA / 558	México	Michoacán	19.4070	-100.3100
S. grammicus	ND	NA / 587	México	Michoacán	19.4090	-100.2790
S. grammicus	ND	NA / 620	México	Michoacán	19.3900	-100.2820
S. grammicus	ND	NA / 626	México	Michoacán	19.3960	-100.2870
S. grammicus	ND	NA / 633	México	Michoacán	19.4140	-100.3130
S. grammicus	ND	NA / 635	México	Michoacán	19.4060	-100.3100
S. grammicus	ND	NA / 708	México	Michoacán	19.5250	-100.2540
S. grammicus	ND	NA / 88	México	Michoacán	19.4100	-100.3110
S. grammicus	ND	not recorded / 737	México	Morelos	19.0260	-99.2190
S. grammicus	ND	not recorded / 854	México	Morelos	19.0220	-99.2860
S. grammicus	ND	not recorded / 856	México	Morelos	19.0170	-99.0000
S. grammicus	ND	S/N / 17824	México	Oaxaca	17.5000	-96.4830
S. grammicus	ND	S/N / 17830	México	Oaxaca	17.5500	-96.4830
S. grammicus	ND	S/N / 17477	México	Oaxaca	17.1160	-96.4330
S. grammicus	ND	not recorded / 137	México	Oaxaca	17.7350	-97.1330
S. grammicus	ND	S/N / 17829	México	Oaxaca	17.5000	-96.5000
S. grammicus	ND	S/N / 22256	México	Puebla	19.1830	-98.3330
S. grammicus	ND	No DPhScgr213	México	San Luis Potosí	22.6690	-100.4140
S. grammicus	ND	No DPhScgr216	México	San Luis Potosí	22.8090	-100.4530
S. grammicus	ND	No DPhScgr451	México	San Luis Potosí	23.2010	-100.2710
S. grammicus	ND	No DPhScgr886	México	San Luis Potosí	22.7100	-100.5710
S. grammicus	ND	No DPhScgr887	México	San Luis Potosí	22.7110	-100.3880
S. grammicus	ND	No DPhScgr888	México	San Luis Potosí	22.7210	-100.3840
S. grammicus	ND	No DPhScgr895	México	San Luis Potosí	22.6320	-100.3840
S. grammicus	ND	S/N / 33623	México	Veracruz	19.6160	-97.0330
S. grammicus	ND	NA / 1698	México	Veracruz	19.4983	-97.0986
S. grammicus	ND	S/N / 37129	México	Veracruz	19.6330	-97.1500
S. grammicus	LACM	75312	México	Chihuahua	29.8400	-108.2600
S. grammicus	LACM	75314	México	Chihuahua	29.8300	-108.2500
S. grammicus	LACM	75315	México	Chihuahua	29.8100	-108.1800
S. grammicus	LACM	66171	México	Coahuila	25.2300	-100.4000
S. grammicus	LACM	58083	México	Distrito Federal	19.4314	-99.1300
S. grammicus	LACM	25201	México	Durango	23.7100	-104.9667
S. grammicus	LACM	99427	México	Durango	23.9167	-104.9667
S. grammicus	LACM	121554	México	Durango	23.8800	-105.0900
S. grammicus	LACM	66170	México	Durango	25.2200	-104.0000
S. grammicus	LACM	66173	México	Durango	25.2200	-104.0000
S. grammicus	LACM	76609	México	México	19.7800	-99.2200
S. grammicus	LACM	61978	México	México	19.3500	-98.7000
S. grammicus	LACM	61979	México	México	19.3500	-98.7000
S. grammicus	LACM	17360	México	Hidalgo	21.0100	-99.1900
S. grammicus	LACM	106715	México	Hidalgo	19.8210	-98.5710
S. grammicus	LACM	106729	México	Hidalgo	20.6500	-98.6500
S. grammicus	LACM	67539	México	Michoacán	19.4810	-100.3230
S. grammicus	LACM	58209	México	Morelos	19.0300	-99.2900
S. grammicus	LACM	119874	México	Morelos	19.0330	-99.0840
S. grammicus	LACM	119827	México	Nuevo León	24.3800	-99.9100
S. grammicus	LACM	119831	México	Nuevo León	24.3270	-99.9120
S. grammicus	LACM	119825	México	Nuevo León	24.6760	-100.3000
S. grammicus	LACM	119826	México	Nuevo León	24.5800	-100.0100
S. grammicus	LACM	119834	México	Nuevo León	25.7940	-100.3810
S. grammicus	LACM	65239	México	Oaxaca	17.3283	-96.4550

S. grammicus	LACM	131191	México	Oaxaca	17.0667	-96.7167
S. grammicus	LACM	119837	México	Oaxaca	16.9900	-96.0900
S. grammicus	LACM	128591	México	Oaxaca	17.5200	-97.4500
S. grammicus	LACM	129949	México	Oaxaca	17.5900	-96.4900
S. grammicus	LACM	61976	México	Oaxaca	17.5800	-96.5100
S. grammicus	LACM	119836	México	Oaxaca	17.5800	-96.5100
S. grammicus	LACM	129945	México	Oaxaca	17.5800	-96.5800
S. grammicus	LACM	129944	México	Oaxaca	17.3800	-96.9200
S. grammicus	LACM	119835	México	Oaxaca	16.9800	-96.0700
S. grammicus	LACM	119846	México	Puebla	19.1200	-97.9400
S. grammicus	LACM	119856	México	Puebla	18.8620	-98.4380
S. grammicus	LACM	119847	México	Puebla	18.6300	-97.2900
S. grammicus	LACM	119853	México	Puebla	18.8800	-97.6500
S. grammicus	LACM	17362	México	Puebla	18.5347	-97.4042
S. grammicus	LACM	109147	México	Querétaro	21.2667	-99.1500
S. grammicus	LACM	109762-109764	México	Querétaro	21.2870	-99.1760
S. grammicus	LACM	119877	México	Veracruz	18.9417	-97.0000
S. grammicus	LACM	126546	México	Veracruz	18.9500	-97.1666
S. grammicus	LACM	119872	México	Veracruz	19.0333	-97.2500
S. grammicus	LACM	119882	México	Veracruz	19.0167	-97.2500
S. grammicus	LACM	65221	México	Veracruz	19.5833	-97.1131
S. grammicus	LACM	119866	México	Veracruz	19.6289	-97.1333
S. grammicus	LACM	97558	México	Veracruz	19.5400	-96.9300
S. grammicus	USNM	346543	México	Aguascalientes	21.9670	-101.9830
S. grammicus	USNM	346535	México	Aguascalientes	22.2000	-102.5500
S. grammicus	USNM	346536	México	Aguascalientes	22.2170	-102.5670
S. grammicus	USNM	346537	México	Aguascalientes	22.2330	-102.5170
S. grammicus	USNM	346545	México	Aguascalientes	22.2170	-102.1670
S. grammicus	USNM	S/N / 44411	México	Chihuahua	29.9660	-108.3330
S. grammicus	USNM	S/N / 2080	México	Chihuahua	26.8160	-107.0660
S. grammicus	USNM	S/N / 49241	México	Coahuila	28.4330	-102.4660
S. grammicus	USNM	S/N / 49271	México	Coahuila	29.1000	-102.6500
S. grammicus	USNM	S/N / 49287	México	Coahuila	25.1160	-101.1000
S. grammicus	USNM	S/N / 9572	México	México	19.1000	-99.7500
S. grammicus	USNM	S/N / 9573	México	México	19.1160	-99.7500
S. grammicus	USNM	S/N / 9012	México	México	19.4160	-98.9500
S. grammicus	USNM	S/N / 9676	México	México	19.3160	-98.7330
S. grammicus	USNM	S/N / 9762	México	México	19.6830	-98.8660
S. grammicus	USNM	S/N / 9362	México	México	19.4160	-98.7160
S. grammicus	USNM	S/N / 9945	México	México	19.4160	-100.1160
S. grammicus	USNM	S/N / 9953	México	México	19.4500	-100.1000
S. grammicus	USNM	S/N / 9493	México	México	19.1160	-99.7660
S. grammicus	USNM	S/N / 6561	México	Guanajuato	21.0660	-101.2000
S. grammicus	USNM	S/N / 6518	México	Guanajuato	21.4660	-100.8660
S. grammicus	USNM	S/N / 52398	México	Guerrero	18.3330	-99.1160
S. grammicus	USNM	S/N / 7129	México	Hidalgo	20.1000	-98.7160
S. grammicus	USNM	S/N / 7106	México	Hidalgo	20.2000	-99.5330
S. grammicus	USNM	346542	México	Jalisco	21.7500	-102.7170
S. grammicus	USNM	S/N / 12430	México	Michoacán	19.8160	-100.3000
S. grammicus	USNM	S/N / 13045	México	Morelos	19.0830	-99.2160
S. grammicus	USNM	299657	México	Nuevo León	25.8870	-100.2191
S. grammicus	USNM	S/N / 15755	México	Nuevo León	24.9330	-100.1500
S. grammicus	USNM	S/N / 19924	México	Oaxaca	17.8500	-96.8660
S. grammicus	USNM	S/N / 17035	México	Oaxaca	17.1660	-96.6660
S. grammicus	USNM	S/N / 20014	México	Oaxaca	17.6830	-97.5830
S. grammicus	USNM	S/N / 9557	México	Puebla	19.1000	-98.4660
S. grammicus	USNM	S/N / 34813	México	Puebla	19.0500	-97.2660
S. grammicus	USNM	S/N / 9811	México	Puebla	19.2830	-98.4330
S. grammicus	USNM	S/N / 25502	México	San Luis Potosí	23.1500	-102.2000
S. grammicus	USNM	47711	México	Tamaulipas	26.4280	-99.1470
S. grammicus	USNM	299657	US	Texas	26.0660	-97.8750
S. grammicus	USNM	S/N / 52556	México	Veracruz	18.8000	-97.2660
S. grammicus	USNM	S/N / 32092	México	Veracruz	19.4160	-97.0660
S. grammicus	USNM	S/N / 37322	México	Veracruz	18.7660	-96.8160
S. grammicus	USNM	S/N / 33867	México	Veracruz	19.4160	-97.0000
S. grammicus	USNM	S/N / 38757	México	Zacatecas	23.2160	-102.8330
S. grammicus	ROM	13326	México	Michoacán	19.5158	-101.6094
S. grammicus	ROM	13327	México	Michoacán	19.5160	-101.6160
S. grammicus	ROM	13327	México	Michoacán	19.5167	-101.6167
S. grammicus	SDNHM	48986	México	Chihuahua	29.0550	-107.8517
S. grammicus	SDNHM	60477	México	Coahuila	26.3400	-101.3600
S. grammicus	SDNHM	48987	México	Coahuila	28.9780	-102.5518
S. grammicus	SDNHM	49776	México	Tamaulipas	24.6230	-99.0300
S. grammicus	TCWC	55374	México	Durango	24.0236	-105.1038
S. grammicus	TCWC	35704	México	México	19.1250	-98.6406

S. grammicus	TCWC	58508	México	México	19.2953	-98.7083
S. grammicus	TCWC	838	México	México	19.3583	-98.7042
S. grammicus	TCWC	12703	México	México	19.3500	-98.7333
S. grammicus	TCWC	54341	México	México	19.0653	-99.8444
S. grammicus	TCWC	52905	México	Michoacán	20.2540	-100.2520
S. grammicus	TCWC	52906-52915	México	Michoacán	20.2660	-100.2880
S. grammicus	TCWC	49520	México	Nuevo León	24.5920	-99.9950
S. grammicus	TCWC	58086	México	Nuevo León	24.6900	-100.1900
S. grammicus	TCWC	55333	México	Nuevo León	24.6790	-99.8450
S. grammicus	TCWC	57480	México	Nuevo León	24.8330	-99.5500
S. grammicus	TCWC	56689	México	Nuevo León	26.4830	-100.2500
S. grammicus	TCWC	6669	México	Puebla	19.0367	-98.6231
S. grammicus	TCWC	40786-40790	México	Querétaro	20.1420	-100.1300
S. grammicus	TCWC	29735	México	Querétaro	21.2860	-99.4440
S. grammicus	TCWC	55338-55339	México	Querétaro	20.7790	-99.7160
S. grammicus	TCWC	57260-57268	México	Querétaro	20.6980	-99.7520
S. grammicus	TCWC	29742	México	Querétaro	21.2870	-99.1170
S. grammicus	TCWC	32381	México	Querétaro	21.2920	-99.0620
S. grammicus	TCWC	35703	México	Querétaro	21.3110	-99.1970
S. grammicus	TCWC	37669	México	Querétaro	21.2800	-99.1220
S. grammicus	TCWC	45467	México	Querétaro	21.2910	-99.1160
S. grammicus	TCWC	54918	México	Querétaro	21.2910	-99.2070
S. grammicus	TCWC	55335	México	Querétaro	21.2820	-99.1420
S. grammicus	TCWC	57137	México	Querétaro	21.2600	-99.1730
S. grammicus	TCWC	29725-29733	México	Querétaro	21.2890	-99.2620
S. grammicus	TCWC	30677-30678	México	Querétaro	21.2920	-99.0690
S. grammicus	TCWC	32429-32442	México	Querétaro	21.2720	-99.2010
S. grammicus	TCWC	32451-32466	México	Querétaro	21.2850	-99.2510
S. grammicus	TCWC	36632-36633	México	Querétaro	21.2920	-99.1590
S. grammicus	TCWC	57134-57136	México	Querétaro	21.2910	-99.1090
S. grammicus	TCWC	32444	México	Querétaro	21.1310	-99.8010
S. grammicus	TCWC	32445	México	Querétaro	21.0900	-99.8180
S. grammicus	TCWC	40792-40794	México	Querétaro	20.9590	-99.7190
S. grammicus	TCWC	32450	México	Querétaro	21.1330	-99.6610
S. grammicus	TCWC	38385-38389	México	Querétaro	21.1440	-99.6480
S. grammicus	TCWC	57159-57162	México	Querétaro	21.2080	-99.5580
S. grammicus	TCWC	57163-57164	México	Querétaro	21.2010	-99.5650
S. grammicus	TCWC	40795-40797	México	Querétaro	20.9410	-99.5720
S. grammicus	TCWC	57165-57167	México	Querétaro	20.9170	-99.5580
S. grammicus	TCWC	61094	México	Querétaro	20.3870	-99.9950
S. grammicus	TCWC	57189	México	San Luis Potosí	23.0440	-100.4940
S. grammicus	TCWC	57276	México	San Luis Potosí	22.5970	-100.5760
S. grammicus	TCWC	56605 - 609	México	San Luis Potosí	22.6250	-100.5360
S. grammicus	TCWC	52918	México	Tamaulipas	23.2960	-99.6620
S. grammicus	TCWC	55352	México	Tamaulipas	23.2640	-99.6790
S. grammicus	TCWC	49522	México	Tamaulipas	24.5330	-99.5360
S. grammicus	TCWC	55377	México	Tamaulipas	24.0750	-99.1410
S. grammicus	TCWC	49515	México	Tamaulipas	24.6170	-98.9100
S. grammicus	TCWC	49518	México	Tamaulipas	24.6140	-98.9300
S. grammicus	TCWC	49521	México	Tamaulipas	24.4110	-98.8630
S. grammicus	TCWC	55279	México	Tamaulipas	24.6230	-99.0440
S. grammicus	TCWC	55367	México	Tamaulipas	24.3240	-98.8900
S. grammicus	TCWC	55368	México	Tamaulipas	24.3130	-98.8800
S. grammicus	TCWC	55375	México	Tamaulipas	24.2200	-99.1640
S. grammicus	TCWC	58088	México	Tamaulipas	24.6230	-99.0310
S. grammicus	TCWC	55334	México	Tamaulipas	24.7350	-99.3560
S. grammicus	TCWC	57315	México	Tamaulipas	23.1180	-98.7420
S. grammicus	TCWC	57383	México	Tamaulipas	23.0770	-98.9490
S. grammicus	TCWC	62236	US	Texas	26.3410	-98.7210
S. grammicus	UAZ	UAZ 46998	México	Coahuila	27.0330	-102.4500
S. grammicus	UAZ	UAZ 02522	México	Hidalgo	20.7370	-99.3820
S. grammicus	UAZ	UAZ 37936	México	Nuevo León	24.9330	-100.2500
S. grammicus	UCM	28740	México	México	19.3333	-98.6706
S. grammicus	IUMNH	24642	México	Oaxaca	17.4160	-96.2500
S. grammicus	IUMNH	43129-132	México	San Luis Potosí	22.1500	-100.9660
S. grammicus	IUMNH	42740-741	México	Veracruz	19.5830	-97.1000
S. grammicus	ND	99885	México	Querétaro	20.6940	-99.8150
S. grammicus	ND	143741	México	Querétaro	20.5000	-100.4150
S. grammicus	ND	128968	México	Querétaro	21.0880	-99.7080
S. grammicus	UMMZ	98999	México	Durango	24.1000	-105.5500
S. grammicus	UMMZ	104033	México	Hidalgo	20.1500	-98.2160
S. grammicus	UMMZ	94299-300	México	Michoacán	19.6660	-100.4160
S. grammicus	UMMZ	101997	México	Michoacán	19.8000	-100.6000
S. grammicus	UMMZ	104644	México	Michoacán	19.6500	-100.7830
S. grammicus	UMMZ	102000-001	México	Michoacán	19.6830	-100.8660

S. grammicus	UMMZ	94301	México	Michoacán	19.6160	-102.1660
S. grammicus	UMMZ	98997	México	Michoacán	19.5330	-101.7000
S. grammicus	UMMZ	104731	México	Michoacán	19.5330	-101.6660
S. grammicus	UMMZ	18990	México	Michoacán	19.4000	-101.5000
S. grammicus	UMMZ	99778	México	Michoacán	19.4330	-101.6000
S. grammicus	UMMZ	99779	México	Michoacán	19.3330	-101.6500
S. grammicus	UMMZ	101999	México	Michoacán	19.3160	-101.5500
S. grammicus	UMMZ	94298	México	Michoacán	19.4160	-102.3160
S. grammicus	UMMZ	104656	México	Michoacán	19.6160	-100.7830
S. grammicus	UMMZ	94297	México	Michoacán	19.5500	-102.0500
S. grammicus	UMMZ	88235	México	Nuevo León	24.9520	-100.2900
S. grammicus	UMMZ	105412 / 17556 / A014	México	Oaxaca	16.5660	-96.7330
S. grammicus	UMMZ	118802	México	Oaxaca	17.4160	-96.4330
S. grammicus	UMMZ	101509	México	Tamaulipas	23.1720	-99.3190
S. grammicus	UMMZ	102969	México	Tamaulipas	23.1720	-99.3090
S. grammicus	UMMZ	110753	México	Tamaulipas	23.2330	-99.3330
S. grammicus	UMMZ	110755	México	Tamaulipas	23.2330	-99.3660
S. grammicus	UMMZ	111218	México	Tamaulipas	23.1760	-99.3100
S. grammicus	UMMZ	111220	México	Tamaulipas	23.2150	-99.2640
S. grammicus	UMMZ	111222	México	Tamaulipas	23.0720	-99.2300
S. grammicus	UMMZ	111223	México	Tamaulipas	23.1090	-99.1220
S. grammicus	UTA	4581	México	México	19.0483	-99.3189
S. grammicus	UTA	7929	México	México	19.0475	-99.3194
S. grammicus	UTEP	9333	México	Durango	25.3700	-106.5300
S. grammicus	UTEP	9144	México	Querétaro	20.1370	-100.1140
S. grammicus	UTEP	6807	México	Zacatecas	24.4700	-101.3800
S. grammicus	YPM	HERR.007807	México	Puebla	19.4000	-97.6667
S. grammicus	YPM	YPM HERR 007807	México	Puebla	19.4000	-97.6670
S. heterolepis	CAS	169624	México	Jalisco	20.8000	-103.7300
S. heterolepis	CAS	3713	México	Jalisco	20.4400	-104.8200
S. heterolepis	CAS	144577	México	Jalisco	19.8900	-103.5200
S. heterolepis	CAS	100164	México	Jalisco	19.4700	-103.4600
S. heterolepis	ENCB	13508	México	Jalisco	19.5270	-103.6080
S. heterolepis	ENCB	13516	México	Jalisco	19.5270	-103.5480
S. heterolepis	ENCB	507	México	Jalisco	19.5270	-103.6380
S. heterolepis	MZFC	6768	México	Jalisco	19.4660	-103.9500
S. heterolepis	CNAR	7174	México	Jalisco	19.9200	-103.1200
S. heterolepis	CNAR	AR8876	México	Jalisco	19.9000	-103.0660
S. heterolepis	CNAR	AR8285	México	Jalisco	20.4200	-104.9800
S. heterolepis	CNAR	7171	México	Jalisco	19.5800	-103.6100
S. heterolepis	KU	27271	México	Jalisco	20.8000	-103.8500
S. heterolepis	KU	27271-273	México	Jalisco	20.7830	-103.8830
S. heterolepis	MCZ	R-32346	México	Jalisco	20.5330	-104.7830
S. heterolepis	MVZ	144196	México	Jalisco	19.5396	-103.5626
S. heterolepis	LACM	25777	México	Jalisco	19.4500	-103.5500
S. heterolepis	LACM	25778	México	Jalisco	19.5300	-103.6100
S. heterolepis	LACM	25786	México	Jalisco	19.4300	-103.4200
S. heterolepis	UCM	59252	México	Jalisco	20.7330	-103.5660
S. insignis	CNAR	5230	México	Jalisco	19.4300	-103.5000
S. insignis	CNAR	5751	México	Jalisco	19.8100	-104.1100
S. insignis	LACM	25736	México	Jalisco	19.4300	-103.5400
S. insignis	CAS	95895	México	Jalisco	20.5200	-104.1100
S. insignis	CAS	165286	México	Michoacán	18.7660	-102.9330
S. insignis	UMMZ	119102	México	Michoacán	18.8160	-102.9330
S. jarrovii	CNAR	7124	México	Sonora	29.7619	-110.3106
S. jarrovii	USNM	S/N / 45068	México	Sonora	27.4660	-108.6500
S. jarrovii	UMMZ	117793	México	Sonora	27.4330	-108.7660
S. jarrovii	CNAR	7128	México	Chihuahua	31.4100	-107.5700
S. jarrovii	CAS	34856	México	Sonora	30.2600	-108.8500
S. jarrovii	CAS	159194	México	Sinaloa	25.8600	-107.5900
S. jarrovii	CAS	159158	México	Sinaloa	25.9000	-107.6400
S. jarrovii	CAS	159192	México	Sinaloa	25.9100	-107.5200
S. jarrovii	CAS	159451	México	Sinaloa	25.9400	-107.5700
S. jarrovii	CAS	159173	México	Sinaloa	25.9500	-107.5600
S. jarrovii	UCM	61500	México	Chihuahua	26.5400	-106.4700
S. jarrovii	CAS	15161	México	Sonora	30.4833	-108.7667
S. jarrovii	KU	44189-205	México	Chihuahua	27.7500	-107.6330
S. jarrovii	KU	44185-187	México	Chihuahua	27.9000	-107.5660
S. jarrovii	KU	51817	México	Chihuahua	27.9330	-107.5830
S. jarrovii	LACM	17472	México	Chihuahua	27.7500	-107.4500
S. jarrovii	CAS	48399	México	Chihuahua	29.5152	-106.6954
S. jarrovii	CAS	34849	México	Chihuahua	29.5583	-106.8222
S. jarrovii	KU	47297-298	México	Chihuahua	29.7500	-107.5500
S. jarrovii	MVZ	68836	México	Chihuahua	29.4850	-106.7704
S. jarrovii	MVZ	66025	México	Chihuahua	29.5150	-106.6950

S. jarrovii	MVZ	92515	México	Chihuahua	29.5580	-106.8222
S. jarrovii	MVZ	84641	México	Chihuahua	29.5660	-106.6430
S. jarrovii	AMNH	84625-628	México	Sonora	30.9830	-110.4660
S. jarrovii	CNAR	7117	México	Sonora	30.9847	-110.3211
S. jarrovii	CNAR	7119	México	Sonora	31.0050	-110.4350
S. jarrovii	KU	50733	México	Sonora	31.0330	-110.2500
S. jarrovii	LACM	127771	México	Sonora	30.9747	-110.0194
S. jarrovii	LACM	95940	México	Sonora	31.0200	-110.3700
S. jarrovii	IUMNH	40475	México	Durango	24.3000	-104.7000
S. jarrovii	CNAR	7126	México	Durango	25.1200	-106.5400
S. jarrovii	CAS	48356	México	Chihuahua	30.0146	-108.4168
S. jarrovii	CAS	50493	México	Chihuahua	30.0830	-108.3500
S. jarrovii	FMNH	1658	México	Chihuahua	29.9660	-108.3330
S. jarrovii	MVZ	46666	México	Chihuahua	30.0140	-108.4160
S. jarrovii	LACM	75308	México	Chihuahua	30.0000	-108.3167
S. jarrovii	LACM	75309	México	Chihuahua	30.0333	-108.3333
S. jarrovii	USNM	26600	México	Chihuahua	29.9107	-108.3333
S. jarrovii	UCM	20905	México	Chihuahua	29.8100	-108.1800
S. jarrovii	CAS	34800	US	New Mexico	33.2973	-108.4594
S. jarrovii	MVZ	29297	US	New Mexico	33.2970	-108.4594
S. jarrovii	MZFC	5471	México	Zacatecas	23.5100	-103.8200
S. jarrovii	KU	51810-811	México	Chihuahua	29.5500	-106.2160
S. jarrovii	MVZ	70717	México	Chihuahua	29.3226	-106.4500
S. jarrovii	MVZ	65902	México	Chihuahua	29.3600	-106.3400
S. jarrovii	CAS	95896	US	Arizona	31.4280	-110.2890
S. jarrovii	CAS	91894	US	Arizona	31.4620	-110.2890
S. jarrovii	CAS	4942	US	Arizona	31.4630	-110.4230
S. jarrovii	CAS	48130	US	Arizona	31.4638	-110.4236
S. jarrovii	CAS	159186	US	Arizona	31.5000	-110.2500
S. jarrovii	CAS	48364	US	Arizona	31.7610	-109.2949
S. jarrovii	CAS	48280	US	Arizona	31.8830	-109.2030
S. jarrovii	CAS	48352	US	Arizona	31.8844	-109.1778
S. jarrovii	CAS	48335	US	Arizona	31.8923	-109.1681
S. jarrovii	CAS	686	US	Arizona	31.9123	-109.1848
S. jarrovii	CAS	229034	US	Arizona	31.9177	-109.2790
S. jarrovii	CAS	100102	US	Arizona	31.9297	-109.3817
S. jarrovii	CAS	48380	US	Arizona	32.0632	-110.0747
S. jarrovii	CUMV	13332	US	Arizona	31.4330	-110.3030
S. jarrovii	CUMV	9048	US	Arizona	31.4625	-110.2889
S. jarrovii	CUMV	10655	US	Arizona	31.5200	-109.1100
S. jarrovii	CUMV	5096	US	Arizona	31.8920	-109.1681
S. jarrovii	CUMV	13808	US	Arizona	31.9122	-109.1840
S. jarrovii	CUMV	5780	US	Arizona	31.9151	-109.2845
S. jarrovii	CUMV	8050	US	Arizona	31.9394	-109.9791
S. jarrovii	CUMV	8050	US	Arizona	32.0632	-110.0740
S. jarrovii	MVZ	79202	US	Arizona	31.4092	-110.2231
S. jarrovii	MVZ	225544	US	Arizona	31.4148	-110.3334
S. jarrovii	MVZ	44720	US	Arizona	31.4162	-110.2714
S. jarrovii	MVZ	214694	US	Arizona	31.4281	-110.2739
S. jarrovii	MVZ	65926	US	Arizona	31.4383	-110.3160
S. jarrovii	MVZ	49625	US	Arizona	31.4567	-110.2389
S. jarrovii	MVZ	67083	US	Arizona	31.7438	-109.3486
S. jarrovii	MVZ	225531	US	Arizona	31.7558	-109.3710
S. jarrovii	MVZ	137635	US	Arizona	31.7794	-109.3097
S. jarrovii	MVZ	137455	US	Arizona	31.8621	-109.1881
S. jarrovii	MVZ	67185	US	Arizona	31.8674	-109.3657
S. jarrovii	MVZ	137631	US	Arizona	31.8708	-109.2333
S. jarrovii	MVZ	96920	US	Arizona	31.8726	-109.1890
S. jarrovii	MVZ	7815	US	Arizona	31.8728	-109.2308
S. jarrovii	MVZ	67040	US	Arizona	31.8739	-109.1815
S. jarrovii	MVZ	67012	US	Arizona	31.8825	-109.2033
S. jarrovii	MVZ	67099	US	Arizona	31.8825	-109.2288
S. jarrovii	MVZ	67149	US	Arizona	31.8825	-109.2373
S. jarrovii	MVZ	67154	US	Arizona	31.8825	-109.2544
S. jarrovii	MVZ	67054	US	Arizona	31.8861	-109.1708
S. jarrovii	MVZ	67095	US	Arizona	31.8917	-109.1658
S. jarrovii	MVZ	67043	US	Arizona	31.8961	-109.2750
S. jarrovii	MVZ	215592	US	Arizona	31.9136	-109.1408
S. jarrovii	MVZ	225541	US	Arizona	31.9143	-109.2671
S. jarrovii	MVZ	7812	US	Arizona	31.9150	-109.2839
S. jarrovii	MVZ	66964	US	Arizona	31.9192	-109.9856
S. jarrovii	MVZ	80340	US	Arizona	31.9250	-109.9667
S. jarrovii	MVZ	7803	US	Arizona	31.9253	-109.2964
S. jarrovii	MVZ	7804	US	Arizona	31.9339	-109.3192
S. jarrovii	MVZ	18956	US	Arizona	31.9347	-109.3204

S. jarrovii	MVZ	7809	US	Arizona	31.9545	-109.3001
S. jarrovii	MVZ	66892	US	Arizona	31.9566	-109.2628
S. jarrovii	MVZ	7801	US	Arizona	31.9588	-109.3137
S. jarrovii	MVZ	18950	US	Arizona	32.0031	-109.3253
S. jarrovii	MVZ	42555	US	Arizona	32.0044	-109.3561
S. jarrovii	LACM	140842	US	Arizona	31.4330	-110.2830
S. jarrovii	LACM	140806	US	Arizona	31.9330	-109.9500
S. jarrovii	USNM	574025	US	Arizona	31.8844	-109.2194
S. jarrovii	SDNHM	49097	US	Arizona	31.4625	-110.2889
S. jarrovii	TCWC	76401	US	Arizona	31.4333	-110.3031
S. jarrovii	UCM	20493	US	Arizona	31.8901	-109.1686
S. jarrovii	YPM	YPM HERR 006752	US	Arizona	31.5000	-110.2500
S. jarrovii	YPM	YPM HERR 017543	US	Arizona	31.8844	-109.1778
S. jarrovii	CNAR	7122	México	Sinaloa	23.5617	-105.8383
S. jarrovii	MVZ	59092	México	Sinaloa	23.5690	-105.8496
S. jarrovii	MVZ	59091	México	Sinaloa	23.6280	-105.8425
S. jarrovii	MVZ	59090	México	Sinaloa	23.6300	-105.8415
S. jarrovii	LACM	129952	México	Sinaloa	23.5900	-105.8400
S. jarrovii	TCWC	76405	México	Sinaloa	23.6310	-105.8415
S. jarrovii	IUMNH	41625	México	Sinaloa	23.5660	-105.8500
S. jarrovii	UTA	17391	México	Sinaloa	23.6285	-105.8425
S. jarrovii	UTEP	14565	México	Sinaloa	23.5800	-105.8300
S. jarrovii	UCM	20918-922	México	Chihuahua	28.4000	-106.8330
S. jarrovii	UMMZ	118969	México	Chihuahua	28.4000	-107.0500
S. jarrovii	MZFC	6657	México	Durango	25.1660	-103.8660
S. jarrovii	FMNH	32032	México	Durango	24.9160	-103.8000
S. jarrovii	AMNH	68346	México	Durango	24.0330	-104.6500
S. jarrovii	CAS	169747	México	Durango	23.9100	-105.3400
S. jarrovii	CAS	114936	México	Durango	23.9900	-104.7500
S. jarrovii	MZFC	3188	México	Durango	23.9830	-104.7500
S. jarrovii	KU	44831-832	México	Durango	23.7660	-104.9160
S. jarrovii	ND	S/N / 4468	México	Durango	23.8500	-105.2660
S. jarrovii	USNM	S/N / 4296	México	Durango	24.1500	-104.5830
S. jarrovii	UCM	48908-910	México	Durango	23.8660	-105.1830
S. jarrovii	UCM	49631	México	Durango	24.0330	-104.6660
S. jarrovii	IUMNH	6572-578	México	Durango	24.1500	-105.0330
S. jarrovii	UTEP	7312	México	Durango	23.9167	-104.9667
S. jarrovii	USNM	S/N / 53234	México	Nayarit	22.5160	-104.6330
S. jarrovii	AMNH	68445	México	Chihuahua	29.2500	-107.7500
S. jarrovii	CNAR	2755	México	Chihuahua	29.3900	-107.6200
S. jarrovii	KU	47310-311	México	Chihuahua	29.2500	-107.7660
S. jarrovii	UMMZ	113648	México	Durango	25.7330	-103.6330
S. jarrovii	MVZ	226206	US	Arizona	32.6929	-109.9015
S. jarrovii	MVZ	226205	US	Arizona	32.7021	-109.9192
S. jarrovii	MVZ	226216	US	Arizona	32.8539	-110.0988
S. jarrovii	LACM	61857	US	Arizona	32.6321	-109.8150
S. jarrovii	LACM	145260	US	Arizona	32.7667	-110.0660
S. jarrovii	SDNHM	62707	US	Arizona	32.6513	-109.8567
S. jarrovii	SDNHM	62690	US	Arizona	32.8609	-110.1940
S. jarrovii	LACM	4472	US	New Mexico	32.6861	-108.7375
S. jarrovii	CAS	35031	México	Chihuahua	26.8216	-107.0805
S. jarrovii	MZFC	5759	México	Chihuahua	27.3000	-107.2160
S. jarrovii	KU	51072-075	México	Chihuahua	27.3000	-107.2330
S. jarrovii	KU	44206	México	Chihuahua	27.5160	-107.4830
S. jarrovii	MVZ	24324	México	Chihuahua	26.8210	-107.0805
S. jarrovii	LACM	17470	México	Chihuahua	27.4600	-107.4900
S. jarrovii	USNM	S/N / 2081	México	Chihuahua	26.8160	-107.0660
S. jarrovii	UCM	61196	México	Chihuahua	27.4100	-107.3500
S. jarrovii	UCM	61198	México	Chihuahua	27.4100	-107.4200
S. jarrovii	LACM	95928	México	Durango	24.5400	-104.0700
S. jarrovii	MZFC	935	México	Chihuahua	26.5100	-107.5000
S. jarrovii	USNM	S/N / 44776	México	Chihuahua	26.1000	-106.9660
S. jarrovii	CNAR	4855	México	Durango	26.3800	-106.4200
S. jarrovii	KU	51813	México	Chihuahua	27.3000	-108.2160
S. jarrovii	CNAR	2753	México	Chihuahua	28.3800	-107.6600
S. jarrovii	KU	51798	México	Chihuahua	28.1500	-107.4830
S. jarrovii	KU	51799-809	México	Chihuahua	28.4500	-107.4500
S. jarrovii	MVZ	59048	México	Chihuahua	28.4110	-107.1300
S. jarrovii	LACM	17443	México	Chihuahua	28.4110	-107.1080
S. jarrovii	YPM	HERR.017422	México	Chihuahua	28.4111	-107.1308
S. jarrovii	CAS	661	US	New Mexico	31.3899	-108.4603
S. jarrovii	CAS	100095	US	New Mexico	31.4090	-108.5376
S. jarrovii	CAS	91889	US	New Mexico	31.4092	-108.5376
S. jarrovii	CAS	48143	US	New Mexico	31.4375	-108.9051
S. jarrovii	CAS	100097	US	New Mexico	31.4375	-108.9051

S. jarrovii	CAS	48236	US	New Mexico	31.4500	-108.2300
S. jarrovii	CAS	159459	US	New Mexico	31.4617	-109.0362
S. jarrovii	CAS	35025	US	New Mexico	31.5100	-108.7100
S. jarrovii	CAS	48183	US	New Mexico	31.5175	-108.9780
S. jarrovii	CAS	100100	US	New Mexico	31.5175	-108.9780
S. jarrovii	CAS	204127	US	New Mexico	31.5336	-108.8767
S. jarrovii	CAS	48234	US	New Mexico	31.5653	-108.7828
S. jarrovii	CAS	34851	US	New Mexico	31.5700	-108.6400
S. jarrovii	CAS	34811	US	New Mexico	31.5811	-108.7836
S. jarrovii	CAS	2404	US	New Mexico	31.5900	-109.0300
S. jarrovii	CAS	34875	US	New Mexico	31.5900	-108.7700
S. jarrovii	CAS	665	US	New Mexico	31.6375	-108.3957
S. jarrovii	CAS	35034	US	New Mexico	32.0000	-109.0000
S. jarrovii	CUMV	13767	US	New Mexico	31.7290	-108.9780
S. jarrovii	CNAR	7123	México	Durango	25.7400	-104.8600
S. jarrovii	MSB	50377	US	New Mexico	31.5000	-108.7600
S. jarrovii	MSB	41200	US	New Mexico	31.5200	-109.0000
S. jarrovii	MSB	49138	US	New Mexico	31.5200	-108.9900
S. jarrovii	MSB	4010	US	New Mexico	31.5700	-108.7800
S. jarrovii	MSB	63078	US	New Mexico	31.5800	-108.6500
S. jarrovii	MSB	39270	US	New Mexico	31.6700	-108.6800
S. jarrovii	MSB	12108	US	New Mexico	31.7800	-108.9000
S. jarrovii	MSB	6314	US	New Mexico	32.3600	-109.0200
S. jarrovii	MSUM	HE.4334	US	New Mexico	31.4900	-108.7500
S. jarrovii	MVZ	59050	US	New Mexico	31.5180	-109.0020
S. jarrovii	MVZ	225535	US	New Mexico	31.5556	-108.9717
S. jarrovii	MVZ	98805	US	New Mexico	31.5650	-108.7828
S. jarrovii	MVZ	225532	US	New Mexico	31.5810	-108.7836
S. jarrovii	MVZ	225537	US	New Mexico	31.6370	-108.3950
S. jarrovii	MVZ	67182	US	New Mexico	31.7480	-108.9850
S. jarrovii	LACM	4471	US	New Mexico	31.3890	-108.4603
S. jarrovii	LACM	4468	US	New Mexico	31.4617	-109.0362
S. jarrovii	LACM	126640	US	New Mexico	31.5108	-109.0418
S. jarrovii	LACM	4467	US	New Mexico	31.5320	-108.8767
S. jarrovii	LACM	133291	US	New Mexico	32.0030	-108.7678
S. jarrovii	LACM	123361	US	New Mexico	32.3644	-109.0236
S. jarrovii	SNOMNH	40834	US	New Mexico	31.8200	-108.9700
S. jarrovii	UCM	50037	México	Durango	25.7330	-104.8500
S. jarrovii	UCM	61201	US	New Mexico	31.5700	-108.6700
S. jarrovii	IUMNH	41238	US	New Mexico	31.7485	-108.9859
S. jarrovii	YPM	HERR.007130	US	New Mexico	31.4500	-108.3400
S. jarrovii	YPM	YPM HERR 002840	US	New Mexico	31.5056	-108.7103
S. jarrovii	MZFC	3205	México	Chihuahua	26.9200	-105.6300
S. jarrovii	CNAR	2754	México	Chihuahua	29.6700	-107.7100
S. jarrovii	LACM	135399	México	Durango	25.6000	-105.0100
S. jarrovii	UCM	50036	México	Durango	25.7330	-104.9160
S. jarrovii	MSB	60266	México	Chihuahua	31.3400	-108.7400
S. jarrovii	USNM	S/N / 45880	México	Chihuahua	31.2000	-108.8000
S. jarrovii	MZFC	6658	México	Durango	25.2660	-103.7660
S. jarrovii	FMNH	100174-178	México	Durango	25.2000	-103.7000
S. jarrovii	FMNH	32033-036	México	Durango	25.2330	-103.6160
S. jarrovii	USNM	S/N / 4395	México	Durango	25.3160	-103.8000
S. jarrovii	USNM	S/N / 4329	México	Durango	25.5000	-103.6000
S. jarrovii	UTEP	3720	México	Durango	25.2830	-103.6160
S. jarrovii	CUMV	844	México	Chihuahua	29.1400	-107.9400
S. jarrovii	CNAR	7120	México	Chihuahua	29.1800	-108.0200
S. jarrovii	IUMNH	21291-293	México	Chihuahua	27.8660	-107.9330
S. jarrovii	MZFC	3182	México	Durango	25.7500	-103.8000
S. jarrovii	CNAR	7121	México	Durango	25.9100	-103.6300
S. jarrovii	CNAR	1698	México	Durango	26.5900	-104.0600
S. jarrovii	ENCB	9739	México	Durango	23.3870	-104.2510
S. jarrovii	ENCB	9515	México	Durango	23.4140	-104.2900
S. jarrovii	ENCB	9516	México	Durango	23.4190	-104.2610
S. jarrovii	IUMNH	21290	México	Sonora	29.6830	-109.6500
S. jarrovii	MZFC	15104	México	Chihuahua	28.2100	-108.5600
S. jarrovii	LACM	17471	México	Chihuahua	28.0500	-108.4700
S. jarrovii	UMMZ	78390-396	México	Sonora	30.5830	-109.2160
S. jarrovii	CAS	48228	México	Chihuahua	29.3921	-106.8987
S. jarrovii	FMNH	126582	México	Chihuahua	29.4132	-106.8915
S. jarrovii	MVZ	84640	México	Chihuahua	29.3670	-106.8270
S. jarrovii	MVZ	72963	México	Chihuahua	29.3920	-106.8980
S. jarrovii	MVZ	92518	México	Chihuahua	29.4130	-106.8910
S. jarrovii	UCM	20941	México	Chihuahua	29.4235	-106.8812
S. jarrovii	ENCB	9777	México	Durango	25.2250	-104.3130
S. jarrovii	CAS	91884	México	Durango	23.8500	-104.2500

S. jarrovii	ENCB	10000	México	Durango	24.0240	-104.3250
S. jarrovii	MZFC	5751	México	Chihuahua	28.2160	-108.2500
S. jarrovii	MVZ	59087	México	Durango	26.6260	-105.9340
S. jarrovii	IUMNH	5951	México	Durango	26.6263	-105.9348
S. jarrovii	LACM	139100	US	Arizona	31.9500	-111.5800
S. jarrovii	UCM	12067	US	Arizona	31.7498	-111.0260
S. jarrovii	CAS	169718	México	Durango	23.6400	-105.8100
S. jarrovii	CAS	114901	México	Durango	23.6400	-105.8200
S. jarrovii	CAS	114898	México	Durango	23.6600	-105.7600
S. jarrovii	CAS	169795	México	Durango	23.7500	-105.5400
S. jarrovii	CAS	91880	México	Durango	23.7667	-105.4167
S. jarrovii	CAS	91851	México	Durango	23.7833	-105.3167
S. jarrovii	CAS	169780	México	Durango	23.7900	-105.3740
S. jarrovii	CAS	34807	México	Durango	23.7906	-105.3742
S. jarrovii	FMNH	1511	México	Durango	23.8160	-105.3330
S. jarrovii	FWMSH	S/N / 4467	México	Durango	23.7500	-105.4330
S. jarrovii	CNAR	4863	México	Durango	23.7200	-105.4800
S. jarrovii	CNAR	2799	México	Durango	23.7600	-105.4400
S. jarrovii	CNAR	4857	México	Durango	23.7600	-105.4200
S. jarrovii	CNAR	7130	México	Durango	23.7600	-105.4300
S. jarrovii	CNAR	4870	México	Durango	23.8200	-105.3400
S. jarrovii	KU	44820-830	México	Durango	23.6330	-105.4660
S. jarrovii	KU	44869-871	México	Durango	23.6660	-105.4330
S. jarrovii	MVZ	67393	México	Durango	23.7880	-105.3700
S. jarrovii	MVZ	67400	México	Durango	23.8150	-105.4018
S. jarrovii	LACM	95934	México	Durango	23.7000	-105.7200
S. jarrovii	LACM	50879	México	Durango	23.7100	-105.7100
S. jarrovii	UCM	41356	México	Durango	23.6500	-105.7500
S. jarrovii	UCM	20925-935	México	Durango	23.7330	-105.5500
S. jarrovii	UCM	20923-924	México	Durango	23.7830	-105.3500
S. jarrovii	UCM	12072	México	Durango	23.7886	-105.3706
S. jarrovii	IUMNH	41623-624	México	Durango	23.7660	-105.4160
S. jarrovii	IUMNH	43289-292	México	Durango	23.8500	-105.3160
S. jarrovii	UMMZ	118571	México	Durango	23.7830	-105.3660
S. jarrovii	UTA	17387	México	Durango	23.8156	-105.4018
S. jarrovii	CNAR	5202	México	Sinaloa	23.0594	-105.4917
S. jarrovii	LACM	50831	México	Durango	24.1000	-105.5900
S. jarrovii	UMMZ	102575	México	Durango	24.0500	-105.5830
S. jarrovii	UMMZ	113647	México	Durango	24.3000	-105.5160
S. jarrovii	AMNH	67720	México	Chihuahua	26.8830	-105.9330
S. jarrovii	CNAR	3526	México	Durango	24.9800	-104.4700
S. jarrovii	AMNH	67721-722	México	Chihuahua	26.7830	-105.8500
S. jarrovii	AMNH	68234	México	Chihuahua	26.8000	-105.8160
S. jarrovii	AMNH	67913-915	México	Chihuahua	26.8160	-105.7160
S. jarrovii	CAS	12694	US	Arizona	31.4660	-110.7070
S. jarrovii	CAS	92123	US	Arizona	31.4666	-110.7073
S. jarrovii	CAS	34784	US	Arizona	31.5529	-111.0328
S. jarrovii	CAS	48298	US	Arizona	31.7000	-110.8470
S. jarrovii	CAS	100097	US	Arizona	31.7001	-110.8507
S. jarrovii	CUMV	5094	US	Arizona	31.7247	-110.8800
S. jarrovii	MVZ	225536	US	Arizona	31.4139	-110.7326
S. jarrovii	MVZ	225548	US	Arizona	31.4152	-110.7369
S. jarrovii	MVZ	225547	US	Arizona	31.7010	-110.8123
S. jarrovii	MVZ	80335	US	Arizona	31.7202	-110.8077
S. jarrovii	MVZ	225543	US	Arizona	31.7214	-110.8625
S. jarrovii	MVZ	79588	US	Arizona	31.7250	-110.8794
S. jarrovii	LACM	146724	US	Arizona	31.6730	-110.9370
S. jarrovii	LACM	17494	US	Arizona	31.7130	-110.8790
S. jarrovii	LACM	4489	US	Arizona	31.7240	-110.8800
S. jarrovii	SDNHM	65178	US	Arizona	31.7131	-110.8798
S. jarrovii	IUMNH	31357	US	Arizona	31.7001	-110.8475
S. jarrovii	YPM	YPM HERR 007497	US	Arizona	31.3397	-110.4911
S. jarrovii	ENCB	912	México	Durango	25.3220	-105.2560
S. jarrovii	UCM	20945	México	Durango	25.0330	-105.4160
S. jarrovii	UCM	20936-944	México	Durango	25.0830	-105.6000
S. jarrovii	CAS	159174	México	Sinaloa	25.9100	-107.6500
S. jarrovii	LACM	95896	México	Zacatecas	23.5500	-103.6500
S. jarrovii	ENCB	9972	México	Durango	23.4130	-104.2050
S. jarrovii	CNAR	1795	México	Durango	23.4000	-104.2400
S. jarrovii	MVZ	65999	México	Chihuahua	28.5681	-108.1481
S. jarrovii	SDNHM	48991	México	Chihuahua	29.0500	-107.7600
S. jarrovii	UCM	20910	México	Chihuahua	28.8000	-107.8500
S. jarrovii	LACM	17469	México	Chihuahua	27.2700	-107.6900
S. jarrovii	UMMZ	111512	México	Chihuahua	27.5160	-107.8500
S. jarrovii	KU	44188	México	Chihuahua	27.8330	-108.5660

S. jarrovii	LACM	95935	México	Sonora	29.0200	-109.7000
S. lineolateralis	UCM	61194	México	Durango	25.9000	-103.4600
S. lineolateralis	CNAR	AR3137	México	Durango	24.9800	-104.4700
S. lineolateralis	MCZ	R-157778	México	Durango	25.2250	-104.3130
S. lineolateralis	AMNH	85273	México	Durango	25.7400	-104.8600
S. macdougalli	IUMNH	37333-337	México	Oaxaca	16.1830	-95.3330
S. melanogaster	AMNH	18474-475	México	Jalisco	20.2660	-102.6830
S. melanogaster	AMNH	18429	México	Jalisco	20.2830	-102.7160
S. melanogaster	AMNH	17983	México	Jalisco	20.4830	-103.2660
S. melanogaster	AMNH	82160-161	México	Zacatecas	23.3500	-102.9830
S. melanogaster	AMNH	68389	México	Zacatecas	22.7500	-102.5160
S. melanogaster	CAS	19486	México	Aguascalientes	22.2000	-102.0200
S. melanogaster	CAS	19501	México	Aguascalientes	22.1400	-102.2700
S. melanogaster	CAS	92100	México	Zacatecas	23.3800	-103.0100
S. melanogaster	CAS	96060	México	Zacatecas	22.6500	-102.8900
S. melanogaster	CAS	92105	México	Zacatecas	23.7300	-103.7600
S. melanogaster	CAS	92096	México	Zacatecas	22.4100	-102.8700
S. melanogaster	CM	73277	México	Querétaro	21.2950	-99.2560
S. melanogaster	CM	59731	México	Zacatecas	24.6900	-101.4500
S. melanogaster	MZFC	5517	México	Guanajuato	20.8330	-100.7660
S. melanogaster	MZFC	5035	México	Guanajuato	21.0800	-101.1800
S. melanogaster	MZFC	6900	México	Guanajuato	21.3000	-100.0500
S. melanogaster	MZFC	6105	México	Jalisco	19.9160	-103.0500
S. melanogaster	MZFC	3731	México	Jalisco	20.3500	-102.7660
S. melanogaster	MZFC	6792	México	Jalisco	20.8000	-103.8500
S. melanogaster	MZFC	3162	México	Zacatecas	23.0000	-103.2160
S. melanogaster	MZFC	3161	México	Zacatecas	22.8330	-103.6660
S. melanogaster	FMNH	32108	México	Aguascalientes	21.6830	-102.3000
S. melanogaster	FMNH	32071-083	México	Guanajuato	21.0330	-100.7160
S. melanogaster	FMNH	32063	México	Guanajuato	21.1000	-101.1660
S. melanogaster	FMNH	32059	México	Guanajuato	20.1330	-100.5500
S. melanogaster	FMNH	106547-548	México	Guanajuato	21.4830	-101.2160
S. melanogaster	FMNH	32109	México	Jalisco	21.8000	-101.7160
S. melanogaster	FMNH	32120-121	México	Michoacán	19.8500	-100.8330
S. melanogaster	FMNH	32064-066	México	Zacatecas	24.1500	-101.4830
S. melanogaster	FWMSH	S/N / 7510	México	Jalisco	20.3000	-103.2830
S. melanogaster	FWMSH	S/N / 8483	México	Jalisco	21.5830	-101.6660
S. melanogaster	CNAR	3447	México	Aguascalientes	21.8567	-102.6517
S. melanogaster	CNAR	3446	México	Aguascalientes	22.1217	-102.3583
S. melanogaster	CNAR	4491	México	Aguascalientes	22.2100	-102.5300
S. melanogaster	CNAR	6129	México	Aguascalientes	22.1600	-102.5600
S. melanogaster	CNAR	6130	México	Aguascalientes	22.1800	-102.4200
S. melanogaster	CNAR	4488	México	Coahuila	24.8000	-101.2900
S. melanogaster	CNAR	5022	México	Guanajuato	21.3200	-100.2100
S. melanogaster	CNAR	6092	México	Jalisco	20.8100	-102.3600
S. melanogaster	CNAR	AR2928	México	Jalisco	20.7000	-102.3160
S. melanogaster	CNAR	4260	México	Jalisco	20.2300	-103.4200
S. melanogaster	CNAR	2744	México	Jalisco	20.2900	-103.2900
S. melanogaster	CNAR	2762	México	Jalisco	20.2900	-103.1900
S. melanogaster	CNAR	4182	México	Jalisco	20.1600	-102.8700
S. melanogaster	CNAR	6076	México	Jalisco	21.6200	-101.9300
S. melanogaster	CNAR	6077	México	Jalisco	21.5500	-101.9600
S. melanogaster	CNAR	6079	México	Jalisco	21.7400	-101.9800
S. melanogaster	CNAR	6075	México	Jalisco	21.9000	-101.6700
S. melanogaster	CNAR	4013	México	Jalisco	21.1600	-101.8500
S. melanogaster	CNAR	11070	México	Jalisco	21.7200	-102.6400
S. melanogaster	CNAR	11071	México	Jalisco	21.6900	-102.6600
S. melanogaster	CNAR	11073	México	Jalisco	21.7300	-102.6500
S. melanogaster	CNAR	11074	México	Jalisco	21.6900	-102.6000
S. melanogaster	CNAR	11076	México	Jalisco	21.7200	-102.6500
S. melanogaster	CNAR	11078	México	Jalisco	21.7100	-102.7100
S. melanogaster	CNAR	AR2939	México	Jalisco	21.6830	-102.5830
S. melanogaster	CNAR	6090	México	Jalisco	21.2100	-102.8900
S. melanogaster	CNAR	6091	México	Jalisco	21.1800	-102.9000
S. melanogaster	CNAR	3336	México	Michoacán	20.1167	-102.8500
S. melanogaster	CNAR	3337	México	Michoacán	20.1200	-102.8800
S. melanogaster	CNAR	4179	México	Michoacán	20.1000	-102.8100
S. melanogaster	CNAR	3458	México	Michoacán	19.8900	-101.1900
S. melanogaster	CNAR	7681	México	Michoacán	20.1900	-100.3400
S. melanogaster	CNAR	AR2932	México	Michoacán	20.1190	-100.2050
S. melanogaster	CNAR	2365	México	Michoacán	19.9800	-101.2500
S. melanogaster	CNAR	5220	México	Michoacán	20.0500	-102.9000
S. melanogaster	CNAR	5214	México	Michoacán	20.2400	-102.1200
S. melanogaster	CNAR	7679	México	Querétaro	20.1100	-100.1200
S. melanogaster	CNAR	4050	México	Querétaro	21.2100	-99.5200

S. melanogaster	CNAR	AR2890	México	Querétaro	21.2320	-99.5160
S. melanogaster	CNAR	4051	México	Querétaro	21.1300	-99.6200
S. melanogaster	CNAR	3705	México	Querétaro	20.9100	-99.5600
S. melanogaster	CNAR	4745	México	San Luis Potosí	23.2400	-100.9500
S. melanogaster	CNAR	3630	México	San Luis Potosí	23.1700	-102.2100
S. melanogaster	CNAR	4270	México	Zacatecas	23.1600	-102.9900
S. melanogaster	CNAR	4832	México	Zacatecas	23.1600	-103.0000
S. melanogaster	CNAR	4935	México	Zacatecas	23.1600	-103.0100
S. melanogaster	CNAR	4497	México	Zacatecas	24.1700	-103.2300
S. melanogaster	CNAR	5054	México	Zacatecas	22.2700	-101.9500
S. melanogaster	CNAR	4510	México	Zacatecas	22.3400	-102.1500
S. melanogaster	CNAR	11214	México	Zacatecas	22.3300	-101.6100
S. melanogaster	CNAR	4489	México	Zacatecas	23.5900	-103.1000
S. melanogaster	CNAR	4485	México	Zacatecas	21.4830	-103.4830
S. melanogaster	CNAR	4027	México	Zacatecas	22.8700	-102.5400
S. melanogaster	CNAR	5058	México	Zacatecas	22.6500	-102.7400
S. melanogaster	KU	29541	México	Zacatecas	22.7160	-102.3330
S. melanogaster	MCZ	R-133844	México	Michoacán	19.9600	-102.4000
S. melanogaster	MCZ	6807	México	San Luis Potosí	22.8660	-100.1500
S. melanogaster	MCZ	4548	México	San Luis Potosí	22.1500	-100.9660
S. melanogaster	MCZ	R-136370	México	San Luis Potosí	22.1200	-100.4600
S. melanogaster	USNM	247753	México	Jalisco	20.8160	-102.7160
S. melanogaster	USNM	S/N / 8933	México	Jalisco	20.6160	-103.0660
S. melanogaster	TCWC	6925	México	Hidalgo	20.7370	-99.3820
S. melanogaster	TCWC	12561	México	Michoacán	19.9660	-102.4330
S. melanogaster	UCM	16701-706	México	Michoacán	20.0000	-102.8830
S. melanogaster	UCM	16707-717	México	Michoacán	20.0000	-103.0160
S. melanogaster	IUMNH	6628	México	Guanajuato	21.2000	-101.7660
S. melanogaster	IUMNH	21613	México	Jalisco	20.2830	-103.1830
S. melanogaster	IUMNH	28160	México	Jalisco	21.7500	-101.8500
S. melanogaster	IUMNH	56105-110	México	Jalisco	21.9830	-101.7330
S. melanogaster	IUMNH	47906-923	México	Jalisco	20.5660	-103.8500
S. melanogaster	IUMNH	1527	México	Jalisco	20.4660	-102.9330
S. melanogaster	IUMNH	21615	México	Michoacán	19.9330	-100.4830
S. melanogaster	IUMNH	21611-612	México	San Luis Potosí	23.2000	-101.1330
S. melanogaster	IUMNH	6603-607	México	San Luis Potosí	22.1500	-101.0660
S. melanogaster	IUMNH	28159	México	Zacatecas	22.7160	-102.5160
S. melanogaster	IUMNH	43305	México	Zacatecas	23.5500	-103.2330
S. melanogaster	IUMNH	6630-631	México	Zacatecas	23.4830	-103.1000
S. melanogaster	IUMNH	21601-603	México	Zacatecas	23.8000	-102.4660
S. melanogaster	UMMZ	77267-271	México	San Luis Potosí	23.1330	-101.1160
S. minor	ENCB	15180	México	Aguascalientes	21.9440	-102.0860
S. minor	ND	S/N / 6962	México	Hidalgo	20.4330	-99.4500
S. minor	MVZ	186494	México	San Luis Potosí	22.1614	-100.6383
S. minor	TCWC	37681	México	Querétaro	21.5610	-99.6880
S. minor	AMNH	70697-699	México	Querétaro	20.7000	-99.8160
S. minor	MZFC	897	México	Querétaro	20.8160	-99.7330
S. minor	MZFC	6221	México	Querétaro	20.6830	-99.5660
S. minor	MZFC	6956	México	Querétaro	20.8000	-99.5830
S. minor	MZFC	7491	México	Querétaro	20.6810	-99.5560
S. minor	MZFC	8419	México	Querétaro	20.8780	-99.6120
S. minor	MZFC	9223	México	Querétaro	20.6840	-99.8150
S. minor	MZFC	9236	México	Querétaro	20.6710	-99.8070
S. minor	MZFC	9768	México	Querétaro	20.8520	-99.5830
S. minor	CNAR	3712	México	Querétaro	20.8800	-99.5900
S. minor	CNAR	4052	México	Querétaro	20.8300	-99.7200
S. minor	ND	99807	México	Querétaro	20.6940	-99.8150
S. minor	TCWC	35731	México	Querétaro	20.7330	-99.8020
S. minor	TCWC	35732	México	Querétaro	20.7000	-99.7000
S. minor	TCWC	40829	México	Querétaro	20.9340	-99.6140
S. minor	TCWC	32493-32509	México	Querétaro	20.6840	-99.8140
S. minor	TCWC	52434-52435	México	Querétaro	20.9500	-99.6760
S. minor	ENCB	10781	México	San Luis Potosí	23.8100	-100.7140
S. minor	ENCB	11266	México	Hidalgo	20.2210	-99.4690
S. minor	ENCB	14269	México	San Luis Potosí	22.4830	-99.4890
S. minor	ENCB	14270	México	San Luis Potosí	22.3880	-99.4440
S. minor	ENCB	17402	México	San Luis Potosí	22.6510	-99.9780
S. minor	ENCB	48901-906	México	San Luis Potosí	22.5000	-99.4160
S. minor	ND	S/N / 25226	México	San Luis Potosí	22.4160	-99.7500
S. minor	LACM	131190	México	San Luis Potosí	22.4400	-99.5700
S. minor	MZFC	9665	México	Querétaro	20.9200	-100.1950
S. minor	ND	9130	México	Querétaro	20.8170	-100.1830
S. minor	MZFC	10700	México	Zacatecas	24.6100	-101.4200
S. minor	ENCB	12321	México	Hidalgo	20.7330	-98.9170
S. minor	UMMZ	111195	México	Nuevo León	23.9330	-99.5160

S. minor	CNAR	4743	México	San Luis Potosí	22.6250	-100.5360
S. minor	CNAR	4744-4	México	San Luis Potosí	22.6420	-100.4320
S. minor	MVZ	146579-80	México	San Luis Potosí	22.8680	-100.2780
S. minor	ND	No DPhScja100	México	San Luis Potosí	22.6530	-100.3470
S. minor	ND	No DPhScja104	México	San Luis Potosí	22.6310	-100.1640
S. minor	ND	No DPhScja134	México	San Luis Potosí	23.0420	-100.4170
S. minor	ND	No DPhScja136	México	San Luis Potosí	22.6880	-100.5050
S. minor	ND	No DPhScja137	México	San Luis Potosí	22.6490	-100.4060
S. minor	ND	No DPhScja14	México	San Luis Potosí	22.6690	-100.4140
S. minor	ND	No DPhScja482	México	San Luis Potosí	22.7540	-100.2970
S. minor	ND	No DPhScja484	México	San Luis Potosí	22.7020	-100.3860
S. minor	ND	No DPhScja80	México	San Luis Potosí	22.6800	-100.4140
S. minor	ND	No DPhScja817	México	San Luis Potosí	22.7210	-100.3840
S. minor	ND	No DPhScja822	México	San Luis Potosí	22.6150	-100.3870
S. minor	ND	No DPhScja86	México	San Luis Potosí	22.6220	-100.4250
S. minor	ND	No DPhScja9	México	San Luis Potosí	22.6320	-100.3840
S. minor	ND	No DPhScja91	México	San Luis Potosí	22.6250	-100.3930
S. minor	ND	No DPhScja92	México	San Luis Potosí	22.6440	-100.4420
S. minor	TCWC	56801	México	San Luis Potosí	23.0420	-100.4910
S. minor	UTEP	UTEP 6081	México	San Luis Potosí	22.9080	-100.4080
S. minor	FMNH	65408-410	México	Hidalgo	20.2160	-98.5660
S. minor	CNAR	7561	México	Veracruz	20.4400	-98.5700
S. minor	CNAR	AR3620	México	Veracruz	20.4400	-98.5700
S. minor	MZFC	10735	México	Hidalgo	20.3700	-99.5400
S. minor	ND	N1	México	Hidalgo	20.5000	-99.1500
S. minor	AMNH	67422-423	México	Hidalgo	21.0160	-99.1330
S. minor	CM	147625	México	Hidalgo	20.9700	-99.1600
S. minor	ENCB	1067	México	Hidalgo	21.0050	-99.1200
S. minor	MZFC	5355	México	Hidalgo	21.0330	-99.1660
S. minor	MZFC	10659	México	Hidalgo	21.0000	-99.1200
S. minor	MVZ	129270	México	Hidalgo	21.0247	-99.1325
S. minor	USNM	S/N / 7004	México	Hidalgo	21.0660	-99.1660
S. minor	IUMNH	8715-716	México	Hidalgo	20.9500	-99.2000
S. minor	MZFC	6267	México	Querétaro	21.1430	-99.5030
S. minor	MZFC	8963	México	Querétaro	21.3980	-99.2520
S. minor	MZFC	8970	México	Querétaro	21.4840	-99.1950
S. minor	MZFC	9326	México	Querétaro	21.4380	-99.2130
S. minor	CNAR	4053	México	Querétaro	21.2100	-99.5200
S. minor	CNAR	AR4169	México	Querétaro	21.2100	-99.5200
S. minor	UANL	4831	México	Tamaulipas	23.2800	-99.4400
S. minor	TAMU	52997	México	Tamaulipas	23.1700	-99.3900
S. minor	UMMZ	101380	México	Tamaulipas	23.2330	-99.3660
S. minor	UMMZ	111198	México	Tamaulipas	23.2330	-99.4000
S. minor	AMNH	75828-830	México	Hidalgo	21.0500	-99.1000
S. minor	CNAR	97	México	Hidalgo	21.1200	-99.1700
S. minor	ND	N2	México	Hidalgo	21.0300	-99.1000
S. minor	MZFC	9846	México	Querétaro	21.3770	-99.1800
S. minor	CNAR	7724	México	Querétaro	21.2900	-99.1200
S. minor	ND	129744	México	Querétaro	21.2900	-99.1300
S. minor	TCWC	35733	México	Querétaro	21.3970	-99.1970
S. minor	TCWC	35734	México	Querétaro	21.3300	-99.2050
S. minor	TCWC	32677-32687	México	Querétaro	21.2920	-99.1370
S. minor	CNAR	5055	México	Zacatecas	22.2700	-101.9500
S. minor	CNAR	AR2213	México	Zacatecas	22.2700	-101.9500
S. minor	ENCB	6144	México	Hidalgo	20.3240	-98.3710
S. minor	ENCB	17379	México	Hidalgo	20.4930	-98.6910
S. minor	CNAR	AR3622	México	Hidalgo	20.4600	-98.7100
S. minor	ENCB	7212	México	San Luis Potosí	22.2670	-101.1120
S. minor	ENCB	14033	México	San Luis Potosí	22.3090	-101.2110
S. minor	MZFC	10731	México	Nuevo León	23.4100	-100.2600
S. minor	USNM	S/N / 30437	México	Nuevo León	23.5660	-99.9500
S. minor	ENCB	5736	México	Tamaulipas	23.5320	-99.7540
S. minor	ENCB	5751	México	Tamaulipas	23.7510	-99.8180
S. minor	ENCB	1068	México	Hidalgo	20.8360	-98.7460
S. minor	MZFC	5405	México	Hidalgo	20.7700	-99.1500
S. minor	LSUMNS	35031	México	Aguascalientes	22.1300	-102.2600
S. minor	MZFC	9189	México	Querétaro	21.1400	-99.6880
S. minor	ND	6091	México	Querétaro	21.0900	-99.8540
S. minor	TCWC	32473	México	Querétaro	21.1010	-99.8590
S. minor	TCWC	32484-32490	México	Querétaro	21.1010	-99.8830
S. minor	TCWC	32510-32534	México	Querétaro	21.0990	-99.8090
S. minor	TCWC	32535-32597	México	Querétaro	21.1010	-99.8670
S. minor	TCWC	32634-32638	México	Querétaro	21.0900	-99.7900
S. minor	TCWC	32639-32641	México	Querétaro	21.0900	-99.8180
S. minor	TCWC	40812-40815	México	Querétaro	20.9620	-99.7230

S. minor	TCWC	40816-40818	México	Querétaro	20.9590	-99.7190
S. minor	MZFC	813	México	Querétaro	21.1080	-99.6170
S. minor	MZFC	814	México	Querétaro	21.1120	-99.6240
S. minor	MZFC	6918	México	Querétaro	21.1420	-99.6210
S. minor	MZFC	6920	México	Querétaro	21.1350	-99.6250
S. minor	CNAR	4054	México	Querétaro	21.1300	-99.6200
S. minor	ND	S/N / 23865	México	Querétaro	21.1160	-99.6660
S. minor	USNM	S/N / 23852	México	Querétaro	21.1330	-99.6330
S. minor	TCWC	32628	México	Querétaro	21.1420	-99.5740
S. minor	TCWC	32629	México	Querétaro	21.1330	-99.6090
S. minor	TCWC	33067	México	Querétaro	21.1020	-99.6560
S. minor	TCWC	35730	México	Querétaro	21.1020	-99.6830
S. minor	TCWC	27774-27775	México	Querétaro	21.1330	-99.6610
S. minor	TCWC	32619-32627	México	Querétaro	21.1420	-99.5680
S. minor	TCWC	38390-38395	México	Querétaro	21.1440	-99.6480
S. minor	TCWC	45650-45651	México	Querétaro	21.1360	-99.6240
S. minor	ENCB	10717	México	Querétaro	20.8360	-100.5290
S. minor	CNAR	7116	México	San Luis Potosí	22.6400	-101.9400
S. minor	MZFC	6267	México	Hidalgo	20.6330	-98.6160
S. minor	ENCB	5199	México	Hidalgo	20.1230	-98.8870
S. minor	MZFC	5869	México	Querétaro	20.9100	-99.5600
S. minor	MZFC	9658	México	Querétaro	20.8910	-99.5180
S. minor	MZFC	9659	México	Querétaro	20.9280	-99.5550
S. minor	MZFC	9654-9655	México	Querétaro	20.9110	-99.5450
S. minor	MZFC	not recorded / 2568	México	Querétaro	20.9420	-99.5590
S. minor	CNAR	3711	México	Querétaro	20.9100	-99.5600
S. minor	TCWC	32472	México	Querétaro	20.9760	-99.4700
S. minor	TCWC	40823	México	Querétaro	20.8990	-99.5440
S. minor	TCWC	40824-40828	México	Querétaro	20.9410	-99.5720
S. minor	CNAR	6136	México	Aguascalientes	22.1800	-102.4200
S. minor	CNAR	6138	México	Aguascalientes	22.1500	-102.4200
S. minor	USNM	346557	México	Aguascalientes	22.2000	-102.5500
S. minor	ENCB	14129	México	San Luis Potosí	22.1220	-101.0370
S. minor	FWMSH	S/N / 25793	México	San Luis Potosí	22.0830	-101.1000
S. minor	IUMNH	6551-571	México	San Luis Potosí	22.1500	-101.0660
S. minor	CNAR	7723	México	San Luis Potosí	22.0600	-100.4700
S. minor	CNAR	AR9220	México	San Luis Potosí	22.0600	-100.4700
S. minor	MCZ	R-146583	México	San Luis Potosí	22.1200	-100.4600
S. minor	MZFC	6918	México	San Luis Potosí	21.6000	-99.3900
S. minor	CNAR	7721	México	San Luis Potosí	21.7800	-100.7200
S. minor	USNM	S/N / 25501	México	San Luis Potosí	23.1500	-102.2000
S. minor	ND	9856	México	Hidalgo	20.5100	-99.3400
S. minor	ENCB	10715	México	Querétaro	20.4830	-99.9220
S. minor	CNAR	3709	México	Querétaro	20.5310	-99.8880
S. minor	LACM	147683	México	Querétaro	20.5200	-99.9000
S. minor	MZFC	9404	México	Guanajuato	20.9480	-100.1790
S. minor	ENCB	4998	México	Hidalgo	19.9310	-98.8560
S. minor	MZFC	812	México	Querétaro	20.8620	-99.8110
S. minor	MZFC	6234	México	Querétaro	20.8210	-99.8440
S. minor	CAS	165257	México	Tamaulipas	22.9100	-99.5600
S. minor	MZFC	10733	México	San Luis Potosí	23.4200	-100.8000
S. minor	USNM	346559	México	Zacatecas	21.9830	-101.8830
S. minor	ENCB	14132 / 5127 / BC004	México	San Luis Potosí	22.0110	-100.5870
S. minor	CNAR	7722	México	San Luis Potosí	22.0700	-100.6300
S. minor	MVZ	186493	México	San Luis Potosí	22.0528	-100.6083
S. minor	MVZ	186495	México	San Luis Potosí	22.0772	-100.6353
S. minor	UMMZ	767692	México	San Luis Potosí	22.0330	-100.6000
S. minor	ENCB	12652	México	Hidalgo	20.8431	-99.2547
S. minor	CNAR	365	México	Hidalgo	20.8900	-99.2300
S. minor	IUMNH	8717	México	Hidalgo	20.8330	-99.2500
S. mucronatus	CAS	87322	México	Veracruz	19.6000	-97.0330
S. mucronatus	ENCB	6509	México	Veracruz	19.5990	-97.0220
S. mucronatus	MZFC	6844	México	Veracruz	19.6000	-97.0330
S. mucronatus	FMNH	112240	México	Veracruz	19.6160	-97.0330
S. mucronatus	CNAR	3827	México	Puebla	19.1500	-97.9300
S. mucronatus	MVZ	196095	México	Veracruz	19.6100	-97.0270
S. mucronatus	ND	S/N / 22108	México	Puebla	19.1330	-97.9330
S. mucronatus	IUMNH	25096	México	Puebla	19.0330	-97.9660
S. mucronatus	IUMNH	21355-360	México	Veracruz	19.6160	-97.0500
S. mucronatus	MZFC	7355	México	Hidalgo	20.2100	-98.2100
S. mucronatus	CNAR	3136	México	México	20.0928	-99.7883
S. mucronatus	CNAR	11978	México	México	20.1125	-99.7350
S. mucronatus	KU	67612	México	México	20.0719	-99.8500
S. mucronatus	ND	21370	México	México	20.0500	-99.7160
S. mucronatus	KU	27051	México	Hidalgo	20.3830	-98.3660

S. mucronatus	MCZ	10700-705	México	Hidalgo	20.1660	-99.0500
S. mucronatus	ENCB	10841	México	Puebla	19.1380	-97.5410
S. mucronatus	MCZ	R-129427	México	Puebla	19.1380	-97.5410
S. mucronatus	CNAR	2804	México	Distrito Federal	19.2500	-99.3130
S. mucronatus	CNAR	7672	México	Querétaro	20.1500	-100.1200
S. mucronatus	MZFC	3221	México	Puebla	19.0500	-98.0500
S. mucronatus	ENCB	13414	México	Puebla	19.7940	-97.9340
S. mucronatus	IUMNH	21361	México	Hidalgo	20.3660	-98.7330
S. mucronatus	UMMZ	99050	México	México	19.7500	-98.6500
S. mucronatus	UMMZ	99925	México	México	19.7500	-98.6660
S. mucronatus	ENCB	6310	México	Tlaxcala	19.5500	-98.5670
S. mucronatus	ENCB	13416	México	Puebla	18.9980	-97.4360
S. mucronatus	ND	No Aplica4	México	México	19.2692	-98.7100
S. mucronatus	ENCB	11651	México	Hidalgo	20.2310	-99.5140
S. mucronatus	MZFC	3461	México	Hidalgo	20.2330	-99.5500
S. mucronatus	ND	60892	México	Hidalgo	20.2330	-99.5660
S. mucronatus	MZFC	3259	México	Puebla	18.3330	-98.6660
S. mucronatus	MZFC	3260	México	Puebla	18.3330	-98.6500
S. mucronatus	MZFC	5725	México	Puebla	18.3330	-98.6000
S. mucronatus	MVZ	144164	México	San Luis Potosí	22.4371	-99.5800
S. mucronatus	FMNH	1516	México	Puebla	18.7830	-98.7160
S. mucronatus	ENCB	1586	México	Distrito Federal	19.2740	-99.3280
S. mucronatus	UTA	R-150194	México	Distrito Federal	19.3200	-99.3000
S. mucronatus	CNAR	8520	México	Hidalgo	19.9000	-98.3500
S. mucronatus	CNAR	AR2172	México	Hidalgo	19.9000	-98.3500
S. mucronatus	MZFC	3293	México	Hidalgo	20.2500	-98.8660
S. mucronatus	ND	112253	México	Hidalgo	20.2500	-98.8660
S. mucronatus	CNAR	AR2009	México	Tlaxcala	19.3300	-97.6800
S. mucronatus	UMMZ	118207	México	Hidalgo	20.0660	-98.6500
S. mucronatus	CNAR	AR5134	México	Michoacán	20.1190	-100.2050
S. mucronatus	CNAR	AR9696	México	Puebla	19.3700	-97.3900
S. mucronatus	ENCB	13425	México	Puebla	20.1610	-98.0660
S. mucronatus	MZFC	3232	México	Puebla	20.1160	-98.1000
S. mucronatus	MZFC	3351	México	Puebla	20.1660	-98.1000
S. mucronatus	CNAR	AR2029	México	Puebla	20.1600	-98.1500
S. mucronatus	MZFC	110	México	Hidalgo	20.4000	-99.5000
S. mucronatus	ND	S/N / 7180	México	Hidalgo	20.3330	-99.7330
S. mucronatus	UTEP	4581	México	Hidalgo	20.3700	-99.6500
S. mucronatus	UMMZ	101956	México	Veracruz	19.4160	-97.0660
S. mucronatus	CAS	143872	México	México	19.3330	-98.7060
S. mucronatus	MZFC	916	México	México	19.3361	-98.7194
S. mucronatus	MZFC	3205	México	México	19.3330	-98.7000
S. mucronatus	CNAR	3610	México	México	19.3317	-98.7236
S. mucronatus	CNAR	4330	México	México	19.3167	-98.7183
S. mucronatus	KU	37760	México	México	19.3472	-98.7550
S. mucronatus	KU	61659	México	México	19.3542	-98.7450
S. mucronatus	LSUMNS	21888	México	México	19.3500	-98.7111
S. mucronatus	ND	No Aplica5	México	México	19.3144	-98.7222
S. mucronatus	ND	No Aplica6	México	México	19.3222	-98.8150
S. mucronatus	ND	No Aplica7	México	México	19.3300	-98.7083
S. mucronatus	LACM	61983	México	México	19.3500	-98.7000
S. mucronatus	LACM	61983	México	México	19.3500	-98.7000
S. mucronatus	USNM	S/N / 9376	México	México	19.3160	-98.7330
S. mucronatus	USNM	S/N / 9648	México	México	19.3160	-98.7500
S. mucronatus	IUMNH	21362-363	México	México	19.3500	-98.6660
S. mucronatus	UMMZ	112608	México	México	19.3330	-98.7160
S. mucronatus	CNAR	11985	México	México	19.9394	-99.5647
S. mucronatus	CNAR	11987	México	México	20.1142	-99.6683
S. mucronatus	USNM	S/N / 34240	México	Veracruz	19.7000	-97.1500
S. mucronatus	ENCB	6506	México	Veracruz	19.5990	-97.0990
S. mucronatus	FMNH	70881-917	México	Veracruz	19.6000	-97.0500
S. mucronatus	MVZ	146916	México	Veracruz	19.6350	-97.0980
S. mucronatus	SNOMNH	34469	México	México	19.3217	-99.3739
S. mucronatus	EBUAP	11247	México	Distrito Federal	19.2080	-99.2580
S. mucronatus	ENCB	6885	México	Distrito Federal	19.2670	-99.2540
S. mucronatus	MZFC	185	México	Distrito Federal	19.2330	-99.2830
S. mucronatus	CNAR	874	México	Distrito Federal	19.2250	-99.2670
S. mucronatus	CNAR	9341	México	Distrito Federal	19.2300	-99.2800
S. mucronatus	ND	9999 / 534	México	Distrito Federal	19.2250	-99.2670
S. mucronatus	CNAR	AR2037	México	Hidalgo	20.6000	-98.8100
S. mucronatus	EBUAP	867	México	Distrito Federal	19.1040	-99.1170
S. mucronatus	ND	9999 / 489	México	Distrito Federal	19.1040	-99.1170
S. mucronatus	ENCB	4074	México	Hidalgo	20.2510	-98.7830
S. mucronatus	ENCB	7611	México	Hidalgo	20.2320	-98.7480
S. mucronatus	ND	47583	México	Hidalgo	20.2510	-98.7830

S. mucronatus	LACM	69057	México	Hidalgo	20.2200	-98.7300
S. mucronatus	UMMZ	114892	México	Hidalgo	20.1330	-98.6660
S. mucronatus	CNAR	AR2023	México	México	19.7700	-99.5900
S. mucronatus	KU	37758-759	México	Tlaxcala	19.5330	-98.5000
S. mucronatus	CNAR	AR2187	México	Hidalgo	20.1756	-99.5908
S. mucronatus	ND	S/N / 9873	México	México	19.3000	-99.3660
S. mucronatus	AMNH	65704-705	México	México	19.0500	-99.3160
S. mucronatus	CNAR	12122	México	México	19.0567	-99.3800
S. mucronatus	MCZ	31737	México	Hidalgo	20.1830	-98.6500
S. mucronatus	MZFC	5798	México	Puebla	19.3700	-97.5000
S. mucronatus	YPM	YPM HERR 007801	México	Puebla	19.4000	-97.6667
S. mucronatus	LSUMNS	36145	México	México	19.0133	-98.8008
S. mucronatus	MZFC	3303	México	Hidalgo	20.1830	-98.7500
S. mucronatus	ENCB	5084	México	Veracruz	19.5600	-97.2420
S. mucronatus	ENCB	5121	México	Veracruz	19.5600	-97.1700
S. mucronatus	MZFC	3474	México	Veracruz	19.5000	-97.3330
S. mucronatus	IUMNH	48957-958	México	Veracruz	19.4330	-97.3830
S. mucronatus	UMMZ	105017	México	Veracruz	19.6160	-97.3660
S. mucronatus	UMMZ	112962	México	Veracruz	19.5500	-97.2660
S. mucronatus	UMMZ	89307-309	México	Veracruz	19.5000	-97.3500
S. mucronatus	IUMNH	60892	México	Puebla	19.0830	-98.2000
S. mucronatus	ENCB	502	México	Hidalgo	20.6250	-98.6180
S. mucronatus	CNAR	5680	México	México	19.6950	-98.7817
S. mucronatus	MZFC	356	México	Tlaxcala	19.1900	-98.1200
S. mucronatus	ND	S/N / 30981	México	Tlaxcala	19.3660	-98.1500
S. mucronatus	CNAR	3772	México	México	20.0178	-99.5236
S. mucronatus	CNAR	11982	México	México	18.8969	-99.6114
S. mucronatus	IUMNH	8746-758	México	México	19.0660	-99.5500
S. mucronatus	CAS	54635	México	México	19.6830	-98.8660
S. mucronatus	MCZ	6340	México	México	19.6830	-98.8660
S. mucronatus	UMMZ	88638	México	Puebla	18.4830	-98.8000
S. mucronatus	ENCB	15832	México	Hidalgo	19.8090	-98.5020
S. mucronatus	ENCB	15833	México	Hidalgo	19.7840	-98.5520
S. mucronatus	MZFC	3332	México	Hidalgo	19.7660	-98.5660
S. mucronatus	MZFC	11578	México	México	19.5500	-98.7000
S. mucronatus	ENCB	16518	México	Puebla	19.5700	-97.4910
S. mucronatus	ND	S/N / 30983	México	Tlaxcala	19.4830	-98.1160
S. mucronatus	KU	37745	México	México	19.3856	-98.6667
S. mucronatus	MZFC	15288	México	Puebla	19.9000	-97.3200
S. mucronatus	ENCB	6649	México	Hidalgo	20.2090	-99.2730
S. mucronatus	MZFC	525	México	México	19.1550	-99.3078
S. mucronatus	UTEP	18588	México	México	19.1350	-99.2917
S. mucronatus	CNAR	4945	México	México	19.2500	-98.6667
S. mucronatus	ENCB	10279	México	Distrito Federal	19.2540	-99.1620
S. mucronatus	MZFC	158	México	Distrito Federal	19.2330	-99.2330
S. mucronatus	MZFC	189	México	Distrito Federal	19.1300	-99.1600
S. mucronatus	MZFC	2438	México	Distrito Federal	19.1600	-99.1900
S. mucronatus	MZFC	11676	México	Distrito Federal	19.1300	-99.1700
S. mucronatus	MZFC	14165	México	Distrito Federal	19.1900	-99.1900
S. mucronatus	MZFC	356	México	Tlaxcala	19.3160	-98.2000
S. mucronatus	MZFC	3477	México	Tlaxcala	19.6000	-98.0830
S. mucronatus	ENCB	8141	México	Hidalgo	20.0530	-99.3630
S. mucronatus	MZFC	70860	México	Hidalgo	20.0500	-99.3500
S. mucronatus	ENCB	923	México	Hidalgo	20.1300	-98.3360
S. mucronatus	FMNH	112244	México	Veracruz	19.6330	-97.1660
S. mucronatus	UANL	4819	México	Tlaxcala	19.4600	-98.2000
S. mucronatus	IUMNH	35320	México	Puebla	20.2830	-97.9500
S. mucronatus	UMMZ	106384	México	Veracruz	20.4160	-98.4660
S. mucronatus	ENCB	10943	México	Puebla	19.6960	-97.5740
S. oberon	CAS	7720	México	Nuevo León	24.7500	-99.8000
S. oberon	CM	50042	México	Nuevo León	25.4410	-100.1540
S. oberon	CM	2569	México	Nuevo León	25.4410	-100.1540
S. oberon	CM	4911	México	Nuevo León	25.4280	-100.1270
S. oberon	CM	R-28309	México	Nuevo León	25.4280	-100.1290
S. oberon	MZFC	10706	México	Coahuila	25.3000	-100.2800
S. oberon	MZFC	10674	México	Nuevo León	24.4100	-100.1200
S. oberon	MZFC	10716	México	Nuevo León	24.3900	-100.1000
S. oberon	MZFC	147640	México	Nuevo León	24.4100	-100.1200
S. oberon	MZFC	147659	México	Nuevo León	24.3200	-100.3600
S. oberon	MZFC	5347	México	Nuevo León	25.0330	-100.2500
S. oberon	MZFC	1689	México	Nuevo León	24.7833	-100.2067
S. oberon	MZFC	7718	México	Nuevo León	24.7833	-100.2067
S. oberon	MZFC	7719	México	Nuevo León	24.8500	-100.1500
S. oberon	MZFC	R-28289	México	Nuevo León	24.8270	-100.0760
S. oberon	MZFC	30179	México	Nuevo León	24.5330	-100.1000

S. oberon	FMNH	19136	México	Nuevo León	25.6460	-100.0920
S. oberon	CNAR	7716	México	Nuevo León	24.5900	-99.9933
S. oberon	CNAR	S/N / 15753 / A014	México	Nuevo León	24.9330	-100.1500
S. oberon	CNAR	48336-339	México	Nuevo León	25.0000	-100.2830
S. oberon	CNAR	5346 / 63536 / A014	México	Nuevo León	25.0330	-100.3160
S. oberon	CNAR	30728	México	Nuevo León	24.8160	-100.0830
S. oberon	CNAR	115866	México	Nuevo León	24.7833	-100.2067
S. oberon	LSUMNS	147672	México	Nuevo León	25.4000	-100.2400
S. oberon	MCZ	3938-1	México	Nuevo León	25.6460	-100.0920
S. oberon	ND	R-28307	México	Nuevo León	25.5990	-100.1580
S. oberon	ND	7422	México	Nuevo León	24.7500	-99.8000
S. oberon	ND	7717	México	Nuevo León	24.7167	-99.5333
S. oberon	ND	21314	México	Nuevo León	24.8500	-99.5500
S. oberon	ND	5346	México	Nuevo León	25.1000	-100.1900
S. oberon	ND	10725	México	Nuevo León	25.1100	-100.2800
S. oberon	ND	5347	México	Nuevo León	25.1000	-100.1500
S. oberon	ND	147677	México	Nuevo León	25.1100	-100.2800
S. oberon	ND	21305	México	Nuevo León	26.4160	-100.1330
S. oberon	ND	10709	México	Nuevo León	25.4000	-100.2300
S. oberon	ND	10719	México	Nuevo León	25.4000	-100.2400
S. oberon	ND	48158	México	Nuevo León	25.4280	-100.1290
S. oberon	USNM	33296	México	Nuevo León	25.0430	-100.3000
S. oberon	UCM	108827	México	Nuevo León	24.8760	-100.2200
S. oberon	IUMNH	R-149186	México	Nuevo León	24.5920	-99.9950
S. ornatus	CNAR	7673	México	Coahuila	25.5900	-101.8800
S. ornatus	CNAR	7674	México	Nuevo León	26.5200	-100.7200
S. ornatus	KU	NoDPhScor1	México	Coahuila	25.2160	-101.5330
S. ornatus	ND	NoDPhScor5	México	Coahuila	25.6500	-101.8660
S. ornatus	ND	NoDPhScor4	México	Coahuila	25.4160	-101.0000
S. ornatus	LACM	NoDPhScor2	México	Nuevo León	25.8600	-100.5300
S. ornatus	LACM	NoDPhScor3	México	Nuevo León	26.2400	-100.5700
S. ornatus	USNM	NoDPhScor10	México	Coahuila	25.6660	-101.1160
S. ornatus	TCWC	NoDPhScor6	México	Coahuila	27.1600	-101.2700
S. ornatus	TCWC	NoDPhScor7	México	Nuevo León	26.5520	-100.4730
S. ornatus	IUMNH	NoDPhScor8	México	Coahuila	25.0330	-101.0160
S. ornatus	IUMNH	NoDPhScor9	México	Coahuila	25.4660	-101.0000
S. ornatus	UTAMM	NoDPhScor11	México	Nuevo León	25.8090	-100.5930
S. ornatus	UTEP	NoDPhScor12	México	Coahuila	25.3200	-100.5600
S. omiltemanus	ND	112253	México	Guerrero	17.4480	-98.8700
S. omiltemanus	ND	61112	México	Guerrero	17.4840	-98.8740
S. omiltemanus	ENCB	6519	México	Guerrero	17.4480	-99.0230
S. omiltemanus	CNAR	10215	México	Guerrero	17.5083	-99.1183
S. omiltemanus	CNAR	AR5135	México	Guerrero	17.4000	-99.0230
S. omiltemanus	MCZ	R-136480	México	Guerrero	17.4480	-99.0230
S. omiltemanus	CAS	169606	México	Guerrero	17.5500	-99.7100
S. omiltemanus	MZFC	3969	México	Guerrero	17.5300	-99.5800
S. omiltemanus	MZFC	16454	México	Guerrero	17.3600	-99.5000
S. omiltemanus	MZFC	1283	México	Guerrero	17.5420	-99.6580
S. omiltemanus	MZFC	1284	México	Guerrero	17.5420	-99.6920
S. omiltemanus	MZFC	1287	México	Guerrero	17.5250	-99.6750
S. omiltemanus	MZFC	1290	México	Guerrero	17.5250	-99.6920
S. omiltemanus	MZFC	1296	México	Guerrero	17.5580	-99.6750
S. omiltemanus	MZFC	1306	México	Guerrero	17.5580	-99.6920
S. omiltemanus	CNAR	7558	México	Guerrero	17.5600	-99.6900
S. omiltemanus	MCZ	15096	México	Guerrero	17.5570	-99.6760
S. omiltemanus	MCZ	R-129544	México	Guerrero	17.5500	-99.6670
S. omiltemanus	ND	6435	México	Guerrero	17.4333	-99.5667
S. omiltemanus	ND	7837	México	Guerrero	17.5670	-99.6830
S. omiltemanus	ND	8759	México	Guerrero	17.5600	-99.5000
S. omiltemanus	ND	21349	México	Guerrero	17.5700	-99.6800
S. omiltemanus	ND	21351	México	Guerrero	17.5600	-99.6500
S. omiltemanus	ND	22048	México	Guerrero	17.5500	-99.6500
S. omiltemanus	ND	47590	México	Guerrero	17.5500	-99.7100
S. omiltemanus	ND	48956	México	Guerrero	17.5500	-99.6700
S. omiltemanus	ND	48958	México	Guerrero	17.5250	-99.6750
S. omiltemanus	ND	60467	México	Guerrero	17.5610	-99.6870
S. omiltemanus	ND	61093	México	Guerrero	17.4333	-99.5833
S. omiltemanus	ND	61102	México	Guerrero	17.5570	-99.6760
S. omiltemanus	ND	148893	México	Guerrero	17.5580	-99.6920
S. omiltemanus	ND	R-129424	México	Guerrero	17.5580	-99.6850
S. omiltemanus	ND	R-136483	México	Guerrero	17.5500	-99.6200
S. omiltemanus	ND	R-136492	México	Guerrero	17.5500	-99.6330
S. omiltemanus	ND	R-136493	México	Guerrero	17.5420	-99.6580
S. omiltemanus	ND	R-42728	México	Guerrero	17.5567	-99.6856
S. omiltemanus	LACM	109170	México	Guerrero	17.4330	-99.5660

S. omiltemanus	LACM	109193	México	Guerrero	17.4330	-99.5830
S. omiltemanus	USNM	47589	México	Guerrero	17.5600	-99.6900
S. omiltemanus	USNM	S/N / 5466	México	Guerrero	17.5660	-99.6830
S. omiltemanus	USNM	S/N / 5502	México	Guerrero	17.5500	-99.6160
S. omiltemanus	USNM	S/N / 5503	México	Guerrero	17.5660	-99.6160
S. omiltemanus	ND	61107	México	Guerrero	17.8900	-99.9800
S. omiltemanus	CAS	135269	México	Guerrero	17.5800	-99.8500
S. omiltemanus	CAS	143942	México	Guerrero	17.6180	-99.8400
S. omiltemanus	CNAR	6399	México	Guerrero	17.6600	-99.8300
S. omiltemanus	CNAR	6400	México	Guerrero	17.6700	-99.8500
S. omiltemanus	CNAR	6401	México	Guerrero	17.6500	-99.8300
S. omiltemanus	CNAR	6402	México	Guerrero	17.6600	-99.8400
S. omiltemanus	MCZ	R-136486	México	Guerrero	17.6183	-99.8400
S. omiltemanus	MVZ	143272	México	Guerrero	17.6210	-99.8380
S. omiltemanus	ND	25078	México	Guerrero	17.6183	-99.8500
S. omiltemanus	ND	36113	México	Guerrero	17.6183	-99.8400
S. omiltemanus	ND	47033	México	Guerrero	17.5700	-99.7000
S. omiltemanus	ND	112229	México	Guerrero	17.5800	-99.8500
S. omiltemanus	ND	R-13651	México	Guerrero	17.6040	-99.8340
S. omiltemanus	LACM	109223	México	Guerrero	17.6180	-99.8400
S. omiltemanus	LACM	127038	México	Guerrero	17.6180	-99.8500
S. omiltemanus	CNAR	6403	México	Guerrero	17.5600	-100.2900
S. omiltemanus	ENCB	7604	México	Guerrero	18.5740	-99.5860
S. omiltemanus	CNAR	6396	México	Guerrero	17.3000	-98.7500
S. omiltemanus	ND	R-42729	México	Guerrero	17.0100	-98.9200
S. sp.	MZFC	20633	MÉXICO	Jalisco	21.9177	-103.8774
S. sp.	MZFC	20634-37	MÉXICO	Jalisco	21.8980	-103.8604
S. sp.	UTA	55433-34	MÉXICO	Jalisco	21.8929	-103.8650
S. sp.	UTA	55437	MÉXICO	Nayarit	21.6736	-104.4074
S. palaciosi	MZFC	14179	México	Distrito Federal	19.3170	-99.2000
S. palaciosi	MZFC	7424	México	Puebla	19.1800	-98.4300
S. palaciosi	MZFC	922	México	México	18.8160	-99.7660
S. palaciosi	MZFC	990	México	Distrito Federal	19.3330	-99.2830
S. palaciosi	EBUM	2544	México	Morelos	19.0210	-99.2850
S. palaciosi	MZFC	543	México	Morelos	19.0340	-99.2010
S. palaciosi	CNAR	5953	México	Morelos	19.0500	-99.3000
S. palaciosi	CNAR	5955	México	Morelos	19.0300	-99.2100
S. palaciosi	UCM	8835	México	Morelos	19.0810	-99.2280
S. palaciosi	MZFC	790	México	México	19.3140	-98.7190
S. palaciosi	MZFC	920	México	México	19.3333	-98.7192
S. palaciosi	MZFC	865	México	México	19.1769	-99.3169
S. palaciosi	MZFC	850	México	Distrito Federal	19.2300	-99.2890
S. palaciosi	MZFC	11672	México	Distrito Federal	19.2390	-99.2540
S. palaciosi	MZFC	11673	México	Distrito Federal	19.2080	-99.2580
S. palaciosi	MZFC	867	México	México	19.7000	-99.1600
S. palaciosi	MZFC	1940	México	Distrito Federal	19.1000	-99.1200
S. palaciosi	EBUM	2527	México	México	19.0870	-99.3120
S. palaciosi	EBUM	2529	México	México	19.0810	-99.3240
S. palaciosi	EBUM	2533	México	México	19.0890	-99.3150
S. palaciosi	MZFC	543	México	México	19.0500	-99.3160
S. palaciosi	MZFC	810	México	México	19.0500	-99.3170
S. palaciosi	CNAR	5957	México	México	19.0567	-99.3133
S. palaciosi	MZFC	5937	México	México	19.8200	-98.8400
S. palaciosi	MZFC	1941	México	México	19.1500	-99.3000
S. palaciosi	MZFC	529	México	México	19.1350	-99.2920
S. palaciosi	MZFC	800	México	México	19.1550	-99.3078
S. palaciosi	MZFC	988	México	México	19.1360	-99.3000
S. palaciosi	CNAR	5212	México	México	19.1400	-99.2900
S. palaciosi	KU	197025	México	México	19.1350	-99.2917
S. palaciosi	MZFC	836	México	México	19.2000	-98.8160
S. palaciosi	CNAR	836	México	México	19.2036	-98.7808
S. palaciosi	CNAR	6819	México	Distrito Federal	19.2300	-99.2200
S. palaciosi	CNAR	6820	México	Distrito Federal	19.2100	-99.2100
S. palaciosi	MZFC	876	México	México	19.3900	-100.0917
S. palaciosi	MZFC	11804	México	México	19.8000	-99.1700
S. prezygus	CAS	163455	México	Chiapas	16.8000	-92.9050
S. prezygus	CAS	163914	México	Chiapas	16.3778	-91.8547
S. prezygus	CAS	163866	México	Chiapas	16.1310	-91.8681
S. prezygus	CAS	163866	México	Chiapas	16.1260	-91.7230
S. prezygus	ECO-SC-H	497	México	Chiapas	16.7860	-92.8260
S. prezygus	ECO-SC-H	489,495-6	México	Chiapas	16.7410	-92.9080
S. prezygus	ECO-SC-H	ECO-SCH-0489	México	Chiapas	16.4700	-92.4900
S. prezygus	MZFC	5937	México	Chiapas	16.1160	-91.7830
S. prezygus	CNAR	18085	México	Chiapas	16.2629	-92.0391
S. prezygus	CNAR	18099	México	Chiapas	16.7359	-92.9238

S. prezygus	CNAR	18084	México	Chiapas	16.2603	-92.0280
S. prezygus	CNAR	18081	México	Chiapas	16.1130	-92.0447
S. prezygus	CNAR	18082	México	Chiapas	16.1144	-92.0401
S. prezygus	CNAR	18093	México	Chiapas	16.8090	-90.8789
S. prezygus	CNAR	18027	México	Chiapas	16.5327	-92.4758
S. prezygus	CNAR	18104	México	Chiapas	16.5305	-92.4771
S. prezygus	CNAR	18105	Guatemala	Huehuetenango	15.3680	-91.2911
S. prezygus	CNAR	18109	Guatemala	Huehuetenango	15.3857	-91.3075
S. prezygus	CNAR	18112	Guatemala	Huehuetenango	15.3571	-91.2998
S. prezygus	CNAR	sincatálogo1	Guatemala	Huehuetenango	15.5900	-91.9400
S. prezygus	CNAR	18113	Guatemala	Quiché	15.3285	-91.0052
S. prezygus	CNAR	18122	Guatemala	Quiché	15.3548	-90.8438
S. prezygus	CZRHE	1226	México	Chiapas	16.7380	-92.8940
S. prezygus	CZRHE	1229	México	Chiapas	16.7410	-92.9080
S. prezygus	CZRHE	1327	México	Chiapas	16.3150	-91.9810
S. prezygus	CZRHE	419	México	Chiapas	16.5790	-92.5180
S. prezygus	CZRHE	1295	México	Chiapas	16.5860	-92.5100
S. prezygus	KU	59696	México	Chiapas	16.5210	-92.4240
S. prezygus	KU	59697	México	Chiapas	16.5150	-92.4010
S. prezygus	KU	94094	México	Chiapas	16.2470	-91.9630
S. prezygus	LACM	61961	México	Chiapas	16.6300	-92.5300
S. prezygus	LACM	61961	México	Chiapas	16.6300	-92.5300
S. prezygus	LACM	61962	México	Chiapas	16.6300	-92.5300
S. prezygus	LACM	129988	México	Chiapas	16.5940	-92.5220
S. prezygus	USNM	S/N / 2506	México	Chiapas	16.3330	-92.3160
S. prezygus	IUMNH	8776	México	Chiapas	16.5000	-92.3330
S. prezygus	IUMNH	8773	México	Chiapas	16.2510	-92.1340
S. prezygus	IUMNH	8779	México	Chiapas	16.3790	-92.2430
S. prezygus	IUMNH	8780	México	Chiapas	16.3790	-92.2460
S. prezygus	IUMNH	8772-775	México	Chiapas	16.3660	-92.1330
S. prezygus	IUMNH	8779-780	México	Chiapas	16.3660	-92.1660
S. prezygus	IUMNH	51009	México	Chiapas	16.7830	-92.9330
S. prezygus	IUMNH	87259	México	Chiapas	16.8080	-92.9110
S. prezygus	IUMNH	52088-09O	México	Chiapas	16.7330	-92.8830
S. prezygus	IUMNH	8781	México	Chiapas	16.5500	-92.4830
S. prezygus	UMMZ	94667	México	Chiapas	16.5110	-92.3710
S. prezygus	UMMZ	94091	México	Chiapas	16.2510	-92.1340
S. prezygus	UMMZ	94650	México	Chiapas	16.2250	-92.1110
S. prezygus	UMMZ	94652	México	Chiapas	16.2010	-92.1080
S. prezygus	UMMZ	94653	México	Chiapas	16.2500	-92.1170
S. prezygus	UMMZ	94655	México	Chiapas	16.2250	-92.1110
S. prezygus	UMMZ	94656	México	Chiapas	16.2510	-92.1340
S. prezygus	UMMZ	94660	México	Chiapas	16.3500	-92.2080
S. prezygus	UMMZ	94662	México	Chiapas	16.4530	-92.2720
S. prezygus	UMMZ	94665	México	Chiapas	16.4740	-92.2670
S. prezygus	UMMZ	119795	México	Chiapas	16.8400	-92.9070
S. prezygus	UMMZ	119794	México	Chiapas	16.7360	-92.6360
S. prezygus	UMMZ	126229	México	Chiapas	16.5920	-92.5170
S. prezygus	UMMZ	133306	México	Chiapas	16.6300	-92.5300
S. prezygus	UTEP	3937	México	Chiapas	16.0760	-92.0360
S. poinsettii	AMNH	737440	US	New Mexico	32.0139	-108.8667
S. poinsettii	ASU	2871	US	New Mexico	33.2272	-108.2717
S. poinsettii	ASU	40318	US	New Mexico	32.9125	-107.7903
S. poinsettii	CAS	203994	US	New Mexico	31.9489	-108.8751
S. poinsettii	CAS	100094	US	New Mexico	31.4092	-108.5376
S. poinsettii	CAS	3715	US	New Mexico	34.0830	-107.2047
S. poinsettii	MSB	11460	US	New Mexico	33.3597	-108.0653
S. poinsettii	MSB	4202	US	New Mexico	33.3500	-108.0789
S. poinsettii	MSB	13469	US	New Mexico	33.4833	-107.7694
S. poinsettii	MSB	20903	US	New Mexico	33.3800	-108.0900
S. poinsettii	MSB	22348	US	New Mexico	33.5600	-108.1300
S. poinsettii	MSB	41622	US	New Mexico	33.2500	-108.3200
S. poinsettii	MSB	4233	US	New Mexico	33.4014	-108.5786
S. poinsettii	MSB	42795	US	New Mexico	33.2600	-108.2900
S. poinsettii	MSB	74078	US	New Mexico	33.4000	-108.1000
S. poinsettii	MSB	13468	US	New Mexico	32.8417	-107.8917
S. poinsettii	MSB	23124	US	New Mexico	33.0333	-108.2167
S. poinsettii	MSB	23130	US	New Mexico	32.9142	-108.2236
S. poinsettii	MSB	40944	US	New Mexico	32.9167	-108.2250
S. poinsettii	MSB	17563	US	New Mexico	33.0300	-108.1700
S. poinsettii	MSB	23129	US	New Mexico	32.9200	-108.2200
S. poinsettii	MSB	23131	US	New Mexico	32.8996	-108.2221
S. poinsettii	MSB	4232	US	New Mexico	32.9800	-108.0700
S. poinsettii	MSB	4235	US	New Mexico	32.9100	-107.8200
S. poinsettii	MSB	52023	US	New Mexico	32.8400	-107.8600

S. poinsettii	MSB	52877	US	New Mexico	32.9100	-107.7661
S. poinsettii	MSB	4234	US	New Mexico	31.9400	-108.6100
S. poinsettii	MSB	63122	US	New Mexico	31.3700	-108.6800
S. poinsettii	MSB	4228	US	New Mexico	33.3700	-107.8900
S. poinsettii	MSB	60150	US	New Mexico	33.2056	-107.6044
S. poinsettii	MSB	66235	US	New Mexico	33.1600	-107.7400
S. poinsettii	MSB	71731	US	New Mexico	33.0800	-107.6900
S. poinsettii	MSB	25742	US	New Mexico	33.8750	-107.1333
S. poinsettii	MSB	11418	US	New Mexico	34.0200	-106.9400
S. poinsettii	MSB	13690	US	New Mexico	33.7700	-107.6100
S. poinsettii	MSB	20502	US	New Mexico	33.5000	-107.4000
S. poinsettii	MSB	32128	US	New Mexico	33.9100	-107.3900
S. poinsettii	MSB	4216	US	New Mexico	33.5958	-107.5833
S. poinsettii	MSB	4219	US	New Mexico	33.8100	-107.4200
S. poinsettii	MSB	4220	US	New Mexico	34.0000	-107.0000
S. poinsettii	MSB	4221	US	New Mexico	34.0333	-107.2333
S. poinsettii	MSB	49513	US	New Mexico	34.3500	-106.8800
S. poinsettii	MSB	50309	US	New Mexico	33.5600	-107.6200
S. poinsettii	MSB	73178	US	New Mexico	33.9500	-106.9700
S. poinsettii	MSB	73203	US	New Mexico	33.8600	-107.5800
S. poinsettii	MSB	73216	US	New Mexico	33.7900	-107.4900
S. poinsettii	MSB	73218	US	New Mexico	33.7200	-107.5200
S. poinsettii	MSB	73253	US	New Mexico	34.0000	-106.9900
S. poinsettii	MSB	73306	US	New Mexico	33.7600	-107.4300
S. poinsettii	MSB	73381	US	New Mexico	33.9200	-107.0800
S. poinsettii	MSB	73382	US	New Mexico	33.9300	-107.0900
S. poinsettii	MSB	73398	US	New Mexico	34.0800	-107.1500
S. poinsettii	MSB	73399	US	New Mexico	34.2900	-107.2700
S. poinsettii	MSB	73402	US	New Mexico	34.1900	-107.2200
S. poinsettii	MSB	74034	US	New Mexico	33.9800	-107.2600
S. poinsettii	MSB	74513	US	New Mexico	34.3000	-107.2800
S. poinsettii	MSB	74515	US	New Mexico	34.3000	-107.3000
S. poinsettii	MVZ	13841	US	New Mexico	32.7732	-108.0971
S. poinsettii	MVZ	42549	US	New Mexico	32.8656	-108.2177
S. poinsettii	MVZ	42550	US	New Mexico	32.7628	-108.2859
S. poinsettii	MVZ	42552	US	New Mexico	32.7975	-108.1473
S. poinsettii	MVZ	42553	US	New Mexico	32.7642	-108.0867
S. poinsettii	MVZ	42554	US	New Mexico	32.7747	-108.2922
S. poinsettii	MVZ	7051	US	New Mexico	32.5901	-108.4068
S. poinsettii	MVZ	79204	US	New Mexico	31.3958	-108.5583
S. poinsettii	MVZ	229646	US	New Mexico	31.4071	-108.5089
S. poinsettii	MVZ	79205	US	New Mexico	31.3958	-108.5616
S. poinsettii	MVZ	79206	US	New Mexico	31.3958	-108.5616
S. poinsettii	MVZ	69432	US	New Mexico	33.0149	-107.5486
S. poinsettii	GNHC	13707	US	New Mexico	33.2211	-108.2389
S. poinsettii	ND	13470	US	New Mexico	33.8000	-108.4000
S. poinsettii	GNHC	12181	US	New Mexico	32.8569	-108.2597
S. poinsettii	GNHC	12184	US	New Mexico	32.9422	-108.1489
S. poinsettii	GNHC	12738	US	New Mexico	32.9239	-108.2131
S. poinsettii	GNHC	12739	US	New Mexico	32.7961	-108.1497
S. poinsettii	GNHC	13009	US	New Mexico	32.5500	-108.3833
S. poinsettii	GNHC	13011	US	New Mexico	33.0122	-108.1064
S. poinsettii	GNHC	13013	US	New Mexico	32.9500	-108.0200
S. poinsettii	GNHC	13015	US	New Mexico	32.8978	-108.2450
S. poinsettii	GNHC	13019	US	New Mexico	32.7700	-108.2797
S. poinsettii	GNHC	13529	US	New Mexico	32.5278	-108.0986
S. poinsettii	GNHC	13580	US	New Mexico	32.4833	-108.4417
S. poinsettii	GNHC	13597	US	New Mexico	32.3208	-108.3222
S. poinsettii	ND	17565	US	New Mexico	33.0282	-108.7048
S. poinsettii	ND	62120	US	New Mexico	32.7975	-108.1473
S. poinsettii	ND	14161	US	New Mexico	32.7400	-108.0800
S. poinsettii	ND	13684	US	New Mexico	32.7900	-108.1100
S. poinsettii	ND	13814	US	New Mexico	32.7680	-108.0500
S. poinsettii	ND	40636	US	New Mexico	31.4092	-108.5376
S. poinsettii	GNHC	13008	US	New Mexico	33.3889	-107.6708
S. poinsettii	GNHC	13708	US	New Mexico	33.0500	-107.7625
S. poinsettii	ND	11010	US	New Mexico	33.5700	-107.5900
S. poinsettii	ND	13695	US	New Mexico	33.7100	-107.4500
S. poinsettii	ND	73227	US	New Mexico	33.9100	-106.9500
S. poinsettii	ND	73307	US	New Mexico	33.7300	-107.5600
S. poinsettii	LACM	4720	México	Chihuahua	31.3363	-108.3190
S. poinsettii	LACM	113619	US	New Mexico	32.9151	-108.2797
S. poinsettii	LACM	139719	US	New Mexico	32.9178	-108.2203
S. poinsettii	LACM	139721	US	New Mexico	32.9178	-108.2203
S. poinsettii	LACM	4721	US	New Mexico	32.9092	-107.8161

S. poinsettii	LACM	4722	US	New Mexico	32.9100	-107.7661
S. poinsettii	LACM	62120	US	New Mexico	32.8341	-108.2307
S. poinsettii	LACM	62121	US	New Mexico	32.8633	-108.2208
S. poinsettii	LACM	133323	US	New Mexico	32.0031	-108.7678
S. poinsettii	LACM	4714	US	New Mexico	31.3945	-108.5617
S. poinsettii	LACM	4718	US	New Mexico	31.3945	-108.5617
S. poinsettii	LACM	74223	US	New Mexico	33.1411	-107.5382
S. poinsettii	LACM	138114	US	New Mexico	34.0540	-107.1496
S. poinsettii	LACM	4723	US	New Mexico	33.7232	-107.4559
S. poinsettii	USNM	11005	US	New Mexico	34.0200	-107.1300
S. poinsettii	NMSU	3689	US	New Mexico	33.2167	-108.0500
S. poinsettii	NMSU	4111	US	New Mexico	33.2806	-108.3864
S. poinsettii	NMSU	4112	US	New Mexico	33.2528	-108.4389
S. poinsettii	SDNHM	24280	US	New Mexico	33.0120	-108.0901
S. poinsettii	SDNHM	25963	US	New Mexico	32.9083	-108.0061
S. poinsettii	SDNHM	26114	US	New Mexico	32.9722	-107.9792
S. poinsettii	TCWC	52877	US	New Mexico	32.9100	-107.7700
S. poinsettii	UCM	11073	US	New Mexico	33.9306	-108.3917
S. poinsettii	UCM	6183-89	US	New Mexico	33.8875	-108.3111
S. poinsettii	UCM	6190-6207	US	New Mexico	33.9028	-108.2083
S. poinsettii	UCM	6208	US	New Mexico	33.7583	-108.2411
S. poinsettii	UCM	6209	US	New Mexico	33.7564	-108.0806
S. poinsettii	UCM	13703	US	New Mexico	32.8133	-108.0906
S. poinsettii	UCM	6211	US	New Mexico	33.4639	-107.8139
S. poinsettii	UTA	17401	US	New Mexico	33.0282	-108.7048
S. poinsettii	UTA	17418	US	New Mexico	33.3467	-107.6467
S. poinsettii	UTA	17412	US	New Mexico	34.0483	-107.1847
S. poinsettii	UTEP	11506	US	New Mexico	32.5694	-108.4667
S. poinsettii	UTEP	11155	US	New Mexico	31.9431	-108.8833
S. poinsettii	UTEP	12428	US	New Mexico	31.3708	-108.6750
S. poinsettii	UTEP	16398	US	New Mexico	31.9806	-108.1625
S. poinsettii	UTEP	16078	US	New Mexico	32.7889	-107.6681
S. poinsettii	UTEP	13693	US	New Mexico	33.7200	-107.6700
S. poinsettii	UTEP	13750	US	New Mexico	33.9139	-107.7042
S. poinsettii	UTEP	4221	US	New Mexico	34.0300	-107.1700
S. poinsettii	Herp	14562	US	New Mexico	33.9900	-108.1500
S. poinsettii	Herp	14155	US	New Mexico	32.7600	-108.0320
S. poinsettii	Herp	12181	US	New Mexico	32.8600	-108.2600
S. poinsettii	Herp	13013	US	New Mexico	32.9556	-108.0250
S. poinsettii	Herp	13684	US	New Mexico	32.7747	-108.2922
S. poinsettii	Herp	13814	US	New Mexico	32.7700	-108.3000
S. poinsetti	UTEP	3632	México	Nuevo León	23.6917	-100.3917
S. poinsetti	UTEP	4327	México	Nuevo León	24.2083	-100.4833
S. poinsetti	RWA	6161	México	Nuevo León	24.5658	-100.2736
S. poinsetti	UTEP	6085	México	San Luis Potosí	23.8278	-100.5181
S. poinsetti	RWA	5731-5733	México	San Luis Potosí	23.8278	-100.5181
S. poinsetti	UTEP	3729	México	San Luis Potosí	24.0750	-101.1333
S. poinsetti	UTEP	3729-30	México	San Luis Potosí	23.9699	-101.0577
S. poinsetti	UTEP	3614	México	Zacatecas	24.2958	-101.6958
S. poinsetti	UTEP	6224	México	Zacatecas	24.0167	-101.3833
S. poinsetti	UTEP	8911-8919	México	Zacatecas	24.0164	-101.7976
S. poinsetti	UTEP	6751-52	México	Zacatecas	24.0106	-101.6140
S. poinsetti	UTEP	3618-23	México	Zacatecas	24.0916	-101.3191
S. serrifer	KU	171426	México	Yucatán	21.2000	-89.2000
S. serrifer	CNAR	18137	México	Campeche	18.2800	-91.5564
S. serrifer	CNAR	sincatálogo1	México	Campeche	18.3214	-91.5450
S. serrifer	CNAR	sincatálogo2	México	Campeche	18.3389	-91.5819
S. serrifer	CNAR	AR9207	México	Yucatán	21.2900	-89.5900
S. serrifer	CNAR	18067	México	Yucatán	20.6833	-87.8333
S. serrifer	MZFC	5475	México	Yucatán	21.1330	-90.0160
S. serrifer	FMNH	49235-238	México	Yucatán	20.6660	-88.5660
S. serrifer	CNAR	18132	México	Yucatán	20.6324	-89.4588
S. serrifer	CM	93172	México	Yucatán	20.9800	-89.6210
S. serrifer	ENCB	1277	México	Yucatán	21.0480	-89.6210
S. serrifer	ENCB	1644	México	Yucatán	21.0970	-89.5990
S. serrifer	FMNH	32282	México	Yucatán	20.9680	-89.6220
S. serrifer	FMNH	32277-299	México	Yucatán	20.9660	-89.6160
S. serrifer	CNAR	18021	México	Yucatán	21.0839	-89.6358
S. serrifer	SDNHM	16306	México	Yucatán	20.9660	-89.6160
S. serrifer	UCM	12937	México	Yucatán	21.0480	-89.6210
S. serrifer	UCM	12946	México	Yucatán	21.0580	-89.6210
S. serrifer	UCM	16773-794	México	Yucatán	21.1000	-89.6330
S. serrifer	UMMZ	72886	México	Yucatán	20.9840	-89.6220
S. serrifer	UMMZ	72890	México	Yucatán	21.1080	-89.6020
S. serrifer	UMMZ	72890	México	Yucatán	21.0970	-89.5990

S. serrifer	UCM	28698	México	Yucatán	21.0960	-89.2720
S. serrifer	UCM	29110	México	Yucatán	21.1000	-89.2830
S. serrifer	CNAR	sincatálogo3	México	Chiapas	16.9022	-90.9668
S. serrifer	UMMZ	113546	México	Yucatán	20.5660	-89.9160
S. serrifer	UCM	28682-689	México	Yucatán	21.3160	-88.1660
S. serrifer	CNAR	18079	México	Yucatán	21.1931	-89.6506
S. serrifer	KU	171427	México	Yucatán	20.9170	-88.8580
S. serrifer	KU	299993	México	Yucatán	20.3700	-89.2700
S. serrifer	CM	38932	México	Yucatán	20.2400	-89.3100
S. serrifer	UCM	28690-691	México	Yucatán	21.2330	-89.2660
S. serrifer	UCM	28690	México	Yucatán	21.2410	-89.2720
S. serrifer	UCM	28692	México	Yucatán	21.2670	-89.2720
S. serrifer	UCM	28694	México	Yucatán	21.2670	-89.2720
S. serrifer	UCM	28692-694	México	Yucatán	21.2660	-89.2660
S. serrifer	KU	157446	México	Yucatán	21.2010	-88.8750
S. serrifer	UCM	28691	México	Yucatán	21.2010	-88.8750
S. serrifer	CNAR	18055	México	Tabasco	17.4494	-91.4917
S. serrifer	KU	157447	México	Yucatán	20.9740	-89.9150
S. serrifer	ENCB	14683	México	Yucatán	21.5830	-88.0460
S. serrifer	CNAR	18123	México	Yucatán	21.3203	-88.1319
S. serrifer	CNAR	18124	México	Yucatán	21.3199	-88.1018
S. serrifer	CNAR	18125	México	Yucatán	21.3217	-88.1442
S. serrifer	CNAR	18128	México	Yucatán	21.3201	-88.1507
S. serrifer	CNAR	18129	México	Yucatán	21.1404	-88.1488
S. serrifer	UCM	28683	México	Yucatán	21.3350	-88.1510
S. sugillatus	UAEM	9999	México	México	19.0500	-99.3170
S. sugillatus	MZFC	891	México	México	19.0500	-99.3160
S. sugillatus	MZFC	6861	México	México	18.9800	-99.4170
S. sugillatus	MZFC	161	México	Morelos	18.5800	-99.3700
S. sugillatus	TCWC	6878	México	Morelos	19.0810	-99.2280
S. sugillatus	MZFC	162	México	Morelos	19.0660	-99.3000
S. sugillatus	IUMNH	16840-843	México	Morelos	19.0330	-99.2830
S. tamaulipensis	UANL	4762	México	Tamaulipas	23.4280	-98.6390
S. tamaulipensis	ND	Sin catálogo8	México	Tamaulipas	23.3400	-98.6000
S. tamaulipensis	TCWC	TCWC57344	México	Tamaulipas	23.1992	-98.4442
S. tamaulipensis	TCWC	TCWC57386	México	Tamaulipas	23.1627	-98.4313
S. tamaulipensis	UMMZ	101509	México	Tamaulipas	23.1760	-98.4250
S. torquatus	ENCB	14867	México	Guanajuato	20.4300	-101.5886
S. torquatus	MZFC	3294	México	México	20.0000	-99.9700
S. torquatus	CNAR	11945	México	México	20.0017	-99.8786
S. torquatus	CNAR	AR656	México	Hidalgo	20.2700	-98.9200
S. torquatus	KU	61714	México	México	20.0722	-99.8500
S. torquatus	KU	67611	México	México	20.1194	-99.8639
S. torquatus	KU	67614	México	México	20.0719	-99.8500
S. torquatus	CIB-UAEH	CIB-UAEH-459	México	Hidalgo	20.0700	-99.1000
S. torquatus	MZFC	5517	México	Guanajuato	20.8330	-100.7670
S. torquatus	KU	38159-160	México	Guanajuato	21.0330	-100.7160
S. torquatus	ENCB	1060	México	Distrito Federal	19.3330	-99.1960
S. torquatus	MZFC	545	México	Distrito Federal	19.2800	-99.2800
S. torquatus	MZFC	789	México	Distrito Federal	19.3160	-99.2000
S. torquatus	CNAR	251	México	Distrito Federal	19.3200	-99.2000
S. torquatus	MCZ	16090	México	Distrito Federal	19.3330	-99.1830
S. torquatus	UMMZ	99033	México	Distrito Federal	19.3500	-99.2000
S. torquatus	UMMZ	99926	México	Distrito Federal	19.3500	-99.1830
S. torquatus	MZFC	9334	México	Querétaro	20.1820	-100.1630
S. torquatus	MZFC	9341	México	Querétaro	20.1540	-100.1650
S. torquatus	MZFC	9342	México	Querétaro	20.1380	-100.1570
S. torquatus	TCWC	40921	México	Querétaro	20.1620	-100.1130
S. torquatus	TCWC	40922-40927	México	Querétaro	20.1420	-100.1300
S. torquatus	TCWC	40928-40931	México	Querétaro	20.0650	-100.0630
S. torquatus	TCWC	52965-52971	México	Querétaro	20.1710	-100.1530
S. torquatus	UTEP	9113	México	Querétaro	20.2410	-100.1540
S. torquatus	UTEP	9146	México	Querétaro	20.1370	-100.1140
S. torquatus	LACM	17359	México	México	19.1250	-98.7736
S. torquatus	TCWC	29551-29553	México	Querétaro	21.2860	-99.4440
S. torquatus	AMNH	15490	México	México	19.5330	-99.2330
S. torquatus	CNAR	11923	México	México	19.7989	-99.8717
S. torquatus	KU	67615	México	México	19.8594	-99.8561
S. torquatus	FMNH	112243	México	Hidalgo	20.3660	-98.7330
S. torquatus	AMNH	18473	México	Distrito Federal	19.4830	-99.2000
S. torquatus	AMNH	15582	México	Distrito Federal	19.4000	-99.1500
S. torquatus	CAS	73459-469	México	Distrito Federal	19.3830	-99.1660
S. torquatus	MZFC	8423	México	Querétaro	20.8780	-99.6120
S. torquatus	MZFC	9858	México	Querétaro	20.8600	-99.5700
S. torquatus	MZFC	14291	México	Querétaro	20.8940	-99.6520

S. torquatus	MVZ	36773	México	Querétaro	20.8780	-99.6130
S. torquatus	LACM	109145	México	Querétaro	20.9000	-99.6300
S. torquatus	TCWC	40946	México	Querétaro	20.8620	-99.6240
S. torquatus	TCWC	57168	México	Querétaro	20.6980	-99.7520
S. torquatus	MZFC	5742	México	México	19.1000	-99.7000
S. torquatus	FMNH	997	México	Guanajuato	20.5160	-100.8160
S. torquatus	CNAR	3807	México	México	19.8200	-99.6167
S. torquatus	MZFC	3333	México	Hidalgo	20.2330	-99.5500
S. torquatus	CIB-UAEH	CIB-UAEH-167	México	Hidalgo	20.2720	-99.2510
S. torquatus	MZFC	9616	México	Querétaro	20.9090	-100.2020
S. torquatus	MVZ	79879	México	Querétaro	20.5870	-100.2090
S. torquatus	CAS	73463	México	Distrito Federal	19.3500	-99.1300
S. torquatus	ENCB	899	México	Distrito Federal	19.3240	-99.1800
S. torquatus	ENCB	2077	México	Distrito Federal	19.3270	-99.1200
S. torquatus	FMNH	32795-796	México	Distrito Federal	19.3000	-99.1500
S. torquatus	MCZ	9597	México	Distrito Federal	19.3500	-99.1660
S. torquatus	FMNH	32783-786	México	México	19.7830	-99.2330
S. torquatus	MZFC	4355	México	Distrito Federal	19.3000	-99.3500
S. torquatus	MZFC	120	México	Distrito Federal	19.4200	-99.1300
S. torquatus	MVZ	32247	México	Distrito Federal	19.4310	-99.1300
S. torquatus	MZFC	5733	México	Hidalgo	19.9500	-98.3000
S. torquatus	AMNH	15604-605	México	México	19.6330	-99.1830
S. torquatus	CIB-UAEH	CIB-UAEH-593	México	Guanajuato	20.5940	-101.6450
S. torquatus	EBUM	414	México	Morelos	18.9800	-99.3100
S. torquatus	ENCB	5364	México	Morelos	18.9840	-99.2500
S. torquatus	MZFC	5742	México	Morelos	18.9670	-99.2840
S. torquatus	MZFC	8486	México	Morelos	18.9850	-99.3175
S. torquatus	FMNH	32754	México	Morelos	18.9330	-99.2330
S. torquatus	MZFC	833	México	México	19.3800	-100.2240
S. torquatus	IUMNH	20922	México	México	19.2917	-100.0958
S. torquatus	CAS	38803	México	Hidalgo	20.2500	-98.8670
S. torquatus	TCWC	11483	México	Querétaro	20.6310	-100.2630
S. torquatus	CIB-UAEH	CIB-UAEH-459	México	Hidalgo	20.1010	-98.6060
S. torquatus	ENCB	14814	México	Hidalgo	20.1090	-98.6020
S. torquatus	MZFC	3297	México	Michoacán	20.1190	-100.2050
S. torquatus	MZFC	9335	México	Michoacán	20.1620	-100.1850
S. torquatus	MZFC	9336	México	Michoacán	20.1190	-100.2050
S. torquatus	LACM	109144	México	Michoacán	20.1630	-100.1730
S. torquatus	TCWC	52960	México	Michoacán	20.2660	-100.2880
S. torquatus	UTEP	4822	México	Michoacán	20.1900	-100.3400
S. torquatus	MZFC	7892	México	Guanajuato	21.0830	-101.1880
S. torquatus	USNM	S/N / 6560	México	Guanajuato	21.0660	-101.2000
S. torquatus	UMMZ	99034	México	Distrito Federal	19.5330	-99.1660
S. torquatus	UTEP	4582	México	Hidalgo	20.3700	-99.6500
S. torquatus	EBUM	2525	México	Morelos	19.0360	-99.3020
S. torquatus	MZFC	118	México	Morelos	19.0660	-99.3000
S. torquatus	MZFC	792	México	Morelos	19.0170	-99.2500
S. torquatus	MZFC	3473	México	Morelos	19.0160	-99.2500
S. torquatus	MZFC	9999	México	Morelos	19.0840	-99.2380
S. torquatus	FMNH	65490	México	Morelos	19.0500	-99.3000
S. torquatus	FMNH	106934	México	Morelos	19.0500	-99.2330
S. torquatus	MVZ	78284	México	Morelos	19.0810	-99.2280
S. torquatus	MVZ	144166	México	Morelos	19.0400	-99.3050
S. torquatus	ND	9999 / 638	México	Morelos	19.0210	-99.2360
S. torquatus	LACM	58196	México	Morelos	19.0300	-99.2900
S. torquatus	ROM	13490	México	Morelos	19.0510	-99.3030
S. torquatus	EBUM	409	México	Morelos	19.0350	-99.2000
S. torquatus	IUMNH	20917	México	Morelos	19.0830	-99.2000
S. torquatus	ENCB	7313	México	Hidalgo	20.5490	-99.2190
S. torquatus	MZFC	3299	México	Hidalgo	20.4800	-99.1500
S. torquatus	UCM	28728	México	México	19.3333	-98.6706
S. torquatus	IUMNH	20921	México	México	19.3317	-98.7236
S. torquatus	UMMZ	117469	México	México	19.3330	-98.8500
S. torquatus	CNAR	AR704	México	México	19.5050	-99.7633
S. torquatus	MVZ	71247	México	México	19.5444	-99.7681
S. torquatus	MZFC	3405	México	Distrito Federal	19.3160	-99.0330
S. torquatus	MZFC	3406	México	Distrito Federal	19.3200	-99.0200
S. torquatus	MZFC	3504	México	Distrito Federal	19.3170	-99.0330
S. torquatus	MCZ	33902	México	Distrito Federal	19.3830	-99.0330
S. torquatus	ENCB	10287	México	Hidalgo	21.0100	-99.2000
S. torquatus	MZFC	11197	México	Hidalgo	21.0000	-99.1200
S. torquatus	FMNH	32736	México	Hidalgo	20.9830	-99.1830
S. torquatus	FMNH	32769-777	México	Hidalgo	20.9160	-99.2000
S. torquatus	IUMNH	8867	México	Hidalgo	21.0000	-99.2000
S. torquatus	MZFC	871	México	México	19.1769	-99.3169

S. torquatus	MZFC	8427	México	Querétaro	21.2300	-99.4400
S. torquatus	MZFC	8872	México	Querétaro	21.4210	-99.2420
S. torquatus	MZFC	8971	México	Querétaro	21.3980	-99.2520
S. torquatus	MZFC	12020	México	Querétaro	21.4000	-99.2400
S. torquatus	CNAR	4050	México	Querétaro	21.2480	-99.5000
S. torquatus	TCWC	29554	México	Querétaro	21.2640	-99.4440
S. torquatus	TCWC	33071	México	Querétaro	21.1310	-99.4850
S. torquatus	TCWC	38429	México	Querétaro	21.1670	-99.5050
S. torquatus	TCWC	32719-32722	México	Querétaro	21.1380	-99.4380
S. torquatus	CNAR	3805	México	México	19.8700	-99.5278
S. torquatus	CNAR	11935	México	México	19.9394	-99.5647
S. torquatus	CNAR	11936	México	México	19.8850	-99.5594
S. torquatus	MZFC	5034	México	México	19.6730	-99.7470
S. torquatus	CNAR	11934	México	México	19.7133	-99.7856
S. torquatus	MZFC	11799	México	Morelos	18.5800	-99.2000
S. torquatus	MZFC	6921	México	Querétaro	21.2830	-99.1330
S. torquatus	MZFC	6922	México	Querétaro	21.2670	-99.1370
S. torquatus	MZFC	9130	México	Querétaro	21.3722	-99.2261
S. torquatus	MZFC	9133	México	Querétaro	21.2970	-99.1790
S. torquatus	MZFC	9287	México	Querétaro	21.2860	-99.1790
S. torquatus	MZFC	9291	México	Querétaro	21.2980	-99.1760
S. torquatus	MZFC	9292	México	Querétaro	21.3830	-99.1950
S. torquatus	MZFC	9911	México	Querétaro	21.2667	-99.1500
S. torquatus	MZFC	9926	México	Querétaro	21.2720	-99.1870
S. torquatus	MVZ	129266	México	Querétaro	21.3230	-99.2240
S. torquatus	MVZ	129267	México	Querétaro	21.3250	-99.2289
S. torquatus	MVZ	129269	México	Querétaro	21.3250	-99.2280
S. torquatus	MVZ	186504	México	Querétaro	21.1800	-99.3400
S. torquatus	LACM	106774	México	Querétaro	21.2850	-99.1750
S. torquatus	LACM	109761	México	Querétaro	21.2870	-99.1760
S. torquatus	TCWC	29596	México	Querétaro	21.2870	-99.1170
S. torquatus	TCWC	32725	México	Querétaro	21.2720	-99.2010
S. torquatus	TCWC	32728	México	Querétaro	21.2950	-99.2560
S. torquatus	TCWC	36631	México	Querétaro	21.2920	-99.1590
S. torquatus	TCWC	37666	México	Querétaro	21.2920	-99.1280
S. torquatus	TCWC	27776-27788	México	Querétaro	21.2860	-99.1760
S. torquatus	TCWC	29597-29612	México	Querétaro	21.2890	-99.2620
S. torquatus	TCWC	32711-32714	México	Querétaro	21.2920	-99.1370
S. torquatus	TCWC	32723-32724	México	Querétaro	21.2800	-99.1220
S. torquatus	TCWC	32726-32727	México	Querétaro	21.2850	-99.2510
S. torquatus	TCWC	35841-35844	México	Querétaro	21.3430	-99.1970
S. torquatus	TCWC	57253-57255	México	Querétaro	21.2910	-99.1090
S. torquatus	UTA	11821	México	Querétaro	21.2850	-99.2650
S. torquatus	MVZ	76313	México	Distrito Federal	19.3330	-99.2000
S. torquatus	UMMZ	99928	México	Distrito Federal	19.3160	-99.2160
S. torquatus	USNM	S/N / 52672	México	Distrito Federal	19.3000	-99.2160
S. torquatus	CNAR	AR697	México	Hidalgo	20.5300	-98.7900
S. torquatus	MZFC	11059	México	Hidalgo	20.2240	-98.7590
S. torquatus	USNM	S/N / 7132	México	Hidalgo	20.2000	-98.7160
S. torquatus	CNAR	AR679	México	Hidalgo	20.7700	-99.1500
S. torquatus	MZFC	3462	México	Hidalgo	20.2330	-99.5780
S. torquatus	ENCB	508	México	Michoacán	19.5450	-100.3210
S. torquatus	ND	NA / 1207	México	Michoacán	19.5310	-100.3010
S. torquatus	ND	NA / 1911	México	Michoacán	19.5340	-100.2620
S. torquatus	ND	NA / 2287	México	Michoacán	19.5270	-100.3590
S. torquatus	ND	NA / 552	México	Michoacán	19.5430	-100.2680
S. torquatus	CNAR	2746	México	México	19.2703	-99.4583
S. torquatus	MZFC	123	México	México	19.1010	-99.3030
S. torquatus	MZFC	890	México	México	19.0500	-99.3170
S. torquatus	MZFC	7584	México	México	18.9800	-99.4170
S. torquatus	CNAR	2879	México	México	19.0469	-99.3150
S. torquatus	CNAR	2952	México	México	19.0750	-99.3122
S. torquatus	TCWC	858	México	México	19.0522	-99.3133
S. torquatus	UCM	50089	México	México	19.0517	-99.3122
S. torquatus	UCM	50089-090	México	México	19.0500	-99.3160
S. torquatus	ENCB	12217	México	Hidalgo	20.2300	-98.6500
S. torquatus	TCWC	54441	México	México	19.6803	-98.7806
S. torquatus	UMMZ	99813	México	Hidalgo	20.9330	-99.3160
S. torquatus	ND	S/N / 23713	México	Querétaro	21.1000	-99.7330
S. torquatus	TCWC	32699	México	Querétaro	21.1010	-99.9560
S. torquatus	TCWC	32697-32698	México	Querétaro	21.0330	-99.8970
S. torquatus	TCWC	32701-32705	México	Querétaro	21.1010	-99.8660
S. torquatus	TCWC	32715-32716	México	Querétaro	21.1010	-99.7400
S. torquatus	TCWC	40934-40935	México	Querétaro	20.9620	-99.7230
S. torquatus	MZFC	8426	México	Querétaro	21.1200	-99.5600

S. torquatus	MZFC	9134	México	Querétaro	21.1560	-99.6140
S. torquatus	CNAR	4051	México	Querétaro	21.1320	-99.6250
S. torquatus	TCWC	45450	México	Querétaro	21.1360	-99.6250
S. torquatus	TCWC	32717-32718	México	Querétaro	21.1330	-99.6610
S. torquatus	AMNH	118362	México	Querétaro	20.7330	-100.4540
S. torquatus	MZFC	9597	México	Querétaro	20.8470	-100.4950
S. torquatus	MZFC	9598	México	Querétaro	20.8150	-100.5440
S. torquatus	MZFC	9609	México	Querétaro	20.8150	-100.5440
S. torquatus	MZFC	9610	México	Querétaro	20.8180	-100.5290
S. torquatus	CNAR	11924	México	México	19.7117	-99.9639
S. torquatus	CNAR	11927	México	México	19.6133	-99.8867
S. torquatus	CNAR	11928	México	México	19.6617	-99.9617
S. torquatus	MZFC	9612	México	Querétaro	20.9280	-99.5550
S. torquatus	MZFC	9613	México	Querétaro	20.9420	-99.5590
S. torquatus	CNAR	3707	México	Querétaro	20.9230	-99.6010
S. torquatus	TCWC	40936-40945	México	Querétaro	20.9120	-99.5440
S. torquatus	TCWC	40952-40959	México	Querétaro	20.9410	-99.5720
S. torquatus	TCWC	54434	México	Querétaro	20.3870	-99.9950
S. torquatus	TCWC	52985-52993	México	Querétaro	20.3560	-100.1360
S. torquatus	MZFC	3323	México	México	19.7017	-98.8317
S. torquatus	CNAR	4033	México	México	19.7417	-98.8333
S. torquatus	MZFC	7582	México	Puebla	19.3300	-98.4800
S. torquatus	CNAR	3773	México	México	20.0178	-99.5236
S. torquatus	MZFC	14497	México	México	19.0700	-100.0700
S. torquatus	CNAR	2813	México	México	18.9625	-99.5944
S. torquatus	CNAR	11931	México	México	19.0022	-99.5778
S. torquatus	AMNH	15607-613	México	México	19.7500	-99.1500
S. torquatus	CAS	54635-636	México	México	19.6830	-98.8660
S. torquatus	LACM	97419	México	México	19.6850	-98.8400
S. torquatus	IUMNH	20923	México	México	19.6833	-98.8600
S. torquatus	MZFC	3290	México	Hidalgo	19.8670	-98.4170
S. torquatus	MZFC	3466	México	Hidalgo	19.8660	-98.4160
S. torquatus	ENCB	414	México	Morelos	18.9830	-99.1000
S. torquatus	MZFC	117	México	Morelos	19.0330	-99.1830
S. torquatus	MZFC	541	México	Morelos	19.0330	-99.1500
S. torquatus	MZFC	7583	México	Morelos	18.9860	-99.2010
S. torquatus	CNAR	1995	México	Morelos	19.0330	-99.0840
S. torquatus	ND	not recorded / 863	México	Morelos	18.9860	-99.2030
S. torquatus	IUMNH	55050-051	México	Morelos	19.0160	-99.1660
S. torquatus	MVZ	8850	México	México	19.9094	-99.1447
S. torquatus	ENCB	15349	México	Morelos	18.9440	-98.6810
S. torquatus	MZFC	11823	México	Morelos	18.8920	-98.7280
S. torquatus	MZFC	14282	México	México	19.1300	-99.5000
S. torquatus	MZFC	522	México	México	19.1360	-99.3000
S. torquatus	MZFC	3383	México	México	19.1350	-99.2917
S. torquatus	ENCB	411	México	Morelos	19.0390	-98.9430
S. torquatus	ENCB	410	México	Distrito Federal	19.2420	-99.2110
S. torquatus	ENCB	422	México	Distrito Federal	19.2680	-99.2070
S. torquatus	ENCB	1035	México	Distrito Federal	19.2000	-99.2370
S. torquatus	ENCB	1666	México	Distrito Federal	19.1360	-99.1430
S. torquatus	ENCB	1671	México	Distrito Federal	19.1360	-99.1430
S. torquatus	ENCB	1686	México	Distrito Federal	19.1330	-99.1710
S. torquatus	ENCB	1687	México	Distrito Federal	19.1360	-99.1480
S. torquatus	ENCB	1837	México	Distrito Federal	19.2750	-99.1490
S. torquatus	ENCB	13057	México	Distrito Federal	19.2750	-99.1390
S. torquatus	ENCB	15338	México	Distrito Federal	19.0830	-99.1430
S. torquatus	MZFC	618	México	Distrito Federal	19.2330	-99.2330
S. torquatus	MZFC	870	México	Distrito Federal	19.2160	-99.2160
S. torquatus	MZFC	993	México	Distrito Federal	19.1160	-99.2000
S. torquatus	MZFC	4356	México	Distrito Federal	19.2700	-99.1500
S. torquatus	MZFC	6344	México	Distrito Federal	19.2170	-99.2170
S. torquatus	ND	S/N / 13050	México	Distrito Federal	19.1330	-99.1830
S. torquatus	ND	S/N / 52419	México	Distrito Federal	19.2000	-99.1500
S. torquatus	ND	S/N / 52682	México	Distrito Federal	19.1000	-99.2160
S. torquatus	LACM	58197	México	Distrito Federal	19.1000	-99.2100
S. torquatus	CNAR	AR682	México	Hidalgo	19.8100	-98.5800
S. torquatus	MZFC	892	México	México	19.2889	-99.7044
S. torquatus	FMNH	70793	México	México	19.2883	-99.7028
S. torquatus	FMNH	70792-794	México	México	19.2830	-99.6830
S. torquatus	CNAR	11921	México	México	19.2972	-99.6542
S. torquatus	MVZ	76305	México	México	19.3330	-99.6860
S. torquatus	MVZ	76314	México	México	19.3333	-99.6903
S. torquatus	ENCB	16399	México	Hidalgo	19.9840	-99.3280
S. torquatus	MZFC	106	México	México	19.7000	-99.1200
S. torquatus	MZFC	4321	México	México	19.1590	-100.1260

S. torquatus	CNAR	992	México	México	19.1933	-100.1294
S. torquatus	ND	S/N / 11378	México	Guanajuato	20.3330	-101.1830
S. torquatus	SNOMNH	32690	México	Guanajuato	20.3900	-101.1817
S. torquatus	CNAR	7048	México	México	19.3900	-100.0917
S. torquatus	UMMZ	99927 / 52655	México	México	19.3500	-100.1830
S. torquatus	ENCB	3143	México	Hidalgo	19.9520	-98.8410
S. torquatus	ENCB	6843	México	Hidalgo	19.8960	-98.8210
S. torquatus	CNAR	3806	México	México	19.6700	-99.5161
S. torquatus	MZFC	6611	México	México	19.4128	-99.9239
S. torquatus	ND	NA / 1722	México	México	19.5060	-100.2060
S. torquatus	MZFC	6900	México	Guanajuato	21.3000	-100.0500
S. torquatus	AMNH	15590	México	Distrito Federal	19.2330	-99.1160
S. torquatus	ENCB	1614	México	Distrito Federal	19.2480	-99.0210
S. torquatus	LACM	36700	México	Distrito Federal	19.2200	-99.0500
S. torquatus	IUMNH	20930-932	México	Distrito Federal	19.2000	-99.1000
S. torquatus	UMMZ	99032	México	Distrito Federal	19.2660	-99.0500
S. torquatus	FMNH	32741-743	México	Morelos	18.8000	-99.2160
S. torquatus	TCWC	52964	México	Guanajuato	20.2200	-101.1800
S. torquatus	CIB-UAEH	CIB-UAEH-511	México	Hidalgo	20.0300	-98.8420
S. torquatus	CIB-UAEH	CIB-UAEH-1339	México	Hidalgo	20.7400	-99.3820
S. torquatus	ENCB	10716	México	Hidalgo	20.7360	-99.5340
S. torquatus	MZFC	6792	México	Hidalgo	20.7920	-99.4080
S. torquatus	MZFC	8424	México	Hidalgo	20.8431	-99.2547
S. torquatus	MZFC	9600	México	Hidalgo	20.8100	-99.2570
S. torquatus	MVZ	78282	México	Hidalgo	20.8430	-99.2540
S. torquatus	MVZ	146914	México	Hidalgo	20.8700	-99.2350
S. torquatus	ND	S/N / 6649	México	Hidalgo	20.8830	-99.2500
S. torquatus	IUMNH	20918	México	Hidalgo	20.7660	-99.3330
S. torquatus	IUMNH	48033	México	Hidalgo	20.9000	-99.2330
S. torquatus	IUMNH	20926-929	México	Hidalgo	20.8830	-99.2830
S. torquatus	UMMZ	103329	México	Hidalgo	20.7330	-99.3000
S. torquatus	AMNH	72408	México	México	19.2830	-99.7330
S. torquatus	MZFC	11801	México	Michoacán	19.9000	-100.7000
S. torquatus	MZFC	2295	México	Michoacán	19.4660	-100.3220
S. torquatus	MZFC	MZFC-12008	México	Michoacán	19.5160	-100.2600
S. torquatus	CNAR	CNAR-IBH-16427	México	Michoacán	19.4650	-100.2700
S. torquatus	CNAR	CNAR-IBH-16430	México	Michoacán	19.5030	-100.3220
S. torquatus	CNAR	CNAR-IBH-16433	México	Michoacán	19.5100	-100.3180
S. torquatus	ND	NA / 1106	México	Michoacán	19.4860	-100.2410
S. torquatus	ND	NA / 1149	México	Michoacán	19.4040	-100.2810
S. torquatus	ND	NA / 1154	México	Michoacán	19.5090	-100.3180
S. torquatus	ND	NA / 1485	México	Michoacán	19.5230	-100.2880
S. torquatus	ND	NA / 155	México	Michoacán	19.5030	-100.3210
S. torquatus	ND	NA / 1589	México	Michoacán	19.4030	-100.3030
S. torquatus	ND	NA / 1591	México	Michoacán	19.4030	-100.2810
S. torquatus	ND	NA / 1601	México	Michoacán	19.4030	-100.3110
S. torquatus	ND	NA / 1663	México	Michoacán	19.5020	-100.3210
S. torquatus	ND	NA / 1734	México	Michoacán	19.5110	-100.2440
S. torquatus	ND	NA / 1749	México	Michoacán	19.5090	-100.2560
S. torquatus	ND	NA / 1791	México	Michoacán	19.5180	-100.2510
S. torquatus	ND	NA / 1906	México	Michoacán	19.4860	-100.3260
S. torquatus	ND	NA / 1907	México	Michoacán	19.5180	-100.2840
S. torquatus	ND	NA / 1983	México	Michoacán	19.3910	-100.2830
S. torquatus	ND	NA / 1985	México	Michoacán	19.4040	-100.3070
S. torquatus	ND	NA / 1986	México	Michoacán	19.4040	-100.3040
S. torquatus	ND	NA / 2166	México	Michoacán	19.5180	-100.3100
S. torquatus	ND	NA / 2224	México	Michoacán	19.4570	-100.3400
S. torquatus	ND	NA / 2238	México	Michoacán	19.4520	-100.3470
S. torquatus	ND	NA / 2245	México	Michoacán	19.4800	-100.2620
S. torquatus	ND	NA / 2264	México	Michoacán	19.4800	-100.3250
S. torquatus	ND	NA / 2265	México	Michoacán	19.4790	-100.3260
S. torquatus	ND	NA / 2266	México	Michoacán	19.4780	-100.3290
S. torquatus	ND	NA / 2267	México	Michoacán	19.4780	-100.3280
S. torquatus	ND	NA / 564	México	Michoacán	19.4060	-100.3090
S. torquatus	ND	NA / 581	México	Michoacán	19.4040	-100.3080
S. torquatus	LACM	61985	México	Michoacán	19.4590	-100.3260
S. torquatus	MZFC	3339	México	Veracruz	20.8000	-98.3100
S. torquatus	USNM	S/N / 10041	México	México	19.8000	-99.1000
