# Supplementary material for: Climatic niche evolution in the viviparous Sceloporus torquatus group (Squamata: Phrynosomatidae)
Source: PeerJ. 2019 Jan 9;6:e6192. doi: 10.7717/peerj.6192 (PMC6330044; doi:10.7717/peerj.6192)
Supplement: Supplemental Information 4 [file peerj-07-6192-s004.docx]

| Bioclimatic layer | Model | α/a/δ | | σ^2^ | z0 | |
| --- | --- | --- | --- | --- | --- | --- |
| Mean Diurnal Range (Bio2) | BM | - | 52.619 | | | 150.704 |
|  | δ | 2.364 | 36.160 | | | 149.573 |
|  | OU | 0.120 | 100.889 | | | 149.680 |
|  | EB | -0.000001 | 52.619586 | | | 150.704 |
| Max Temperature of Warmest Month (Bio5) | OU | 2.718 | 5199.010 | | | 298.586 |
|  | δ | 2.99 | 103.748 | | | 297.978 |
|  | BM | - | 181.224 | | | 299.040 |
|  | EB | -0.000001 | 181.226 | | | 299.040 |
| Mean Temperature of Wettest Quarter (Bio8) | OU | 2.718 | 6486.571 | | | 207.486 |
|  | δ | 2.999 | 130.922 | | | 205.429 |
|  | BM | - | 235.846 | | | 205.433 |
|  | EB | -0.000001 | 235.848 | | | 205.433 |
| Mean Temperature of Driest Quarter (Bio9) | OU | 0.356 | 819.176 | | | 161.855 |
|  | δ | 2.999 | 118.711 | | | 163.208 |
|  | BM | - | 201.580 | | | 165.909 |
|  | EB | -0.000001 | 201.582 | | | 165.909 |
| Precipitation Seasonality (Bio15) | BM | - | 26.109 | | | 91.411 |
|  | δ | 1.686 | 20.219 | | | 90.565 |
|  | OU | 0.049 | 35.008 | | | 90.759 |
|  | EB | -0.000001 | 26.109 | | | 91.411 |
| Precipitation of Warmest Quarter (Bio18) | OU | 0.314 | 6234.280 | | | 270.202 |
|  | δ | 2.999 | 1011.857 | | | 273.743 |
|  | BM | - | 1675.153 | | | 281.655 |
|  | EB | -0.000001 | 1675.166 | | | 281.655 |
| Precipitation of Coldest Quarter (Bio19) | OU | 0.597 | 704.630 | | | 53.367 |
|  | δ | 2.999 | 66.327 | | | 51.680 |
|  | BM | - | 122.448 | | | 51.067 |
|  | EB | -0.000001 | 122.449 | | | 51.067 |
| Average Potential Evapo-Transpiration in May (PET5) | BM | - | 41.903 | | | 178.890 |
|  | δ | 2.999 | 27.044 | | | 177.303 |
|  | OU | 0.256 | 139.648 | | | 177.021 |
|  | EB | -0.000001 | 41.904 | | | 178.890 |
| Average precipitation in May (Prec5) | OU | 0.310 | 512.839 | | | 48.220 |
|  | δ | 2.999 | 85.395 | | | 46.636 |
|  | BM | - | 142.771 | | | 43.402 |
|  | EB | -0.000001 | 142.772 | | | 43.402 |
| Average precipitation in October (Prec10) | OU | 0.356 | 761.883 | | | 65.768 |
|  | δ | 2.999 | 110.535 | | | 65.282 |
|  | BM | - | 189.637 | | | 64.651 |
|  | EB | -0.000001 | 189.638 | | | 64.651 |
| Average maximum temperature in January (Tmax1) | BM | - | 241.711 | | | 215.719 |
|  | δ | 2.782 | 157.124 | | | 213.726 |
|  | OU | 0.176 | 592.784 | | | 213.443 |
|  | EB | -0.000001 | 241.713 | | | 215.719 |

-
