# Supplementary material for: Climatic niche evolution in the viviparous Sceloporus torquatus group (Squamata: Phrynosomatidae)
Source: PeerJ. 2019 Jan 9;6:e6192. doi: 10.7717/peerj.6192 (PMC6330044; doi:10.7717/peerj.6192)
Supplement: Supplemental Information 5 [file peerj-07-6192-s005.docx]

| Mean Diurnal Range (Bio2) | | | | | |
| --- | --- | --- | --- | --- | --- |
|  | **Mean** | **SD** | **Naive SE** | **Time-series SE** | **Effective Size** |
| lnL | -100.522 | 0.784 | 0.002 | 0.008 | 10751.171 |
| Prior | -53.321 | 8.713 | 0.028 | 0.143 | 3688.153 |
| Alpha | 0.349 | 0.723 | 0.002 | 0.027 | 740.967 |
| sig2 | 266.564 | 532.186 | 1.683 | 19.391 | 753.262 |
| K | 9.051 | 3.211 | 0.010 | 0.053 | 3732.189 |
| ntheta | 10.051 | 3.211 | 0.010 | 0.053 | 3732.189 |
| Root | 147.338 | 1.315 | 0.004 | 0.045 | 860.141 |
| all theta | 147.293 | 1.360 | NA | NA | NA |
| **Max Temperature of Warmest Month (Bio5)** | | | | | |
| lnL | -112.420 | 0.882 | 0.004 | 0.021 | 1711.315 |
| Prior | -57.148 | 8.640 | 0.035 | 0.171 | 2552.149 |
| Alpha | 0.744 | 1.277 | 0.005 | 0.051 | 631.554 |
| sig2 | 1446.506 | 2585.409 | 10.555 | 98.600 | 687.553 |
| K | 9.077 | 3.135 | 0.013 | 0.057 | 3018.018 |
| ntheta | 10.077 | 3.135 | 0.013 | 0.057 | 3018.018 |
| Root | 298.532 | 1.250 | 0.005 | 0.048 | 679.984 |
| all theta | 298.536 | 1.344 | NA | NA | NA |
| **Mean Temperature of Wettest Quarter (Bio8)** | | | | | |
| lnL | -121.547 | 0.967 | 0.003 | 0.012 | 6594.543 |
| Prior | -55.001 | 8.425 | 0.027 | 0.126 | 4491.106 |
| Alpha | 0.037 | 0.033 | 0.000 | 0.001 | 3169.032 |
| sig2 | 418.868 | 183.328 | 0.580 | 2.749 | 4446.742 |
| K | 9.026 | 3.203 | 0.010 | 0.051 | 3944.641 |
| Ntheta | 10.026 | 3.203 | 0.010 | 0.051 | 3944.641 |
| Root | 147.581 | 1.282 | 0.004 | 0.041 | 989.369 |
| all theta | 147.447 | 1.352 | NA | NA | NA |
| **Mean Temperature of Driest Quarter (Bio9)** | | | | | |
| lnL | -114.354 | 0.816 | 0.003 | 0.010 | 6185.168 |
| Prior | -56.845 | 8.680 | 0.027 | 0.137 | 4033.584 |
| Alpha | 0.569 | 1.035 | 0.003 | 0.037 | 794.645 |
| sig2 | 1333.205 | 2384.507 | 7.540 | 88.687 | 722.905 |
| K | 9.042 | 3.195 | 0.010 | 0.052 | 3827.220 |
| ntheta | 10.042 | 3.195 | 0.010 | 0.052 | 3827.220 |
| Root | 161.970 | 1.310 | 0.004 | 0.042 | 988.528 |
| all theta | 161.910 | 1.361 | NA | NA | NA |

S3. Table. Continuation.

| Precipitation Seasonality (Bio15) | | | | | |
| --- | --- | --- | --- | --- | --- |
|  | **Mean** | **SD** | **Naive SE** | **Time-series SE** | **Effective Size** |
| lnL | -93.168 | 0.933 | 0.003 | 0.017 | 2935.402 |
| prior | -50.858 | 8.538 | 0.027 | 0.131 | 4248.623 |
| Alpha | 0.116 | 0.284 | 0.001 | 0.009 | 1038.650 |
| sig2 | 60.393 | 129.302 | 0.409 | 4.259 | 921.770 |
| K | 9.087 | 3.217 | 0.010 | 0.055 | 3365.262 |
| Ntheta | 10.087 | 3.217 | 0.010 | 0.055 | 3365.262 |
| root | 85.683 | 1.269 | 0.004 | 0.042 | 894.972 |
| all theta | 85.518 | 1.346 | NA | NA | NA |
| **Precipitation of Warmest Quarter (Bio18)** | | | | | |
| lnL | -106.814 | 0.939 | 0.003 | 0.015 | 4036.695 |
| Prior | -56.750 | 8.640 | 0.027 | 0.129 | 4452.522 |
| Alpha | 0.832 | 1.491 | 0.005 | 0.054 | 774.834 |
| sig2 | 1001.151 | 1881.778 | 5.951 | 70.008 | 722.511 |
| K | 9.144 | 3.168 | 0.010 | 0.047 | 4485.922 |
| Ntheta | 10.144 | 3.168 | 0.010 | 0.047 | 4485.922 |
| Root | 53.567 | 1.270 | 0.004 | 0.040 | 1024.933 |
| all theta | 53.639 | 1.345 | NA | NA | NA |
| **Precipitation of Coldest Quarter (Bio19)** | | | | | |
| lnL | -106.867 | 0.981 | 0.003 | 0.019 | 2599.962 |
| Prior | -56.341 | 8.694 | 0.030 | 0.163 | 2858.763 |
| Alpha | 0.813 | 1.312 | 0.005 | 0.045 | 839.024 |
| sig2 | 979.732 | 1682.600 | 5.771 | 57.668 | 851.309 |
| K | 8.996 | 3.151 | 0.011 | 0.054 | 3387.888 |
| Ntheta | 9.996 | 3.151 | 0.011 | 0.054 | 3387.888 |
| Root | 53.625 | 1.311 | 0.004 | 0.048 | 753.963 |
| all theta | 53.661 | 1.365 | NA | NA | NA |
| **Average Potential Evapo-Transpiration in May (PET5)** | | | | | |
| lnL | -97.474 | 0.764 | 0.003 | 0.008 | 9958.277 |
| Prior | -53.612 | 8.676 | 0.029 | 0.147 | 3472.218 |
| Alpha | 0.511 | 1.301 | 0.004 | 0.052 | 627.131 |
| sig2 | 281.985 | 705.200 | 2.351 | 28.053 | 631.910 |
| K | 9.139 | 3.169 | 0.011 | 0.053 | 3601.551 |
| ntheta | 10.139 | 3.169 | 0.011 | 0.053 | 3601.551 |
| Root | 176.663 | 1.277 | 0.004 | 0.041 | 976.308 |
| all theta | 176.597 | 1.355 | NA | NA | NA |

S3. Table. Continuation.

| Average precipitation in May (Prec5) | | | | | |
| --- | --- | --- | --- | --- | --- |
|  | **Mean** | **SD** | **Naive SE** | **Time-series SE** | **Effective Size** |
| lnL | -110.561 | 0.838 | 0.003 | 0.012 | 4790.376 |
| Prior | -56.151 | 8.610 | 0.029 | 0.155 | 3088.884 |
| Alpha | 0.553 | 0.936 | 0.003 | 0.031 | 928.014 |
| sig2 | 944.689 | 1672.337 | 5.574 | 53.408 | 980.460 |
| K | 9.069 | 3.154 | 0.011 | 0.056 | 3194.923 |
| ntheta | 10.069 | 3.154 | 0.011 | 0.056 | 3194.923 |
| Root | 49.866 | 1.286 | 0.004 | 0.044 | 845.707 |
| all theta | 49.937 | 1.350 | NA | NA | NA |
| **Average precipitation in October (Prec10)** | | | | | |
| lnL | -113.686 | 0.844 | 0.003 | 0.012 | 4715.943 |
| Prior | -56.812 | 8.661 | 0.029 | 0.140 | 3824.559 |
| Alpha | 0.542 | 0.870 | 0.003 | 0.032 | 740.863 |
| sig2 | 1207.982 | 1987.411 | 6.625 | 73.093 | 739.299 |
| K | 9.119 | 3.203 | 0.011 | 0.055 | 3427.403 |
| Ntheta | 10.119 | 3.203 | 0.011 | 0.055 | 3427.403 |
| Root | 66.638 | 1.300 | 0.004 | 0.048 | 746.002 |
| all theta | 66.671 | 1.347 | NA | NA | NA |
| **Average maximum temperature in January (Tmax1)** | | | | | |
| lnL | -117.772 | 0.744 | 0.002 | 0.006 | 16078.420 |
| prior | -56.649 | 8.724 | 0.029 | 0.156 | 3113.841 |
| alpha | 0.389 | 0.917 | 0.003 | 0.026 | 1223.052 |
| sig2 | 1293.245 | 2941.536 | 9.805 | 85.277 | 1189.836 |
| k | 9.097 | 3.204 | 0.011 | 0.055 | 3384.599 |
| ntheta | 10.097 | 3.204 | 0.011 | 0.055 | 3384.599 |
| root | 212.149 | 1.286 | 0.004 | 0.042 | 933.477 |
| all theta | 212.138 | 1.349 | NA | NA | NA |
