# Supplementary material for: Climatic niche evolution in the viviparous Sceloporus torquatus group (Squamata: Phrynosomatidae)
Source: PeerJ. 2019 Jan 9;6:e6192. doi: 10.7717/peerj.6192 (PMC6330044; doi:10.7717/peerj.6192)
Supplement: Supplemental Information 6 [file peerj-07-6192-s006.doc]

| ID | Species | ID | | | | | | | | | | | | | | | | | | | |
| --- | --- | --- | --- | --- | --- | --- | --- | --- | --- | --- | --- | --- | --- | --- | --- | --- | --- | --- | --- | --- | --- |
|  |  | 1 | 2 | 3 | 4 | 5 | 6 | 7 | 8 | 9 | 10 | 11 | 12 | 13 | 14 | 15 | 16 | 17 | 18 | 19 | 20 |
| **1** | *Sceloporus aureolus* | **NA** | 0.146 | 0.321 | 0.060 | 0.059 | 0.098 | 0.195 | 0.351 | 0.033 | 0.139 | 0.131 | 0.476 | 0.117 | 0.290 | 0.101 | 0.002 | 0.443 | 0.157 | 0.407 | 0.368 |
| **2** | *Sceloporus binocularis* | 0.341 | **NA** | 0.184 | 0.262 | 0.577 | 0.325 | 0.118 | 0.179 | 0.158 | 0.252 | 0.362 | 0.234 | 0.731 | 0.073 | 0.477 | 0.032 | 0.137 | 0.118 | 0.186 | 0.256 |
| **3** | *Sceloporus bulleri* | 0.613 | 0.427 | **NA** | 0.077 | 0.085 | 0.112 | 0.432 | 0.594 | 0.122 | 0.286 | 0.192 | 0.313 | 0.145 | 0.310 | 0.128 | 0.007 | 0.200 | 0.130 | 0.370 | 0.326 |
| **4** | *Sceloporus ornatus caeruleus* | 0.175 | 0.567 | 0.219 | **NA** | 0.264 | 0.670 | 0.083 | 0.142 | 0.226 | 0.167 | 0.158 | 0.103 | 0.221 | 0.033 | 0.611 | 0.069 | 0.056 | 0.033 | 0.101 | 0.111 |
| **5** | *Sceloporus cyanogenys* | 0.168 | 0.838 | 0.253 | 0.518 | **NA** | 0.269 | 0.065 | 0.089 | 0.099 | 0.178 | 0.266 | 0.118 | 0.533 | 0.019 | 0.427 | 0.005 | 0.060 | 0.061 | 0.081 | 0.140 |
| **6** | *Sceloporus cyanostitctus* | 0.237 | 0.614 | 0.273 | 0.907 | 0.553 | **NA** | 0.100 | 0.210 | 0.232 | 0.155 | 0.134 | 0.116 | 0.287 | 0.074 | 0.673 | 0.069 | 0.083 | 0.045 | 0.160 | 0.114 |
| **7** | *Sceloporus dugesii* | 0.452 | 0.325 | 0.735 | 0.236 | 0.202 | 0.264 | **NA** | 0.432 | 0.060 | 0.376 | 0.175 | 0.233 | 0.086 | 0.198 | 0.103 | 0.002 | 0.081 | 0.047 | 0.261 | 0.294 |
| **8** | *Sceloporus insignis* | 0.638 | 0.415 | 0.856 | 0.327 | 0.233 | 0.399 | 0.745 | **NA** | 0.116 | 0.362 | 0.209 | 0.289 | 0.135 | 0.395 | 0.180 | 0.004 | 0.246 | 0.117 | 0.515 | 0.324 |
| **9** | *Sceloporus jarrovii* | 0.159 | 0.397 | 0.320 | 0.458 | 0.288 | 0.452 | 0.185 | 0.294 | **NA** | 0.135 | 0.097 | 0.057 | 0.123 | 0.017 | 0.174 | 0.204 | 0.026 | 0.020 | 0.106 | 0.055 |
| **10** | *Sceloporus melanogaster* | 0.414 | 0.545 | 0.572 | 0.402 | 0.384 | 0.403 | 0.674 | 0.669 | 0.366 | **NA** | 0.500 | 0.269 | 0.198 | 0.100 | 0.209 | 0.007 | 0.084 | 0.038 | 0.285 | 0.460 |
| **11** | *Sceloporus minor* | 0.349 | 0.645 | 0.425 | 0.405 | 0.536 | 0.380 | 0.429 | 0.468 | 0.290 | 0.784 | **NA** | 0.288 | 0.298 | 0.054 | 0.215 | 0.005 | 0.078 | 0.063 | 0.190 | 0.489 |
| **12** | *Sceloporus mucronatus* | 0.768 | 0.485 | 0.605 | 0.307 | 0.298 | 0.318 | 0.507 | 0.583 | 0.258 | 0.558 | 0.567 | **NA** | 0.180 | 0.154 | 0.147 | 0.010 | 0.242 | 0.102 | 0.453 | 0.621 |
| **13** | *Sceloporus oberon* | 0.292 | 0.937 | 0.363 | 0.510 | 0.820 | 0.565 | 0.254 | 0.341 | 0.332 | 0.478 | 0.582 | 0.417 | **NA** | 0.050 | 0.438 | 0.020 | 0.135 | 0.130 | 0.140 | 0.199 |
| **14** | *Scelporus omiltemanus* | 0.576 | 0.207 | 0.586 | 0.107 | 0.068 | 0.187 | 0.458 | 0.671 | 0.090 | 0.273 | 0.138 | 0.356 | 0.158 | **NA** | 0.058 | 0.001 | 0.252 | 0.069 | 0.378 | 0.158 |
| **15** | *Sceloporus ornatus ornatus* | 0.273 | 0.788 | 0.323 | 0.855 | 0.717 | 0.894 | 0.272 | 0.386 | 0.428 | 0.445 | 0.495 | 0.386 | 0.753 | 0.178 | *NA* | 0.056 | 0.102 | 0.082 | 0.128 | 0.152 |
| **16** | *Sceloporus poinsettii* | 0.013 | 0.121 | 0.033 | 0.190 | 0.036 | 0.201 | 0.008 | 0.018 | 0.475 | 0.053 | 0.048 | 0.064 | 0.091 | 0.003 | 0.187 | **NA** | 0.001 | 0.001 | 0.008 | 0.009 |
| **17** | *Sceloporus prezygus* | 0.759 | 0.341 | 0.448 | 0.156 | 0.177 | 0.205 | 0.233 | 0.499 | 0.130 | 0.294 | 0.269 | 0.534 | 0.321 | 0.514 | 0.276 | 0.016 | **NA** | 0.222 | 0.280 | 0.167 |
| **18** | *Sceloporus serrifer* | 0.351 | 0.309 | 0.348 | 0.105 | 0.198 | 0.128 | 0.161 | 0.271 | 0.087 | 0.174 | 0.234 | 0.324 | 0.340 | 0.190 | 0.245 | 0.005 | 0.496 | **NA** | 0.085 | 0.062 |
| **19** | *Sceloporus sugillatus* | 0.673 | 0.431 | 0.642 | 0.278 | 0.220 | 0.352 | 0.539 | 0.785 | 0.280 | 0.581 | 0.425 | 0.702 | 0.358 | 0.659 | 0.335 | 0.042 | 0.533 | 0.208 | **NA** | 0.425 |
| **20** | *Sceloporus torquatus* | 0.642 | 0.532 | 0.602 | 0.321 | 0.349 | 0.337 | 0.585 | 0.607 | 0.236 | 0.723 | 0.745 | 0.847 | 0.463 | 0.357 | 0.406 | 0.068 | 0.415 | 0.220 | 0.668 | **NA** |
