# Supplementary material for: Climatic niche evolution in the viviparous Sceloporus torquatus group (Squamata: Phrynosomatidae)
Source: PeerJ. 2019 Jan 9;6:e6192. doi: 10.7717/peerj.6192 (PMC6330044; doi:10.7717/peerj.6192)
Supplement: Supplemental Information 7 [file peerj-07-6192-s007.docx]

| Variable | PC1 | PC2 | PC3 |
| --- | --- | --- | --- |
| Annual Mean Temperature(Bio1) | -0.128 | -0.342 | -0.007 |
| Mean Diurnal Range (Bio2) | 0.222 | 0.153 | 0.182 |
| Isothermality (Bio3) | -0.282 | 0.034 | 0.127 |
| Temperature Seasonality (Bio4) | 0.300 | -0.003 | -0.098 |
| Max Temperature of Warmest Month (Bio5) | 0.152 | -0.330 | -0.089 |
| Min Temperature of Coldest Month (Bio6) | -0.215 | -0.275 | -0.084 |
| Temperature Annual Range (Bio7) | 0.300 | 0.070 | 0.028 |
| Mean Temperature of Wettest Quarter (Bio8) | 0.083 | -0.357 | -0.151 |
| Mean Temperature of Driest Quarter (Bio9) | -0.128 | -0.342 | -0.007 |
| Mean Temperature of Warmest Quarter (Bio10) | 0.093 | -0.352 | -0.157 |
| Mean Temperature of Coldest Quarter (Bio11) | -0.211 | -0.284 | -0.034 |
| Annual Precipitation (Bio12) | -0.297 | 0.100 | 0.011 |
| Precipitation of Wettest Month (Bio13) | -0.288 | 0.088 | 0.106 |
| Precipitation of Driest Month (Bio14) | -0.061 | 0.105 | -0.487 |
| Precipitation Seasonality (Bio15) | -0.152 | -0.041 | 0.440 |
| Precipitation of Wettest Quarter (Bio16) | -0.282 | 0.087 | 0.141 |
| Precipitation of Driest Quarter (Bio17) | -0.065 | 0.126 | -0.484 |
| Precipitation of Warmest Quarter (Bio18) | -0.201 | 0.073 | -0.017 |
| Precipitation of Coldest Quarter (Bio19) | -0.042 | 0.129 | -0.319 |
| Average Potential Evapotranspiration in May (Pet5) | 0.191 | -0.243 | 0.066 |
| Average precipitation in May (Prec5) | -0.212 | 0.101 | -0.227 |
| Average precipitation in October (Prec10) | -0.276 | 0.073 | -0.146 |
| Average maximum temperature in January (Tmax1) | -0.219 | -0.264 | 0.041 |
| Proportion of explained variance | 43.2% | 28.1% | 15.3% |
