# Supplementary material for: Climatic niche evolution in the viviparous Sceloporus torquatus group (Squamata: Phrynosomatidae)
Source: PeerJ. 2019 Jan 9;6:e6192. doi: 10.7717/peerj.6192 (PMC6330044; doi:10.7717/peerj.6192)
Supplement: Supplemental Information 8 [file peerj-07-6192-s008.docx]

| PC | Model | lnL | AICc | Parameters |
| --- | --- | --- | --- | --- |
| PC1 | **BM** | **-58.113** | **120.825** | **2** |
|  | EB | -57.973 | 123.210 | 3 |
|  | δ (2.6) | -58.104 | 123.471 | 3 |
|  | OU | -58.113 | 123.488 | 3 |
| PC2 | **BM** | **-55.197** | **114.994** | **2** |
|  | OU | -54.166 | 115.594 | 3 |
|  | δ (2.9) | -54.233 | 115.729 | 3 |
|  | EB | -55.197 | 117.657 | 3 |
| PC3 | **δ (2.9)** | **-45.784** | **96.167** | **2** |
|  | EB | -45.782 | 98.827 | 3 |
|  | BM | -45.783 | 98.830 | 3 |
|  | OU | -45.784 | 98.831 | 3 |
