# Supplementary figures and images for: Climatic niche evolution in the viviparous Sceloporus torquatus group (Squamata: Phrynosomatidae)
Source: PeerJ. 2019 Jan 9;6:e6192. doi: 10.7717/peerj.6192 (PMC6330044; doi:10.7717/peerj.6192)

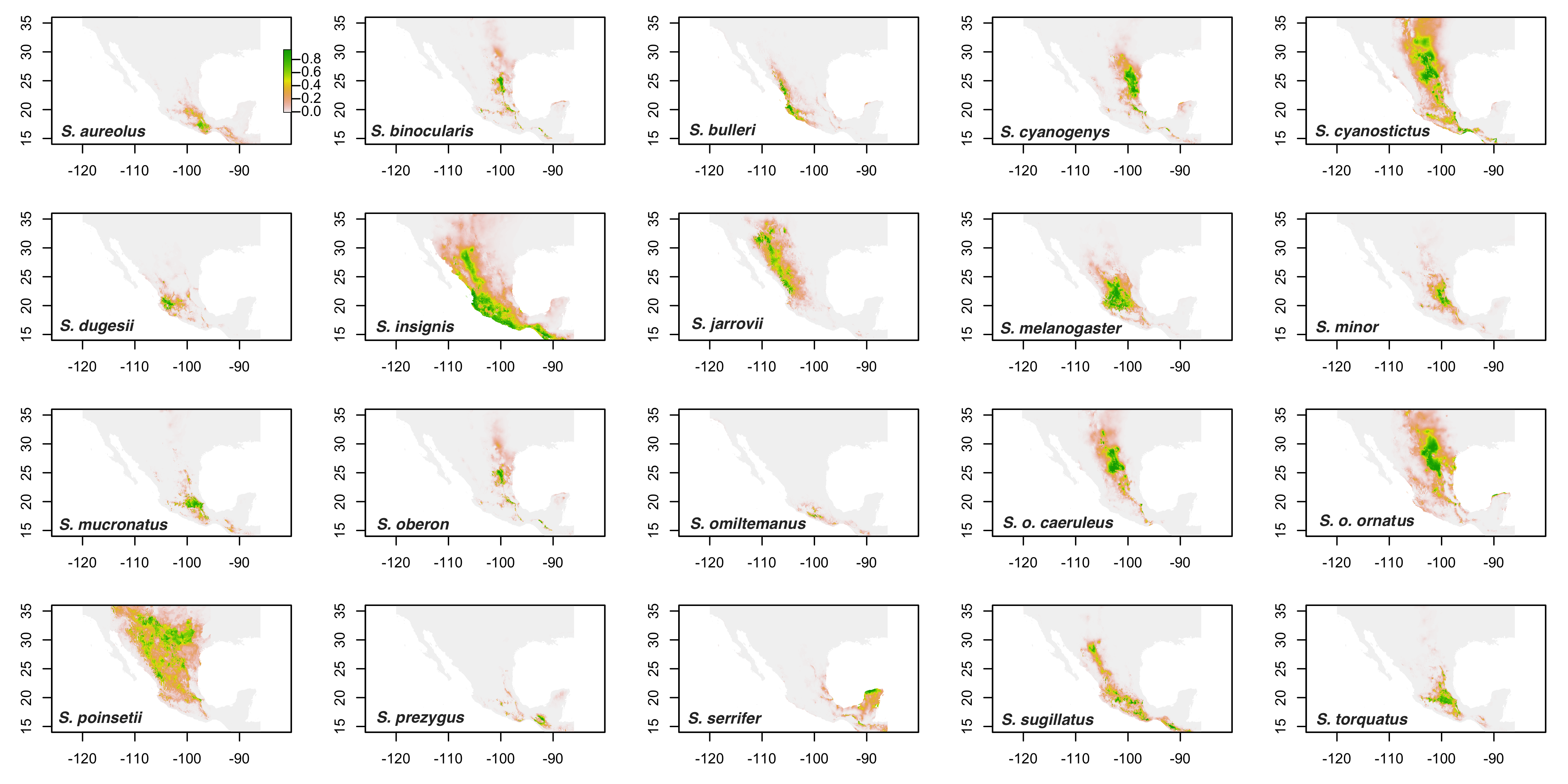

Supplement: Supplemental Information 9 [file peerj-07-6192-s009.png]

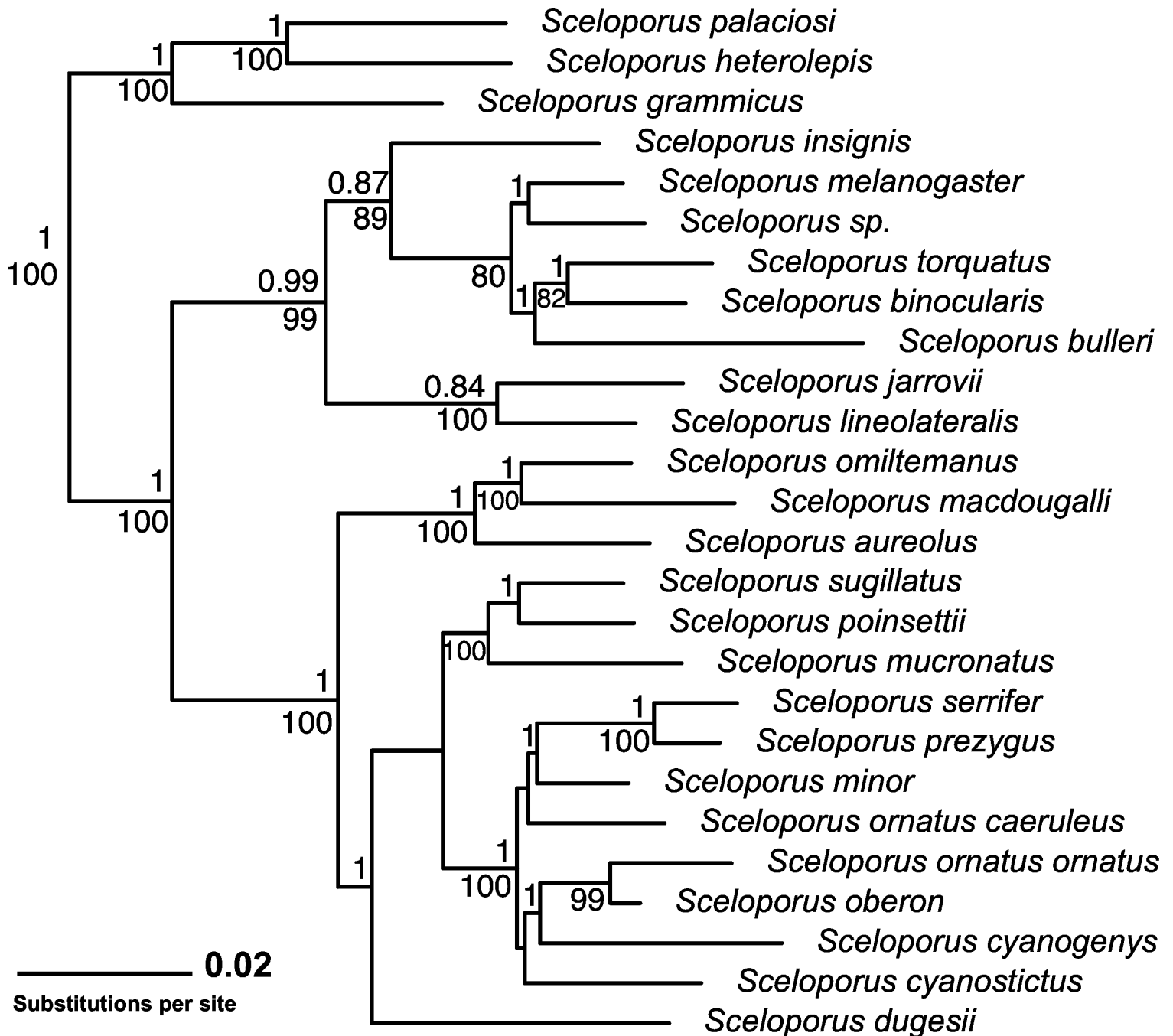

Supplement: Supplemental Information 10 — Nodes with posterior probability values = 0.5 and bootstrap values = 50% are shown. [file peerj-07-6192-s010.pdf]

**Tmax1**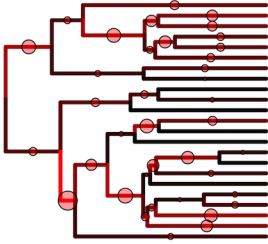**PET5**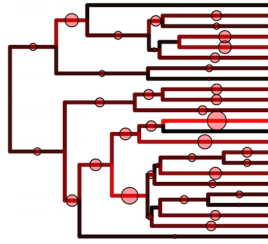**Prec5**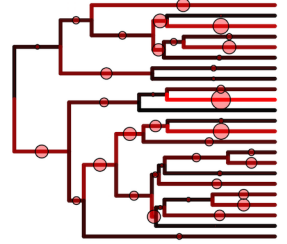**Prec10**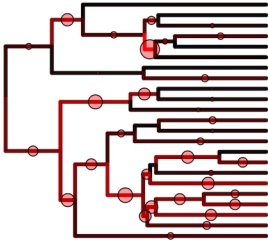**Bio2**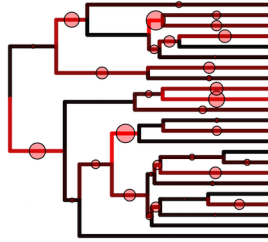**Bio5**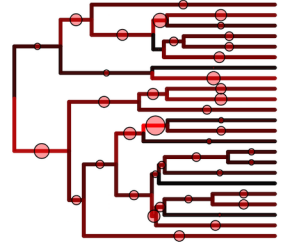**Bio8**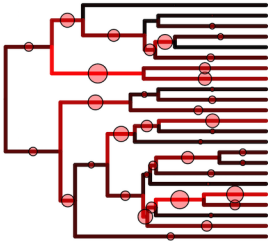**Bio9**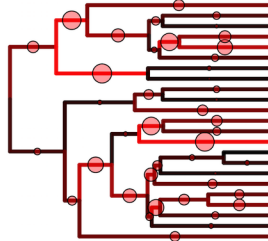**Bio15**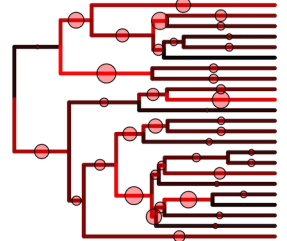**Bio18**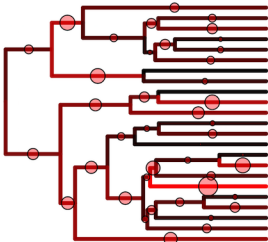**Bio19**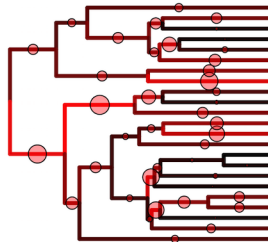

● pp = 0.25

Supplement: Supplemental Information 11 — The diameter of the circles at branches is proportional to the posterior probability of shift. [file peerj-07-6192-s011.pdf]

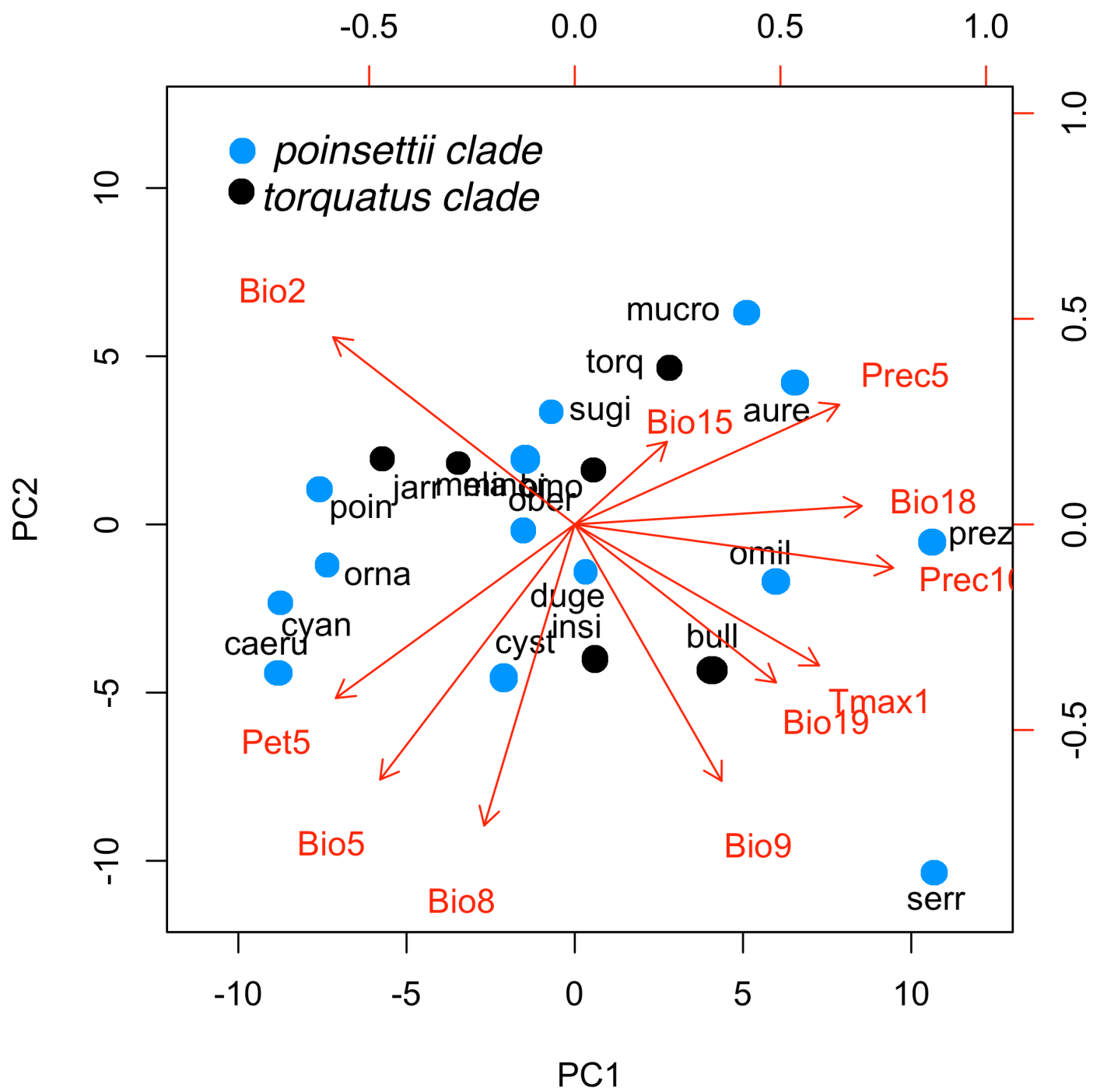

Supplement: Supplemental Information 12 — The arrows are the loadings that indicate the direction and strength of each environmental variable to the overall distribution. The percentages of variance explained by the first two PCs are in the axis labels. Species names consisting of the four letters of the species epithets, except for Sceloporus ornatus caeruleos (caeru). [file peerj-07-6192-s012.pdf]

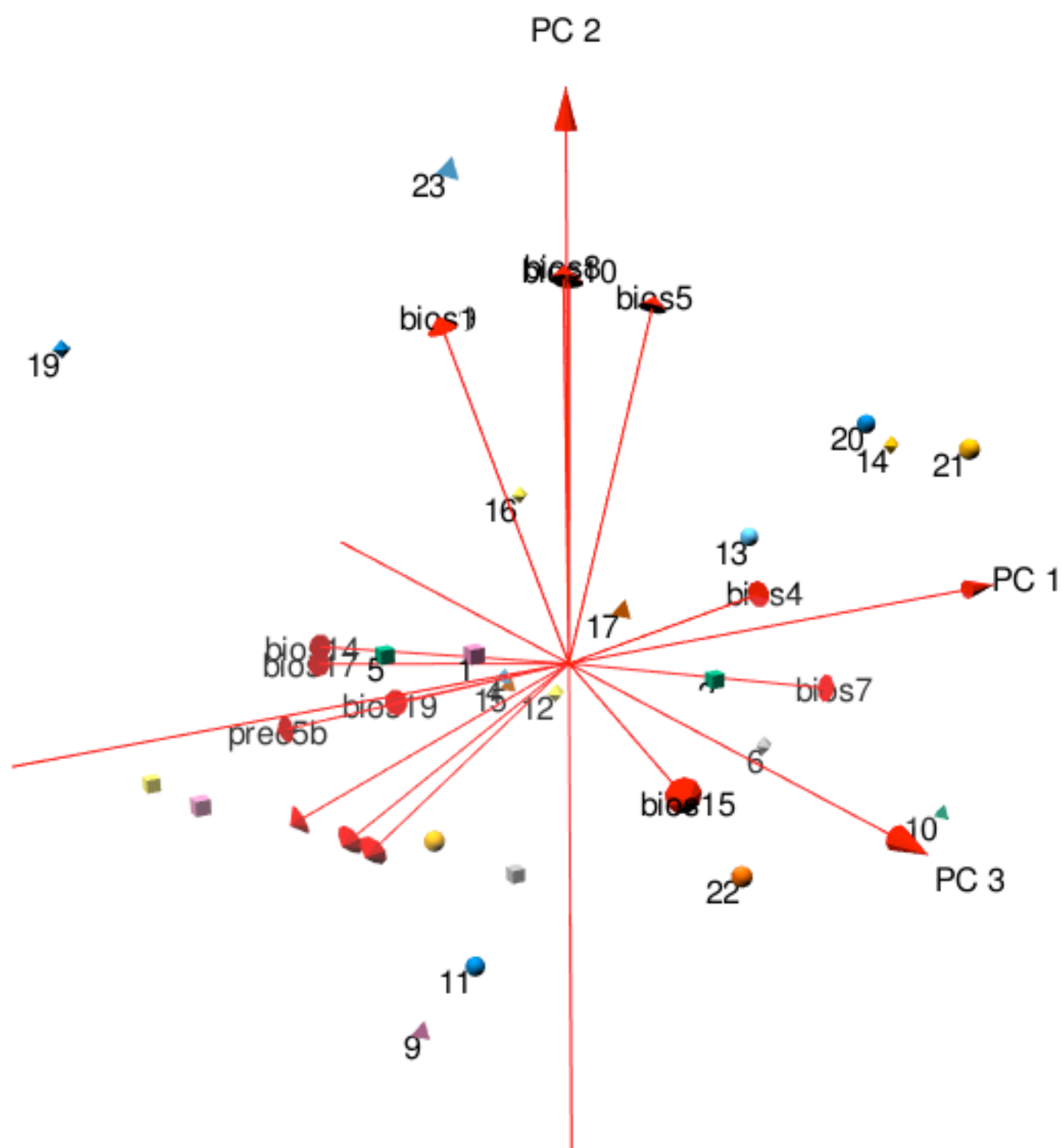

Supplement: Supplemental Information 13 — PCA Lebels: 1. Sceloporus aureolus, 2. S. binocularis, 3. S. bulleri, 4. S. cyanogenys, 5. S. cyanostictus, 6. S. dugesii, 7 S. insignis, 8. S. jarrovii, 9. S. lineolateralis, 10. S. macdougalli, 11. S. melanogaster, 12. S. minor, 13. S. mucronatus, 14. S. oberon, 15. S. omiltemanus, 16. S. ornatus caeruleus, 17. S. ornatus ornatus, 18. S. poinsettii, 19. S. prezygus, 20. S. serrifer, 21. Sceloporus sp., 22. S. sugillatus, 23. S. torquatus. [file peerj-07-6192-s013.pdf]

PC1

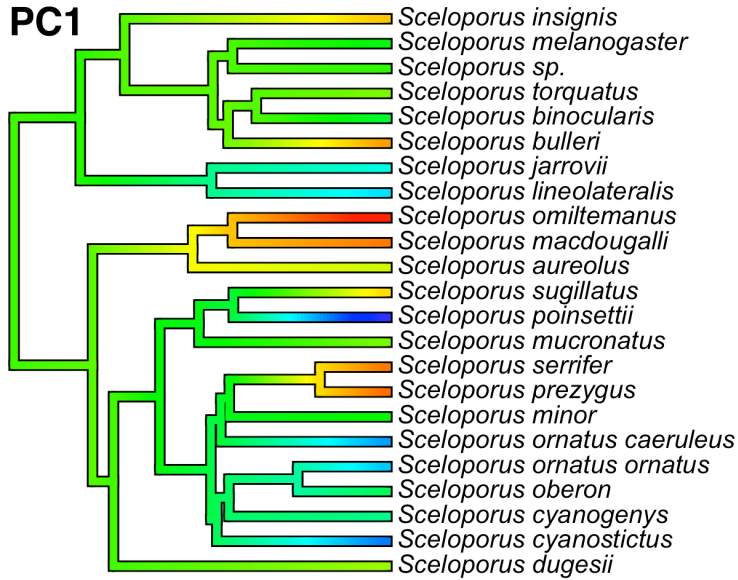

-5.214 trait value 6.15

length=5.177

PC2

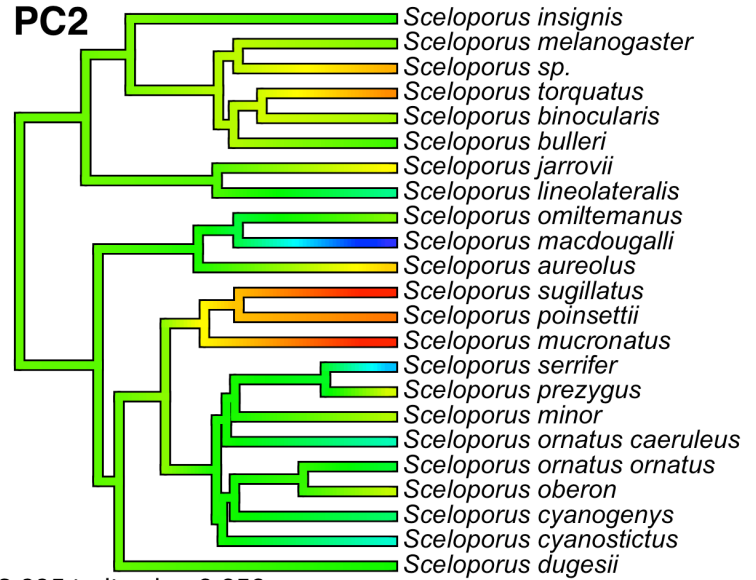

-3.995 trait value 6.656

length=5.177

PC3

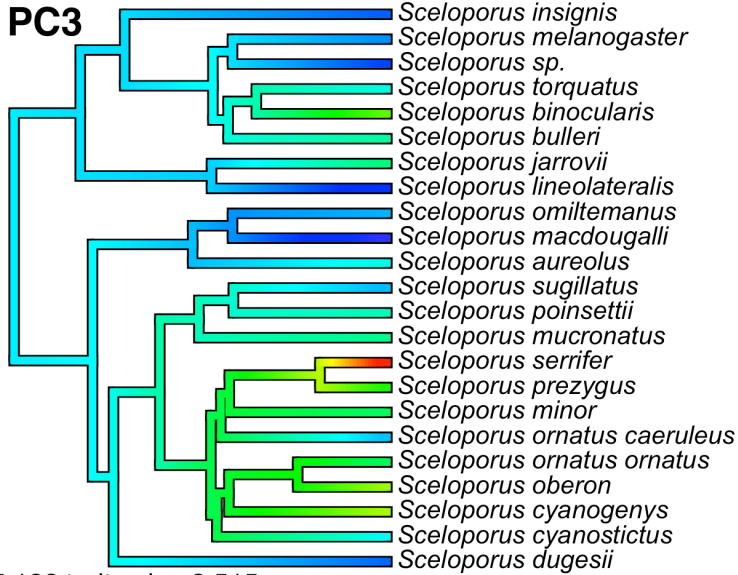

-5.122 trait value 2.515

length=5.177

Supplement: Supplemental Information 14 [file peerj-07-6192-s014.pdf]
